# Inhibition of Metal–Support Interactions by Rare-Earth Doping in Palladium/Ceria Zirconia Three-Way Catalysts

**DOI:** 10.1021/acs.chemmater.5c01417

**Published:** 2025-08-29

**Authors:** Lucy Costley-Wood, Nicolás A. Flores-González, Claire Wilson, Paul Thompson, Sarah Day, Veronica Celorrio, Donato Decarolis, Ruby Morris, Manfred E. Schuster, Huw Marchbank, Timothy I. Hyde, Amy Kolpin, Dave Thompsett, Emma K. Gibson

**Affiliations:** † 3526 University of Glasgow, Glasgow G12 8QQ, U.K.; ‡ University College London, London WC1H 0AJ, U.K.; § Faculty of Engineering, University of Concepción, Concepción 4070386, Chile; ∥ ESRF—The European Synchrotron, Grenoble 38000, France; ⊥ 120796 Diamond Light Source, Didcot OX11 0DE, U.K.; # Johnson Matthey Technology Centre, Reading RG4 9NH, U.K.

## Abstract

The impact of rare-earth (RE) doping in ceria-zirconia—critical for enhancing thermal stability and optimizing redox properties—on surface palladium (Pd) behavior has been investigated. RE doping was found to weaken metal–support interactions, leading to increased Pd mobility, with notable effects on oxygen storage capacity and light-off performance under model exhaust conditions. The mobility and redox characteristics of Pd were assessed through in situ thermal experiments using X-ray absorption spectroscopy at the Pd K-edge and synchrotron powder diffraction. Complementary Ce K-edge EXAFS and Rietveld refinements confirmed the structure and composition of the doped ceria-zirconia material. Deactivation studies and lifetime prediction are essential for commercial catalysts, particularly for three-way catalysts (TWCs) designed for decade-long operation. To probe long-term stability, in situ thermal treatments were conducted to induce separation of the metastable ceria–zirconia solid solution. These accelerated thermal aging treatments were then compared with a prolonged, seven week aging protocol, and regular in situ synchrotron PXRD measurements provided insights into the phase separation process. The influence of thermal aging on metal–support interactions was further assessed through catalytic performance testing.

## Graphical abstract

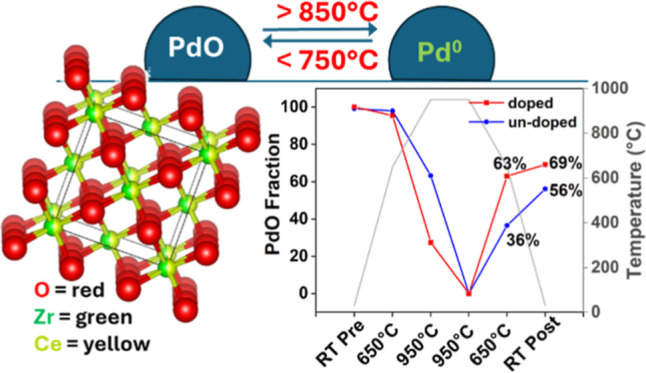

## 1 Introduction

Transport is responsible for almost a quarter of Europe’s greenhouse gas emissions and is the primary cause of air pollution in cities.^1^ Three-way catalytic converters (TWCs) are the emission control technology employed in gasoline vehicles, where the mass ratio of air-to-fuel burned is typically 14.7:1.^2^ Such internal combustion engine vehicles still make up the majority of the fleet in the UK; in 2024, 52% of all new vehicle registrations were for non-hybrid petrol cars.^3^

TWCs consist of a honeycomb structured monolith, with the catalytic components suspended in a washcoat that is applied to the monolith. Pd is employed in the majority of TWCs, either alone or, more commonly, in mixtures with Rh. Pd offers high thermal stability, fast light-off,^4,5^ and its redox activity is higher than that of Pt.^6^ Ceria zirconia (CZ) has been used as a promoter to alumina since the 1980s, helping stabilize the platinum group metal (PGM), maintaining high dispersions, and also enabling the water gas shift reaction (eq 1).^7^ This provides an additional mechanism for the removal of CO, in turn generating extra H_2_ for the reduction of NO_x_.^7^ Wang et al. published an extensive review of the various reaction pathways involved in the elimination of CO, hydrocarbons (HCs), and nitrogen oxides (NOx) on a Pd-based TWC.^8^

1 $$CO+H_{2}O\to H_{2}+{CO}_{2}$$

Ceria zirconia can also stabilize the fluctuating oxygen content in the exhaust gas, widening the operational range for both the oxidation and reduction reactions being performed.^7^ This is a result of the redox properties of Ce, known to be enhanced when in solid solutions with Zr, resulting in a superior oxygen storage capacity (OSC). The Zr is not itself reducible, but a larger stoichiometric amount of Ce^4+^ can be reduced when Zr^4+^ is in its coordination sphere.^9^ Along with modulating the morphological and electronic properties of CeO2, Zr is essential also for increasing its thermal stability.^10,11^ This is particularly relevant in gasoline applications, where exhaust temperatures are thought to reach, or even exceed, 1000 °C during acceleration events and high load operation, and vehicle lifetimes should exceed 160,000 km.^11^ Ceria zirconia can however undergo phase separation during aging processes, where two or more compositionally different crystalline phases result from one homogeneously mixed phase.^12,13^ It is noted that solid solutions of Ce and other metals including but not limited to manganese, iron, and lanthanum can also be prepared and show enhanced textural properties, redox activity, and soot oxidation performance compared to pure CeO_2_, demonstrated for example by Mukherjee et al.^14^

Rare-earth (RE) metal doping is employed to inhibit the phase separation of ceria zirconia, when dopants are added in appropriate quantities, typically yttrium,^15^ lanthanum,^16,17^ neodymium,^17,18^ and praseodymium.^15,19^ They are believed to be substitutional dopants integrating into the ceria zirconia solid solution. REs are typically trivalent; hence, their introduction creates oxygen vacancies in the structure due to the requirement for charge balancing. Therefore, despite not contributing to the total reducibility, RE doping facilitates oxygen diffusion during reduction and oxidation processes via these vacancies.^20^

The OSC of CZ is dramatically improved by the addition of Pd and other PGMs, particularly at low temperatures. This is due to the oxygen transfer via the PGM at the PGM–CZ interface, from/to lattice vacancies in the CZ.^21–25^ Oxygens migrate from the ceria zirconia via this interface, creating vacancies, and contributing to the reaction through the PGM. Recent studies, particularly those by Nagasawa et al.^21^ and by Garcia et al.,^22^ have further elucidated the crucial role of the Pd–CZ interaction during oxygen storage and release. Pd can also itself be reversibly reduced and hence can increase the total amount of releasable oxygen.^21,22^

The roles of both RE dopants and PGMs individually on the oxygen storage properties and lifetime of ceria zirconias have been well studied. Often the focus however is on either the structural characterization, or performance, and the effects of aging on one of the two. However, to the best of the authors knowledge, no research has been conducted on the effect of RE doping on the Pd–support interaction, a crucial property given that the Pd–Ce interface provides the most active sites for reaction, and is where oxygen exchange between the catalyst and the reactants occur.^22,25^ This paper aims to provide a comprehensive study on the effect of rare-earth doping on the TWC structure, OSC, and activity and extrapolate from this the effects of RE doping on the metal support interaction (MSI). The effect of thermal aging on the MSI, and subsequently on the structure and performance, will also be presented. This is conducted through in situ X-ray powder diffraction and X-ray absorption spectroscopy, coupled with OSC and activity testing under model exhaust conditions. Finally, a major and demonstrable concern of accelerated aging methods is addressed, which is that the response of a material to shorter, harsher aging conditions may be different to the response during years of actual operation.^26–28^ Long duration thermal aging methods were compared to be accelerated for these same catalysts by means of the long duration experiment (LDE) facility on the I11 powder diffraction beamline at the Diamond Light Source.^29^ The selected catalysts for all sections of the following results were 5 wt % Pd supported on two different ceria zirconias, one RE doped and one undoped. The high loading was to enhance visibility in the selected characterization methods, and, despite likely decreasing the reduction temperature of both the PdO and the support, no significant effects on the catalyst structure or behavior are expected.^30^

## 2 Materials and Methods

### 2.1 Catalyst Preparation and Aging

The ceria zirconia supports were supplied by Johnson Matthey. The two used throughout this study were a nonstabilized Ce_0.5_Zr_0.5_O_2_ (CZ1) and a RE-stabilized Ce_0.357_Zr_0.592_RE_0.051_O_2–*x*_ (CZ2), where RE = Nd^3+^ and La^3+^. The fractional occupancy of oxygen is lower in CZ2 to charge balance the trivalent dopants and hence is described by “2 – *x*”. Other formulations with Ce/Zr ratios of 3:1 and 1:5, along with a pure CeO_2_, were also obtained from Johnson Matthey, though used only to calibrate lattice parameters and Ce–Ce interatomic distances by PXRD and XAFS, respectively. These were CeO_2_, Ce_0.75_Zr_0.25_O_2_, and Ce_0.143_Zr_0.810_RE_0.046_O_2–*x*_. To prepare the Pd catalysts, 5 wt % Pd was added to all the supports by incipient wetness impregnation in a single step, from a palladium nitrate precursor, Pd­(NO_3_)_2_·*x*H_2_O. These were dried overnight at 120 °C and calcined in air at 500 °C for 2 h. Samples described as “aged” underwent high-temperature calcinations in an open tubular furnace; typical conditions were 950 °C at 10 °C min^–1^ for 12 h, and if other conditions were used then these are specified in the relevant sections.

### 2.2 Nitrogen Sorption Measurements

Surface area, pore size, and pore volume were measured by nitrogen sorption at 77 K, using a Quadrasorb Evo Gas Sorption Surface Area and Pore Analyzer. Degassing was performed at 120 °C for 17 h under vacuum. The multipoint BET method was applied to determine surface area, and the BJH method was applied to the desorption branch of the isotherm for pore volume.

### 2.3 Transmission Electron Microscopy (TEM) and Energy-Dispersive X-ray Spectrometry (EDS)

TEM was performed on Johnson Matthey’s JEOL-ARM2000 aberration-corrected instrument located at the ePSIC center, using a 200 keV beam. Imaging was in the bright-field mode using an annular detector. Powder samples were ground between glass slides and then dusted onto a holey carbon coated Cu TEM grid. Compositional analysis by X-ray emission detection was performed in the scanning mode using a Gatan GIF detector. Images were processed and analyzed using Gatan Microscopy Suite software and by ImageJ.^31^

### 2.4 Laboratory PXRD

Ex situ powder X-ray diffraction was performed on a PANalytical Empyrean diffractometer using a Cu source, with a monochromator removing Kα_2_ radiation. Structure refinements were performed by the Rietveld method using GSAS II.^32,33^ In situ experiments were performed using a PANalytical X’Pert Pro, with an Anton Paar HTK1200N chamber. Experiments were performed in an atmospheric air. Other details of the full structure refinements can be found in Supporting Information Section S1, including refinement calibrations using various Ce_*x*_Zr_1–*x*_O_2_ formulations to ensure high-quality fits. Further refinement details relating to specific data sets are signposted at the relevant Results sections.

### 2.5 Long Duration Powder Diffraction Experiments on I11, Diamond Light Source

This was performed at the long duration^29^ experiment (LDE) facility on the I11 beamline at the Diamond Light source.^29,34^ Pellets of each Pd/CZ1 and Pd/CZ2 were held at 850 °C in Linkam cells for 7 or more weeks. Powder diffraction patterns were collected in situ once a week by moving the stage into the path of the beam. Measurements were at 25 keV using a Pixium area detector over 50 s. Further details, including data and temperature calibrations, can be found in Supporting Information Section S2.

### 2.6 X-ray Absorption Spectroscopy Experiments

Ex situ data collection at the Pd K edge and Ce L_3_ edges was performed on B18 at the Diamond Light Source via UK Catalysis Hub Block Allocation Group Access. Data were collected in the transmission mode at the Pd edge and the fluorescence mode at the Ce edge, with 3 scans merged per sample during processing. Ex situ data collection at the Ce K edge was performed in the fluorescence mode at the XMaS beamline at the ESRF. Further details of data correction and processing of the Ce K edge data are found in Supporting Information Section S3.

An in situ XANES experiment was performed on B18 at the Diamond Light Source, also via UK Catalysis Hub Block Allocation Group Access. Samples were measured at the Pd K edge, in the fluorescence mode, using a capillary furnace open to air. The Pd-loaded samples were heated to 950 °C at 10 °C min^–1^, held for 2 h, and then cooled at the same rate. Isothermal points were added at 650 °C, a temperature of specific interest. XANES spectra were collected throughout. Processing of all XAS data including spectral merging, calibration, normalization, linear combination fitting, and EXAFS fitting was performed using Athena and Artemis from the Demeter software package.^34^

### 2.7 Combined Diffraction and Absorption Spectroscopy Experiment

A combined PXRD (24 keV) and Pd K edge XANES experiment was performed on the XMaS beamline (BM28) at the ESRF to determine the PdO decomposition and reoxidation temperatures and compare the values obtained by the two different techniques. Powder samples were heated to 900 °C, held at temperature for 1 h, and cooled, at 10 °C min^–1^. A custom-made high-temperature cell was used, described along with other relevant experimental details in Supporting Information Section S4.

### 2.8 Oxygen Storage Testing

Two methods of OSC testing were applied: bare ceria zirconias were tested using a dynamic method, and Pd-loaded ceria zirconias were tested using a breakthrough method. The requirement for this is due to the multiple CO oxidation pathways present for Pd/CZ, demonstrated with reaction data in Supporting Information Section S5. For both methods, the sample (sieve fraction 250–350 μm) was loaded into a quartz capillary and plugged with quartz wool, and testing was in a tube furnace microreactor connected to a mass spectrometer (MS) for online gas analysis.

Dynamic testing involved alternating between a flow of 50 mL of CO (10% in He), and 50 mL of O_2_ (5% in He), each flowed for 300 s, and calculating the quantity of CO which reacted with lattice oxygen to form CO_2_ from the MS response. The gas concentration ratios of 2:1 CO/O_2_ account for the fact that 2 mol CO react with 1 mol O_2_. Testing was performed each 100 °C from 300 to 700 °C, ramping at 20 °C min between tests, using 50 mg of the sample. During breakthrough testing, the sample was oxidized in O_2_ (5% in He) until MS responses were stable. The gas was then switched to CO (10% in He), at which point CO_2_ was observed. The time between the flow of CO and the appearance of CO in the MS was the breakthrough period, and from this, the quantity of lattice oxygen which was removed to form CO_2_ could be calculated. The sample was then reoxidized, and the process repeated at a higher temperature. See examples of the MS response of both methods in Supporting Information Section S6, along with reproducibility data.

### 2.9 Activity Testing

Catalyst activity testing was performed at the Emission Control Research Laboratories at Johnson Matthey, Sonning Common. 200 mg of the sample was mixed with 200 mg of cordierite (both 250–350 μm) and loaded into a fixed bed reactor with online gas analysis by FTIR. A gas mixture proportioned to model that of a typical exhaust feed in a gasoline vehicle was used, with a perturbed 3 s rich-lean environment created by alternating the concentration of CO + H_2_ and O_2_ (Supporting Information Section S7). The lambda variation was 0.97–1.02, averaging 0.99 (slightly reduced). The sample was heated from 110 to 500 °C at 10 °C min^–1^, then cooled at the same rate, and this program was repeated. These two parts of the experiment are differentiated by “run 1” and “run 2”. Run 2 is considered more representative of a working TWC catalyst, the catalyst having experienced exhaust gases. Conversions were calculated from the loss of reactant, with inlet concentrations recorded for each reaction through a bypass. Selectivities of NO to NH_3_, N_2_O, NO_2_, and N_2_ were calculated from outlet concentrations when the total NO conversion was >5% (see Supporting Information Section S8).

## 3 Results and Discussion

### 3.1 Structural Characterization and Aging of the Bare Ceria Zirconias

CZ1 and CZ2 were confirmed to be solid solutions with substitutional exchange of Ce by Zr by both PXRD and EXAFS analysis at the Ce K edge. The K edge is typically less easily accessible than the typical L_3_ edge but allows observation of second shell interactions (Figure 1a).^35,36^ The data below show splitting of the Ce–Ce peak, indicative of a new Ce–Zr scattering path, at a smaller distance consistent with the smaller ionic size of Zr^4+^. Two further formulations with larger differences in the Ce/Zr ratio (3:1 and 1:5) were also measured to highlight the difference in distances depending on the likely second shell neighbor of the absorbing Ce atom. For the various formulations measured, the Ce–Ce distance changed linearly with Zr concentration due to lattice contraction, in accordance with Vegard’s Law, confirming the presence of a true solid solution (Supporting Information Section S9).^37^ Ce L_3_ edge XANES was also collected to confirm the +4 oxidation state of Ce in fresh CZ1 and CZ2, also found in Supporting Information Section S9 along with further spectra from the Ce K edge.

**Figure 1:**
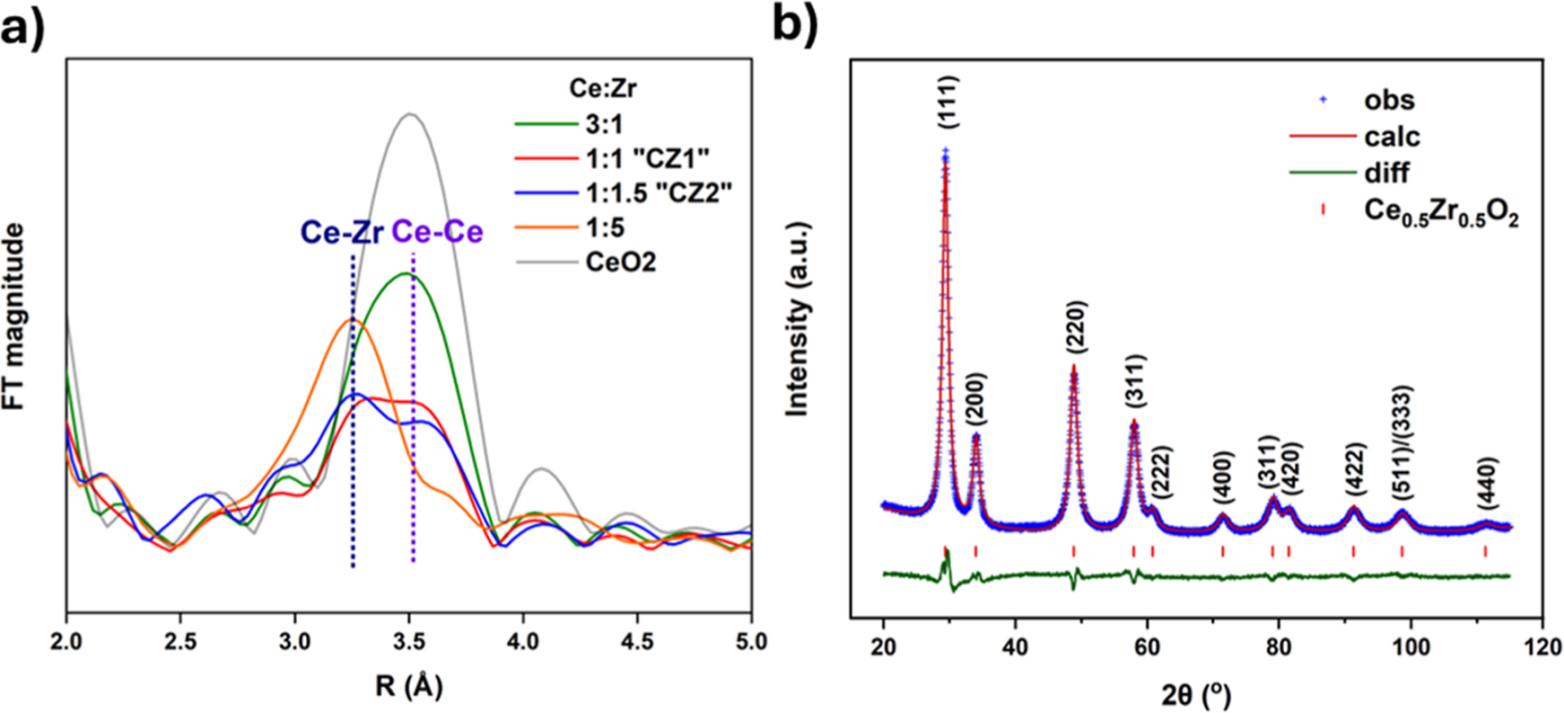
(a) *k*_2_ weighted Fourier transform of various 5% Pd/Ce_*x*_Zr_*y*_RE_*z*_O with different Ce/Zr ratios at the Ce K edge, showing the second shell Ce–Ce and the suggested Ce–Zr interaction. (b) Room-temperature profile fit from Rietveld refinement of the structure of CZ1 against PXRD data. Reflections are indexed to a fluorite *Fm* 3®*m* phase (*R*_w_ = 4.1%).

The fresh ceria zirconia catalysts here are described by a “pseudocubic” phase, and refined with *Fm* 3®*m* symmetry, in line with a previous PXRD focused structural study of ceria zirconia by Summer et al.^38^ This is not unanimously agreed in the literature and hence is discussed further in Supporting Information Section S10. Raman spectroscopy measurements supporting the assignment of a pseudocubic phase are also provided in Supporting Information Section S11. Figure 1b shows the profile fit for CZ1, following the Rietveld structure refinement.

The observed patterns of CZ1 and CZ2 are virtually identical, and full profile refinement details of both, along with the CZ2 powder pattern, can be found in Supporting Information Section S12. The refined average crystallite size of the fresh samples was 10 and 16 nm for CZ1 and CZ2, respectively. This is consistent with particle size analysis from TEM (Supporting Information Section S13), implying that each of the crystallites observed within the grains are entire diffracting domains. The lattice parameter of CZ1 is 5.28 Å, and for CZ2, it is 5.26 Å; CZ2 is smaller despite the large RE^3+^ dopants due to the lower Ce/Zr ratio.

The enhanced phase stability of CZ2 resulting from RE doping was confirmed by PXRD. A comparison of the two samples during lab in situ isothermal aging at 950 °C for 12 h is presented in Figure 2, and it shows the process of phase separation of CZ1, with none occurring for CZ2.

**Figure 2:**
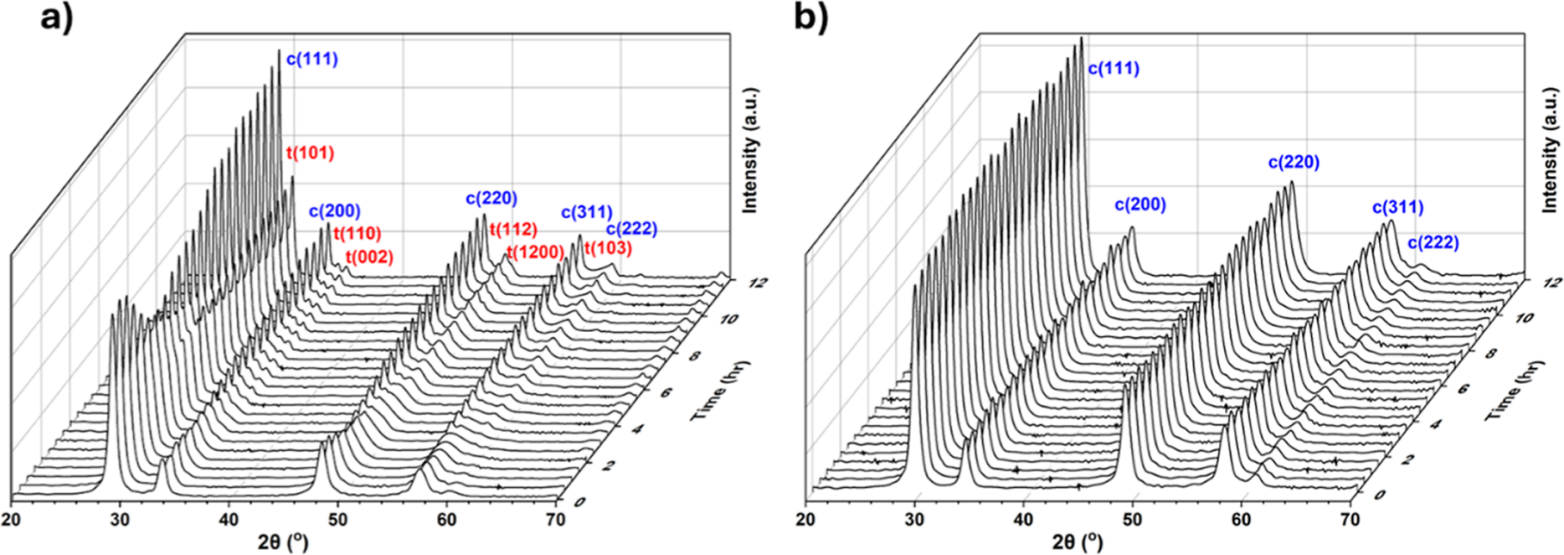
Stacked in situ PXRD patterns of (a) CZ1 and (b) CZ2 at 950 °C over 12 h in atmospheric air using an Anton Paar chamber. The data have been smoothed, and the reflections corresponding to the cubic “c” and tetragonal “t” phase are labeled.

Structure refinement of thermally separated CZ1 patterns gives a combination of a Ce-rich *Fm* 3®*m* phase and a Zr-rich *P*4_2_/*nmc* phase, in accordance with the previous literature.^12,13^ It was found that more severe aging conditions (1150 °C, 24 h) could eventually cause phase separation of CZ2 (Supporting Information Section S14), and PXRD from this aging treatment was refined, but performance testing was performed on the “milder” aged catalysts.

The enhanced stability of CZ2 was also reflected in the surface area and pore volume measurements (Supporting Information Section S15). Although the initial surface area of CZ1 was over twice that of CZ2, on aging at 950 °C for increasing lengths of time, the surface area and pore volume of CZ1 decreased rapidly, while CZ2 remained virtually unchanged.

### 3.2 Characterization and Accelerated Aging of Pd-Loaded Ceria Zirconias

The addition of 5% Pd resulted in little change to the fresh catalyst, although the surface area and pore volume decreased by approximately 25% after Pd impregnation (Supporting Information Section S15). Elemental mapping by EDS showed that the PdO nanoparticles (NPs) were not evenly distributed over the ceria zirconia particle, with large areas of higher Pd concentration, though there appeared to be a reasonable background dispersion (Figure 3, and Supporting Information Section S16). This is potentially a result of the incipient wetness process, though with a high loading of 5 wt % limited to occupying only the CZ crystallite surface, the maximum potential dispersion is low.

**Figure 3:**
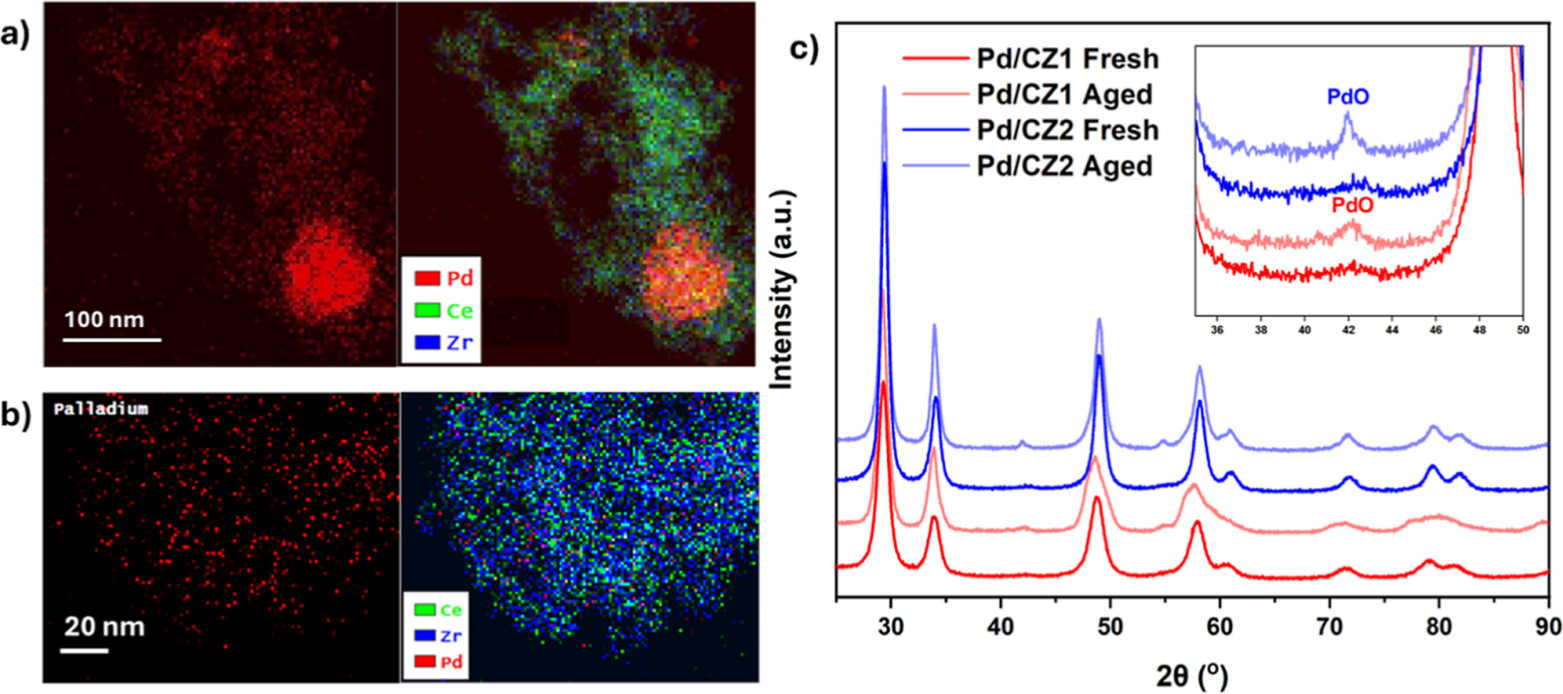
EDX analysis performed alongside HAADF-STEM imaging of fresh (a) Pd/CZ1 and (b) Pd/CZ2 showing the locations of Ce, Zr, and Pd. (c) Stacked room-temperature PXRD patterns of Pd/CZ1 (red) and Pd/CZ2 (blue), fresh (bold), and aged at 950 °C for 12 h (faded). An inset highlights one of the regions in which PdO reflections are observed, at 2θ = 42°. A Pd^0^ reflection would be observed at 2θ = 39° if present.

However, no PdO or Pd^0^ reflections were observed in the PXRD pattern (Figure 3c), indicating a reasonable dispersion with a mean size diameter of <5 nm,^39^ and *ex situ* XANES confirmed the Pd species to be 100% PdO on both CZ1 and CZ2 with no Pd–Pd metal distances observed in the EXAFS of the fresh catalysts (Supporting Information Section S17). Therefore, despite the apparent high levels of aggregation, these concentrated regions are assumed to consist of many small, amorphous PdO NPs. After the catalysts were aged at 950 °C, small reflections of PdO were observed in the PXRD patterns in both catalysts, indicating an increase in crystallite size and decrease in dispersion (Figure 5). For Pd/CZ1, 5% Pd^0^ was also calculated in the XANES by linear combination fitting (LCF), compared to 0% for the fresh catalysts, and a small scattering contribution from a Pd.1 path in Pd^0^, coordination number (CN) of 1, was observed in the EXAFS (Supporting Information Section S17). An increase in the PdO particle size was also confirmed by an increase in the CN of the Pd.1 and Pd.2 paths of PdO. Pd/CZ2, by comparison, remained 100% PdO by LCF. The Pd.1 and Pd.2 CN did increase more so than for Pd/CZ1, though no Pd^0^ paths were required for fitting. Therefore, additional growth of the PdO NPs occurred in preference to Pd^0^ formation.

Along with the PdO crystallite growth, TEM showed macroscale aggregation of Pd NPs on both CZ1 and CZ2. PXRD and bright-field imagine confirm that these continued to reflect regions of high concentrations of small Pd/PdO_*x*_ NPs rather than large coherent crystallites (Supporting Information S16).

### 3.3 In Situ Temperature Cycling by PXRD

PdO thermally reduces at temperatures above 700 °C, depending on the support, pressure, and atmosphere, and can reoxidize at temperatures below this. This is been observed by in situ XAFS by Keating et al.^40^ and by Cho and Kang^41^ for Pd on alumina-based supports. This PdO – Pd^0^ transition was chosen as a catalyst property by which to probe the strength of the metal–support interaction for Pd/CZ1 and Pd/CZ2. This correlation between the reoxidation and redispersion of Pd on a ceria zirconia support, and the strength of the metal–support interaction, is discussed and justified further in Supporting Information Section S18. Temperature cycling above and below this autoreduction temperature caused the reduction and subsequent reoxidation of PdO, and PXRD showed different efficiencies of Pd^0^ reoxidation between the two supports. Maximum temperatures of 850, 950, and 1050 °C were chosen, with the lower temperature always 650 °C, and a dwell time of 2h per cycle. The 950 °C cycle data are presented in Figure 4 along with calculated crystallite sizes. The 850 and 1050 °C data can be found in Supporting Information Section S19.

**Figure 4:**
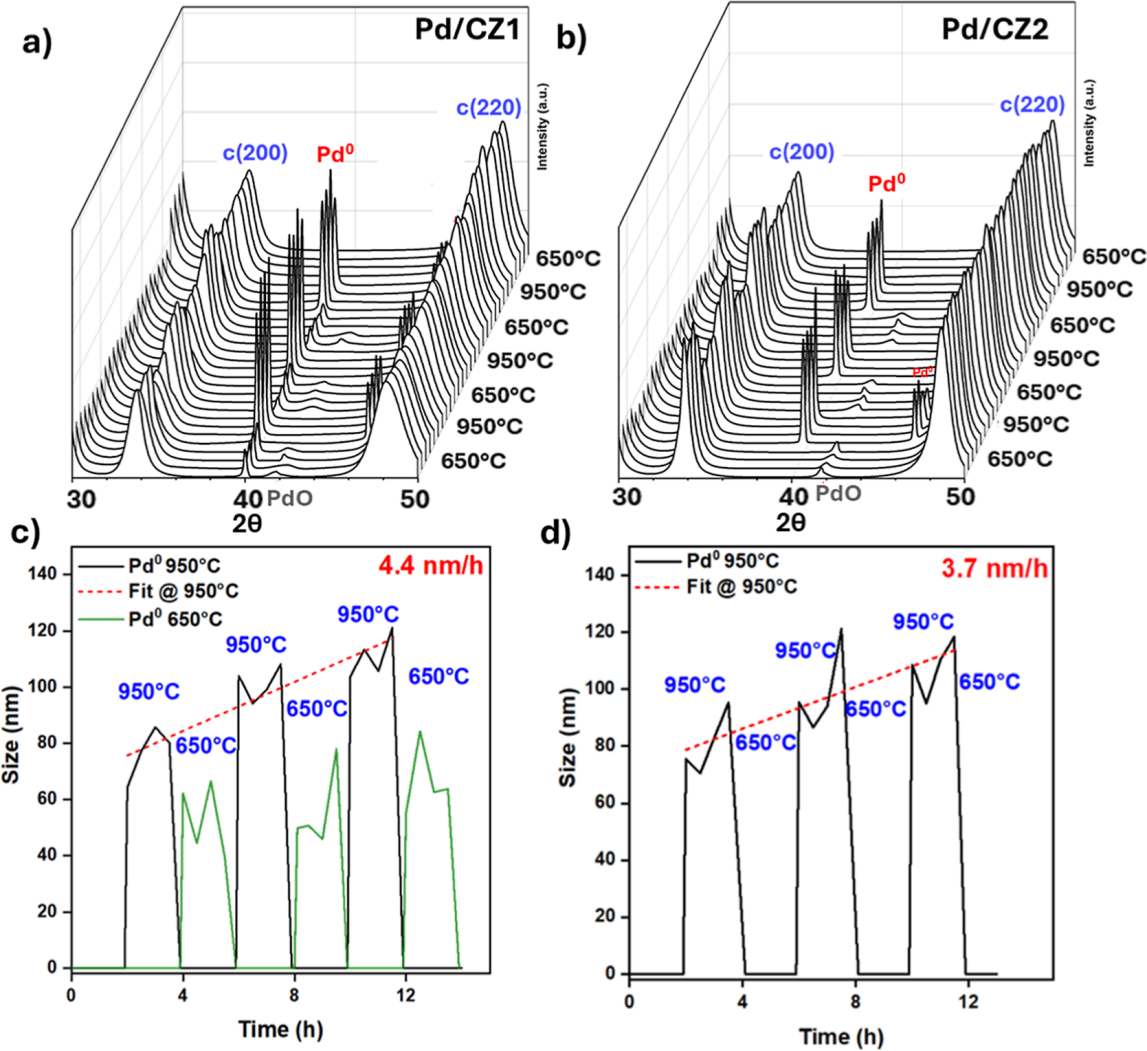
In situ PXRD patterns of (a) Pd/CZ1 and (b) Pd/CZ2, temperature cycled in atmospheric air using an Anton Paar chamber. A Savitzky–Golay filter was applied to the data for the sake of clarity. The calculated crystallite size of the Pd^0^ during the cycling is also shown for (c) Pd/CZ1 and (d) Pd/CZ2, performed on the raw data. Cycling temperatures were 950–650 °C. The reflection at 2θ = 40° is the (111) of Pd^0^ with *Fm* 3®*m* symmetry. At 2θ = 42° is the (110) of PdO with *P*4_2_/*nmc* symmetry, which overlaps with the CZ2 (200) and hence its alternating intensity. Phase separation of CZ1 is observed at high temperature.

Sharp Pd^0^ reflections were present at higher temperatures during cycling and either partially or entirely disappeared during the cooling cycles, accompanied by the emergence of small broad PdO reflections. This transition presumably accompanies the loss/partial loss of large crystalline metallic NPs to form small well-dispersed oxidic ones, though it is not possible to determine the quantity of amorphous Pd^0^ remaining by PXRD. Comparing the two samples during the 950–650 °C cycles, residual Pd^0^ reflections remained during the cooling cycle for Pd/CZ1 but not Pd/CZ2, an effect also observed but to a greater extent on cycling 1050–650 °C. This indicates the reoxidation process is more efficient on CZ2. Cooling from the lower temperature of 850 °C, apparent complete reoxidation occurred on both CZ1 and CZ2, confirming the similar metal–support interactions under less demanding conditions.

The size of the Pd^0^ at 950 °C, and also at 650 °C for Pd/CZ1, were calculated by structural refinement, though the PdO could not be calculated in this way given the size and shape of the reflections. Under all conditions, the size growth of the Pd^0^ NPs was continuous, despite the intermittent cooling and reoxidation to PdO. This is true also cycling between 850 and 650 °C and 1050–650 °C, regardless of whether the Pd^0^ forms a crystalline PdO phase during the cooling cycles. This indicates that the metallic NPs which form during one cycle are related to those formed during the subsequent cycle, demonstrating a “memory effect” of the Pd^0^ which affects the redispersion process. It is also noted that such continuous growth rates are more typical of an Ostwald Ripening sintering process, common also for larger NPs, as opposed to particle migration and coalescence, which is more commonly observed at the initial stages of sintering when NPs are small.^42,43^ Any remaining, amorphous Pd^0^ during the low-temperature cycles would act as a nucleation site upon autoreduction of the PdO at high temperatures. The in situ clustering of nanoparticles which redisperse when cooling is also commonly observed for Pt catalysts, unless the nanoclusters are stabilized during the high-temperature treatment as demonstrated by Liu et al.^44^

The reoxidation of Pd^0^ here is presumed to occur via a redispersion process, described in previous literature for the reoxidation of Pd^0^ NPs in air by Wan et al.^45^ These reoxidized NPs are presumed to remain in localized clusters, close to the supposed Pd^0^ nucleation sites, allowing the immediate resumption of sintering on reduction. An alternative reoxidation process, proposed by Xiong et al. for Pd/La–Al_2_O_3_, is one where oxidation occurs to areas within large metallic NPs, resulting in domains of both Pd^0^ and PdO within large polycrystalline particles.^46^ While individual large NPs that reversibly grow domains of PdO would be consistent with the nucleation sites indicated by the sintering process, they do not account for the significant influence of the support. The differences in Pd behavior on CZ1 compared to CZ2 throughout all temperature cycling work reported in this study indicate a redispersion mechanism as by this method the NP–surface contact would be much greater, accounting for different strengths of metal–support interactions.

### 3.4 In Situ XANES Experiment

PXRD only shows species that are crystalline and have sufficiently large, ordered domains. To determine whether the entire Pd^0^ phase is being oxidized during the cooling cycles, where Pd^0^ reflections partially or completely disappear, or only becoming amorphous, an in situ XANES experiment was performed (Figure 5 and Supporting Information Section S20).

**Figure 5:**
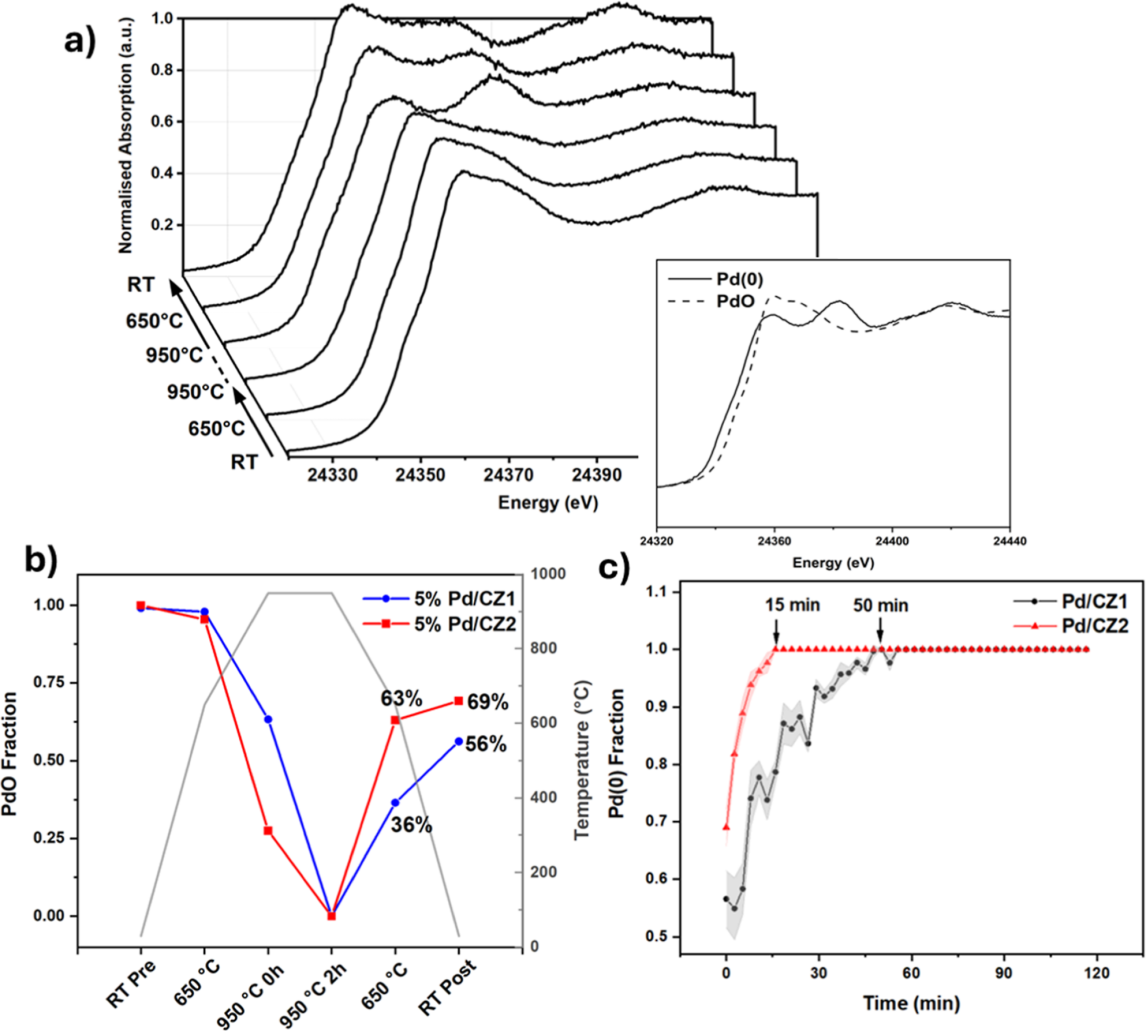
Results of an in situ XANES experiment performed using a capillary furnace on a B18 at DLS. (a) XANES spectra of Pd/CZ1 from isothermal points in the experiment, including the start and end of a 2 h isothermal dwell at 950 °C, with a reference spectra insert. (b) PdO fraction from LCF fitting at isothermal points during the reaction. (c) Pd(0) fraction as a function of time during the 950 °C dwell. LCF fitting error is represented by shaded regions in (c), though was too small to represent in (b), being < 1% for each fit.

The data show that, first, PdO reduction occurred more rapidly on CZ2 than on CZ1 on reaching 950 °C. TGA experiments following identical thermal profiles confirmed the reduction onset temperature for Pd on each support to be within 10 °C (Supporting Information Section S21), hence the difference is in the rate of reduction rather than time since initial reduction began. Similarly, the reoxidation of Pd^0^ back to PdO also occurred more rapidly on CZ2; most of the oxidation process (63%) had finished by the 650 °C isothermal measurement during cooling, unlike on CZ1 where only 36% had reoxidized. This was mirrored by the TGA experiment, where an initial fast reoxidation of approximately half of the Pd^0^ was followed by a more gradual process for the remaining part on CZ1, unlike the rapid and almost total reoxidation that occurred on CZ2. Finally, the total amount of Pd reoxidation was greater for Pd/CZ2, with the fully cooled sample containing 13% more PdO than Pd/CZ1 by LCF of the XANES. This is consistent with the above observations by PXRD temperature cycling—that Pd^0^ reoxidation is more complete and occurs within a narrower temperature range, on CZ2. It is worth noting that EXAFS analysis of fresh and aged samples showed no evidence of the Pd–Ce interaction or indicated any encapsulation during the reoxidation process (Supporting Information Section S17).

From the PXRD cycling data above (950–650 °C, Figure 4), small Pd^0^ reflections remained during the 650 °C cooling cycle for Pd/CZ1 but not Pd/CZ2. XANES however shows the presence of Pd^0^ on both supports at this temperature, albeit with a smaller % on CZ2. While the Pd^0^ NPs may be smaller and more dispersed on CZ2 during cooling, hence not producing reflections by PXRD, both materials do contain Pd^0^ which could act as the previously discussed nucleation sites for Ostwald ripening.

Another in situ experiment was performed to complement the in situ PXRD and XAFS results above by combining both techniques. This allowed the reduction and reoxidation temperatures obtained by both methods to be accurately compared without potential error occurring from different environment cells. This experiment could not however replace the two separate experiments due to the slower time resolution of the XANES. The results confirmed that the reduction and reoxidation temperatures by both techniques were equivalent, with the chemical and structural changes occurring simultaneously. The results also confirmed again that the reoxidation of Pd^0^ is slower on CZ1, occurring gradually for much more of the cooling period than reoxidation on CZ2 (PXRD data in Figure 6, with XANES in Supporting Information Section S22). At 650 °C, the LCF of the XANES calculates the Pd NPs as 94% metallic on CZ1, but only 55% metallic on CZ2, in line with the above in situ XANES experiment but with some expected discrepancy due to the use of different cells. Interestingly, PdO reflections were observed in the PXRD of Pd/CZ2 immediately upon the reoxidation of Pd^0^, but none were observed for Pd/CZ1, where the Pd^0^ was slow to reoxidise and eventually become amorphous PdO_*x*_.

**Figure 6:**
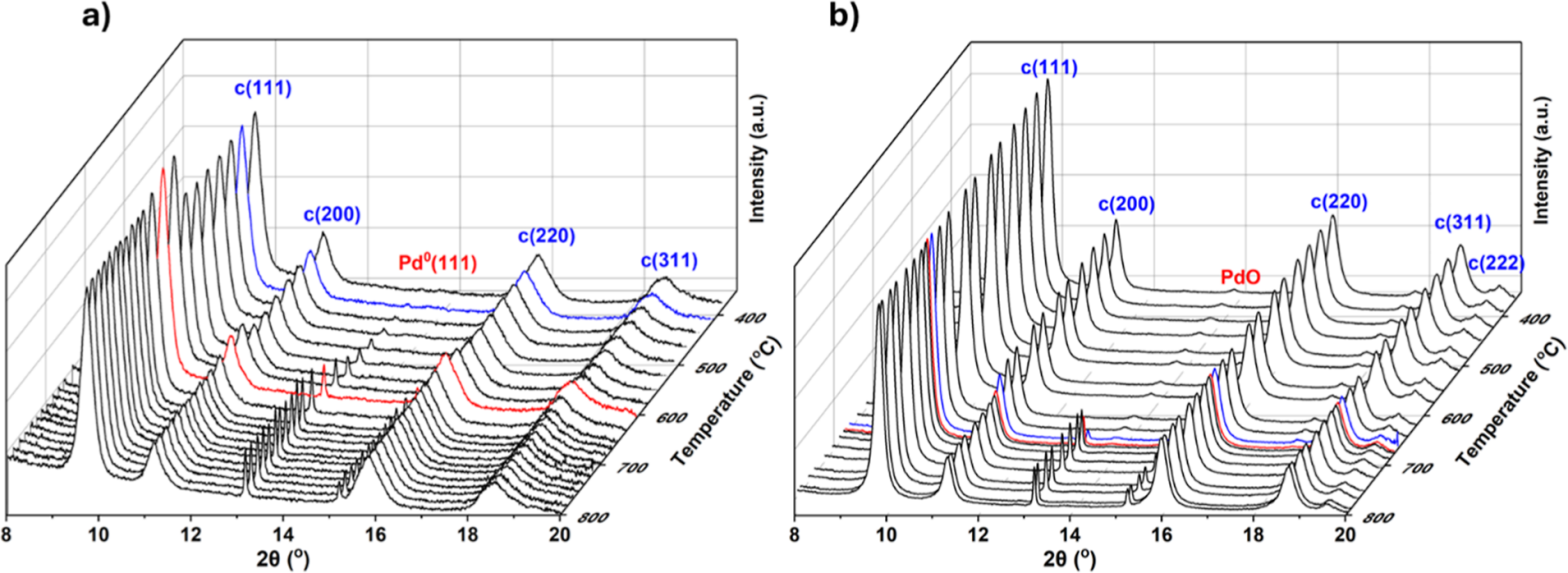
A portion of the XRD patterns collected in situ in air on XMaS at 24 keV (0.496 Å) during cooling of (a) Pd/CZ1 and (b) Pd/CZ2 from 900 °C. Pd^0^ reoxidation onset and end are indicated in red and blue, respectively.

Together these various in situ experiments demonstrate a higher NP mobility of Pd on CZ2 as it has been justified by the influence of the support that both the reduction and the reoxidation process require particle migration. There is a strong correlation between the strength of metal–support interaction and NP mobility.^47,48^ Hence, weaker Pd/CZ interaction is suspected in Pd/CZ2 compared to Pd/CZ1, as evidenced in the later OSC and activity results.

### 3.5 Long Duration Aging

The ability to perform X-ray diffraction measurement in situ during long aging sequences, without the need for removing the sample from its aging environment, is unique to beamline I11 at DLS.^29^ Using this facility, we were able to compare the structural changes ceria zirconias undergoes during thermally accelerated aging, utilized in virtually every reported aging study of the materials, to those which occur under a milder and lengthier aging process.

Two accelerated thermal treatments (12 h at 950 °C and 24 h at 1150 °C) were compared with a 7 week treatment at 850 °C. We were able to collect PXRD patterns every seventh day at temperature during this treatment. A constant temperature of 850 °C was chosen as it is sufficiently low to not cause phase separation, as tested over a three-day laboratory aging, but high enough to ensure structural change would occur. However, more importantly it also represents a temperature TWCs can be anticipated to reach during acceleration or high-load events.^49,50^ A selection of the data and results is displayed in Figure 7. Full refinement details for the data shown below and additional results including changing phase fractions, and the plotted refinement results for Pd/CZ2, can be found in Supporting Information Section S23.

**Figure 7:**
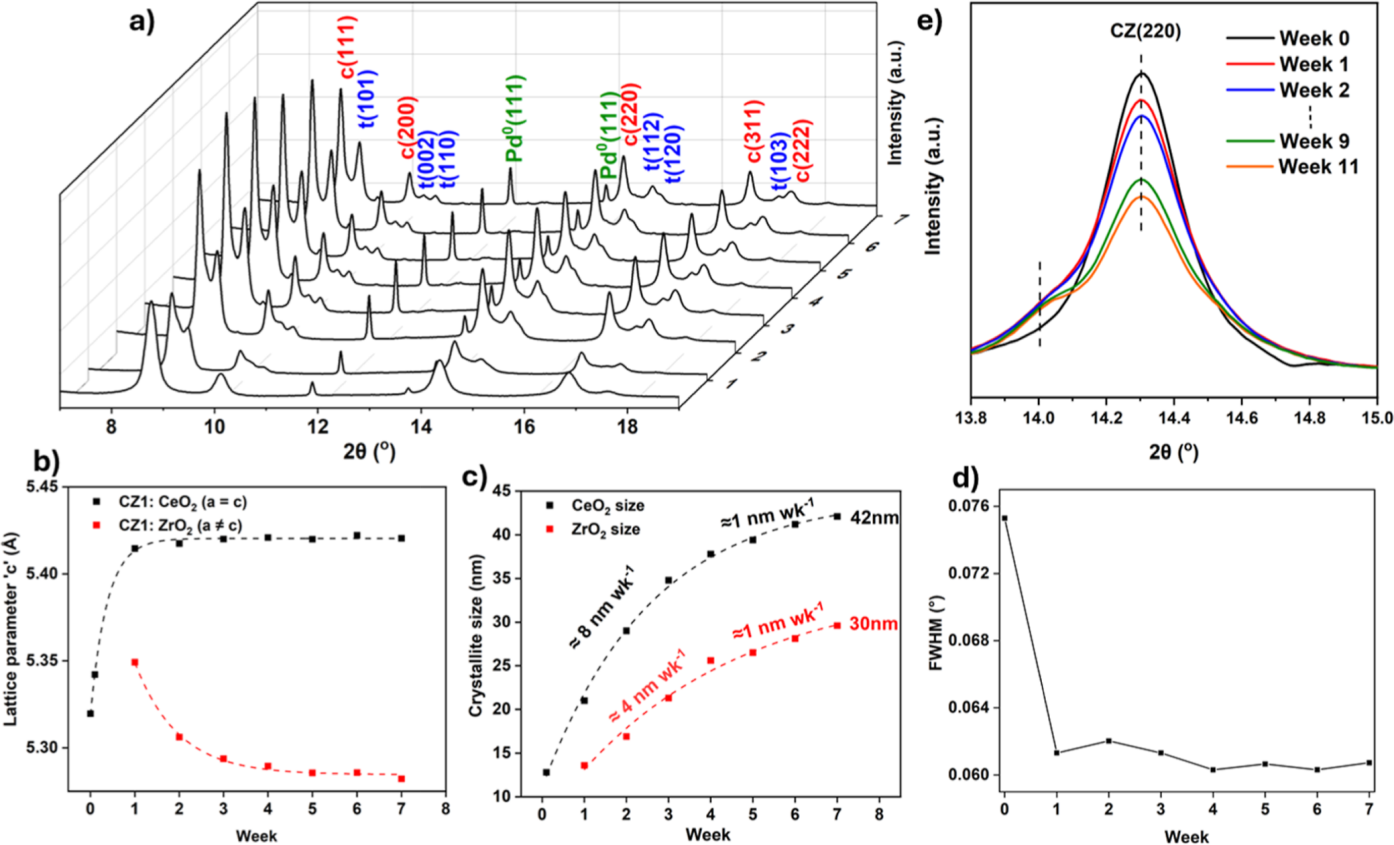
(a) Stacked in situ PXRD patterns of Pd/CZ1 collected at 850 °C over 7 weeks. The final weeks data have reflections labeled “c” and “t” referring to a cubic Fm3®*m* phase and tetragonal *P*4_2_/*nmc* phase of the ceria zirconia. The Pd^0^ reflections are also indexed. (b) The calculated lattice parameters and (c) calculated crystallite size, both refined from the measurements of Pd/CZ1. At week 0, only the cubic phase was fit, and from week 1 of the separated cubic CeO_2_ rich and tetragonal ZrO_2_-rich phases (only the “c” value for ZrO_2_ is shown). The lattice parameters at 6h come from a complementary laboratory based in situ experiment. (d) fwhm of the Pd(111) reflection on Pd/CZ1, (e) a selection of data of the (220) reflection of CZ2 at 850 °C over 11 weeks showing the development of a low shoulder peak from week 1.

The lattice parameter at week 0, the freshly heated sample, for both CZ1 and CZ2, was that of a homogeneous single phase ceria zirconia, the structure of which was described earlier. For CZ1, phase separation then occurs (Figure 7a), and after 1 week, the cubic phase had expanded dramatically and reflections of a tetragonal phase were present. Given the larger ionic size of Ce^4+^ than Zr^4+^, the cubic phase must be CeO_2_ rich and the tetragonal phase ZrO_2_ rich. The lattice parameters showed that the composition of the cubic phase was stable after 1 week (Figure 7b). Empirical methods were used to calculate stoichiometries of the cubic phase (Supporting Information Section S1), yielding the final composition of the cubic phase at week 7 of Ce_0.9_Zr_0.1_O_2_.

The lattice of the tetragonal phase, however, continued to shrink exponentially. This phase must initially have contained Ce cations, which were gradually removed from the lattice and replaced by Zr. A small proportion of Ce was expected to remain after the lattice size plateaued in order to stabilizing the tetragonal phase as pure ZrO_2_ would have a monoclinic structure at this temperature. Indeed the final lattice size of ZrO_2_ was slightly larger than that of pure tetragonal ZrO_2_. After being expelled from this phase, the Ce was incorporated into the cubic phase, evidenced by the continued evolution of total crystalline phase fractions which only stabilized once the ZrO_2_ lattice size had stabilized (see Supporting Information Figure SI22.2).

The sintering of the two phases was independent of when the composition of each phase stabilized; they began to plateau similarly, despite the cubic phase stabilizing much more quickly (Figure 7c). Extrapolating from the above exponential fits of crystallite size, the cubic and tetragonal phases would plateau at 45 and 34 nm, respectively. Therefore, at this temperature, sintering would never yield the 78 and 48 nm sizes of the respective phases reached under example accelerated aging temperatures of 1150 °C over 24 h (Supporting Information Section S14 previously). Crystallite size of the Pd^0^ was calculated only to maintain a good fit but the values not expected to be accurate, being above 100 nm and accounting for the limitations of the Scherrer equation.^51^ No decrease of the fwhm of the Pd^0^ reflections occurred after week, though this may be due to the dominating effects of instrumental broadening rather than a stabilizing of crystallite size (Figure 7d).

No obvious compositional changes or sintering of CZ2, the RE-stabilized ceria zirconia, was observed; however, the sample was rerun for a longer period due to the observation of a low angle shoulder which developed on each of the ceria zirconia reflections (Figure 7e). Given the larger ionic size of the RE^3+^ ions, and the propensity of these elements to become mobile during high-temperature calcinations in ceria materials, a Ce-stabilized rare earth oxide is proposed.^52,53^ It is further proposed that this phase was never fully integrated into the ceria zirconia solid solution as its removal did not cause any lattice contraction of the major cubic phase. It most likely corresponds to amorphous material, which became ordered given sufficient time at temperature. Notably, this phase was not observed during any accelerated aging studies, even ones sufficiently harsh to cause significant phase separation of the stabilized ceria zirconia.

The focus in this work was a time versus temperature study of the structural properties of ceria zirconia. In related work, a comparison of a laboratory-accelerated aged TWC with those aged in both a laboratory bench engine, and one extracted from a vehicle after 60,000 km of use, was performed by Guiliano et al.^28^ They observed that, on the whole, the structural degradation between the vehicle aged and accelerated aged samples were similar, resulting in only minor discrepancies in the levels of sintering and the loss of OSC. Light-off temperatures and conversion profiles were more affected, thought to mostly result from the range of poisons and inorganic component buildup which affects vehicle aged catalysts. The results from this long-duration study align with Guiliano’s findings, indicating that, overall, the structural changes induced by accelerated aging are sufficiently representative to provide essential thermal integrity information during the R&D process. However, the phase changes of Pd/CZ2 demonstrate how relying solely on accelerated aging experiments to infer the long-term aging behavior of materials may lead to inaccurate conclusions.

The limited sample quantity from these experiments made performance testing of the recovered samples impossible. Is it hoped that future work will allow both the long duration aging of TWC materials under more relevant conditions (gases, humidity) and that this can be performed in such a way as to recover sufficient sample for performance testing. The following activity results therefore refer to samples aged at the accelerated times and temperatures utilized in the earlier sections of this paper.

### 3.6 OSC Testing

The OSC of the bare ceria zirconias, tested using the dynamic OSC method, is reported in Table 1 below. The total available OSC is also compared with the fast OSC (that which is available in the first 3.5 s of CO exposure) in Supporting Information Section S24.

**Table 1:** Dynamic OSC Values of CZ1 and CZ2, Calculated from Total CO Oxidized over 300 s at Each Temperature^a^

temperature (°C)	OSC (μmol/g)		% Ce utilization	
	CZ1	CZ2	CZ1	CZ2
300	210	135	12	10
400	685	625	40	48
500	812	695	48	53
600	903	759	53	58
700	984	820	58	62

a The % utilization is calculated from the stoichiometries of each support.

While absolute OSC of CZ1 was higher than CZ2, normalizing to the stoichiometric amount of Ce showed that more of the available Ce can be reduced in CZ2. Surface area may play a large role, indicated by previous literature;^54^ however, data were also normalized to surface area and the results were similar, evidencing the large contribution of the chemical and structural differences in the supports over the contribution of surface area. The higher OSC of CZ2 is attributed predominantly to its lower Ce/Zr ratio, 1:2 compared to 1:1 for CZ1, for reasons now described.

Literature indicates that increasing the Zr content increases the reducibility of ceria, lowering its reduction enthalpy.^10^ Regarding the local environment of the Ce, the probability that the Ce being reduced is bonded to Zr, through O^2–^, increases as the Zr ratio increases. This coordination environment was found to be required for oxygen removal in earlier literature; during reduction oxygens are removed from the coordination sphere of the Zr, though only resulting in reduction of the Ce^4+^.^54,55^ RE doping may also affect OSC, but only the rate of oxygen removal and the surface reducibility, not the extent of bulk reduction as RE^3+^ ions are not redox active. Aging the supports decreased the OSC of CZ1 more than CZ2 due to surface area loss and phase separation, both processes which were inhibited for CZ2.

The breakthrough method was applied to the Pd-loaded samples. While a 5% Pd loading is higher than typically observed for such materials, Clark et al. demonstrated little difference in amount of reduction and reoxidation of Pd/CeO_2_ with a 1% versus 5% loading.^30^ Figure 8 describes the oxygen storage capacity, in terms of % Ce utilization of *all* reducible components (Ce plus Pd), of Pd/CZ1 and Pd/CZ2, freshly prepared and after thermal aging.

**Figure 8:**
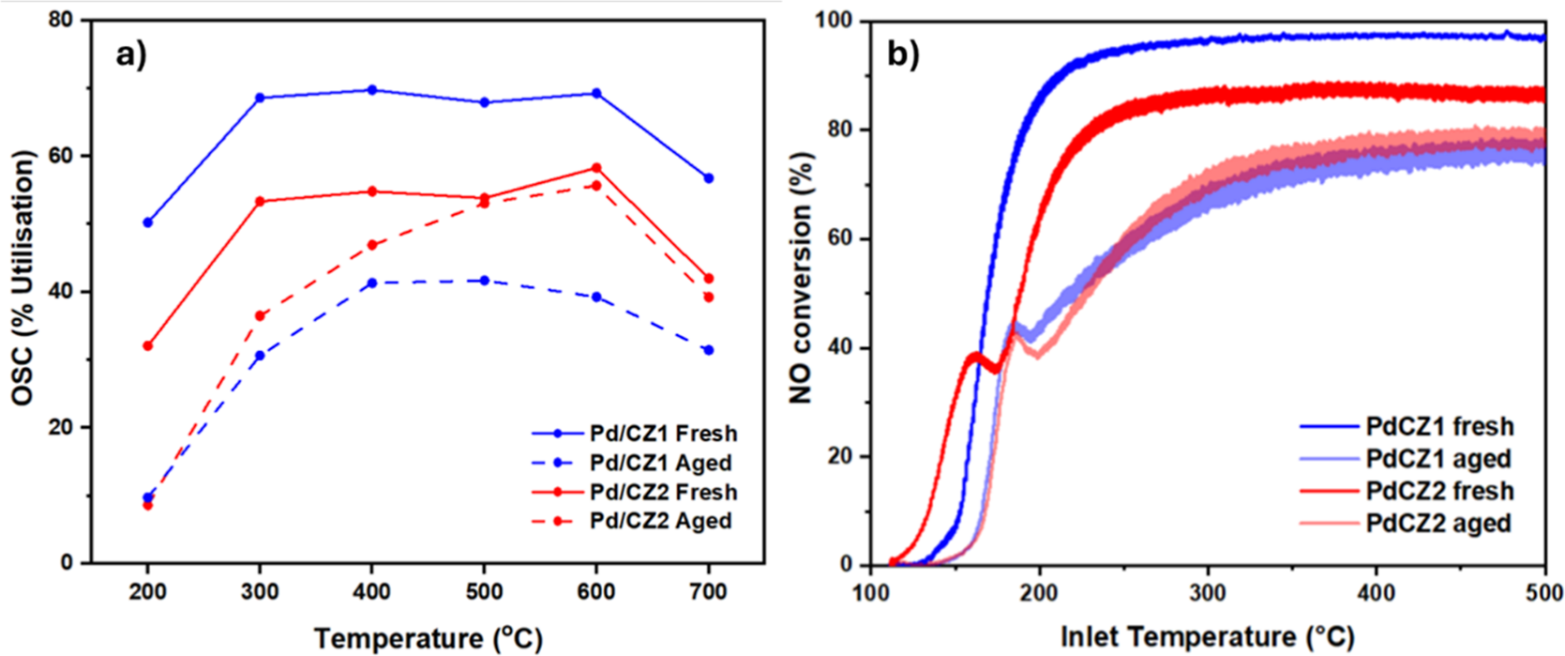
(a) Oxygen storage capacity in total % utilization of Pd/CZ1 and Pd/CZ2 (accounting for both Pd and Ce reduction), fresh and aged, using the breakthrough method in the temperature range 200–700 °C. (b) NO conversion of Pd/CZ1 and Pd/CZ2 from TWC activity testing in model exhaust gas with 5% water. Data are from the first temperature ramp (Fresh r1), and after thermal aging. Thermal aging for both experiments was in air at 950 °C for 12 h.

The OSC of both CZ1 and CZ2 improved greatly on addition of Pd, in agreement with the literature.^25^ The low-temperature OSC was particularly enhanced in this way. A backspillover mechanism at the Pd-ceria zirconia interface provides a lower energy pathway for the removal of the oxygens, which diffuse to the interface from the ceria zirconia lattice.^22,25^ The promotion of the Pd is not only to the CZ at the interface region; Nagasawa et al. used secondary ion mass spectrometry to demonstrate that CZ reducibility is improved in a large radius surrounding the Pd NPs and penetrating far into the bulk.^21^

While CZ2 slightly outperformed CZ1, the performance of fresh Pd/CZ1 was far superior to that of fresh Pd/CZ2. This provides further evidence of the stronger MSI of Pd/CZ1 indicated by in situ characterization as oxygen migration from the ceria zirconia to the Pd near the interface region is enhanced. Aging Pd/CZ1 decreased the OSC equally across all temperatures; however, aging Pd/CZ2 resulted in the loss of OSC only below 500 °C, with the high-temperature OSC unaffected. OSC at these higher temperatures is attributed mostly to the ceria zirconia, and hence, the enhanced stability of CZ2 is responsible for maintaining its OSC after aging. At low temperatures, OSC loss is attributed to severe Pd sintering, and this effect dominates any differences in the metal–support interaction between the samples, with slightly reduced sintering of the Pd on CZ2, as observed earlier in the temperature cycling PXRD study (Figure 5) presumably aiding the OSC of this sample at these lower temperatures.

### 3.7 Activity Testing

TWC activity testing was performed to calculate the conversion of CO, NO, and hydrocarbons and nitrogen selectivity. NO conversion is presented in Figure 8b, representing well the total activity differences between the samples, with other activity data in Supporting Information Section S25. Comparisons of the T_50_, temperature at 50% conversion, and the N_2_ selectivity are presented in Figure 9 below, with further conversion data given in Supporting Information Section S25. Additionally, in Supporting Information Section S26, an identical set of performance testing for the same catalysts but with Pt as the active metal rather than Pd is presented. These results reiterate that, while other PGM catalyst formulations may yield better performance when fresh, their resistivity to aging and overall lifetime is of significant importance when evaluating materials for TWC application.

**Figure 9:**
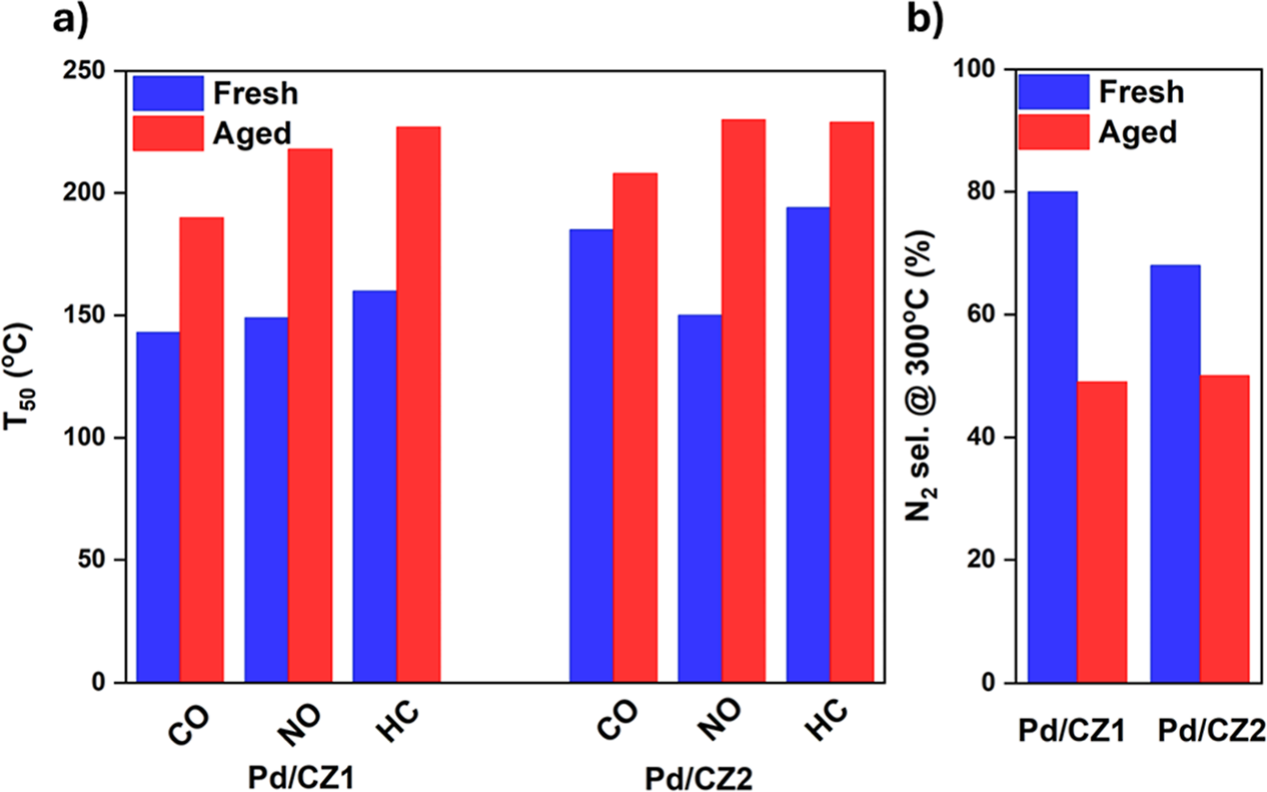
(a) *T*_50_ values (temperature at 50% conversion) for the conversion of CO, NO, and HC (total hydrocarbons), along with (b) the N_2_ selectivity at 300 °C for Pd/CZ1 and Pd/CZ2 fresh and aged (950 °C, 12 h, in air). Experiments were under model exhaust conditions.

The weaker MSI of Pd/CZ2 is evidenced in both the CO and NO light-off curves of the fresh catalysts where a lag in conversion at 160 °C occurred (see Figure 8a and Supporting Information Section S25). This feature was only observed for fresh Pd/CZ2, not Pd/CZ1, though on repeating the light-off test on the same catalyst, unremoved from the reactor, did not, this feature did not appear in the CO conversion and was much dampened in the NO conversion. It is likely caused by a requirement for activation of Pd/CZ2 (conditioning with temperature and reducing gases) to enable the typical involvement of ceria zirconia oxygen by a Mars van Krevelen route, present already for Pd/CZ1 due to its stronger MSI. The higher OSC of Pd/CZ1 demonstrated earlier also resulted in improved N_2_ selectivity, 13% higher than that of Pd/CZ2 at 200 °C, specifically reducing the quantity of NH_3_ formed. The oscillations in conversion and selectivity are also less significant for Pd/CZ1, again a result of its improved fast OSC compared with Pd/CZ2, as the ceria zirconia is better able to respond to the 3s gas composition fluctuations.

After aging, the CO and NO T_50_ and total conversion of both Pd/CZ1 and Pd/CZ2 were similar, as was their N_2_ selectivity. However, aging was more detrimental to Pd/CZ1, which initially performed superior to Pd/CZ2. The significantly reduced OSC of aged Pd/CZ1 is observed in the thicker lines in both the light-off curves and selectivity plot (see Figure 8a and Supporting Information Section S25), again a result of its lower ability to respond to fluctuating gas composition. The change is much less for Pd/CZ2, which showed equally thick lines in the fresh catalyst. As with the low-temperature OSC results, Pd sintering seems to dominate the catalytic properties, more so than the differences in the metal–support interaction and the phase separation of CZ1. Furthermore, after aging, both catalysts required an activation process to access their OSC, with both showing the light-off feature initially present in only Pd/CZ2, representing a lag in conversion at 190 °C for NO and CO conversion.

## 4 Conclusion

### 4.1 Pd Structure and Pd–Ce Interaction

The relationship between the strength of the metal–support interaction and surface NP mobility is well-known; generally, the stronger the interaction, the more anchored the NPs. The above results have demonstrated a difference in MSI between Pd and ceria zirconia depending on the stoichiometry of the support and the presence of dopants, evidenced by characterization methods and reflected in the catalyst OSC and TWC performance. The weaker MSI of Pd/CZ2 is proposed to be due to a more stable ceria zirconia surface, the result of its lower surface area, lower Ce content, and the presence of dopants. This lower MSI improves Pd NP mobility, evidenced by the faster autoreduction of PdO on CZ2, and the faster and more complete subsequent Pd^0^ reoxidation, which is presumed to occur through a redispersion process. However, this weaker MSI is detrimental to the catalyst performance for reactions that rely on oxygen mobility between the support and the PGM. Therefore, fresh Pd/CZ2 is outperformed by Pd/CZ1, which has a stronger MSI and hence improved oxygen transfer at the Pd–Ce interface.

### 4.2 Catalyst Aging and RE Doping

RE doping and lower Ce/Zr ratios are implemented to improve catalyst stability. However, after *ex situ* aging, during which severe phase separation and surface area loss have occurred to the undoped CZ1 support but not to doped CZ2, the light-off profiles and general performance of the two catalysts are equal. This may suggest that RE doping does not provide any performance advantages; however, the thermal aging performed for the purpose of this study was (a) in air and (b) accelerated. It is likely that subsequent aging treatment and more representative aging conditions (e.g., transient gases, hydrothermal, lower temperatures for longer times) would lead to greater differences between the supports, and under such conditions, Pd/CZ1 would likely continue to deactivate at a faster rate than Pd/CZ2. Indeed, the long duration PXRD experiment confirmed that RE doping really can inhibit phase separation at times and temperatures more similar to those demanded of a TWC, though unexpected minor phases may form domains breaking up the ceria zirconia structure.

By in situ aging, Pd/CZ2 showed an advantage over Pd/CZ1. The transient studies including temperature cycling (Figure 5) demonstrate that the Pd NPs are more recoverable in situ on CZ2, repeatedly reoxidizing completely to PdO. Pd/CZ1 by comparison continued to contain large metallic Pd^0^ fractions during cooling cycles, implying more total Pd agglomeration and therefore a decrease in the quantity of the Pd–Ce interface. This interface is essential for OSC. Therefore, while catalysts which have been aged *ex situ* and tested show little difference in performance, under transient conditions in a TWC, CZ2 may provide advantage by helping to prevent irreversible Pd agglomeration following periods of high temperature or stretches of time under reducing atmospheres.

## Supplementary Material



## Notes

Additional methodology information, including gas compositions for activity testing, powder diffraction refinement information, and comparisons of dynamic and breakthrough oxygen storage testing, experimental data complementing that in the main manuscript, and comparisons of Pd and Pt (PDF)

## Abbreviations

TWC: three-way catalyst
CZ: ceria zirconia
OSC: oxygen storage capacity
PGM: platinum group metal
