# Supplementary material for: Inhibition of Metal–Support Interactions by Rare-Earth Doping in Palladium/Ceria Zirconia Three-Way Catalysts
Source: Chem Mater. 2025 Aug 29;37(18):7214–26. doi: 10.1021/acs.chemmater.5c01417 (PMC12461787; doi:10.1021/acs.chemmater.5c01417)
Supplement: Supplementary file 1 [file cm5c01417_si_001.pdf]

Supporting information: The inhibition of metal-support interaction by rare earth doping in palladium/ceria zirconia three-way catalysts

Lucy Costley-Wood[1,2], Nicolás A. Flores-González[1,3], Claire Wilson[1], Paul Thompson[4], Sarah Day[5], Veronica Celorrio[5], Donato Decarolis[5], Ruby Morris[1], Manfred E. Schuster[6], Huw Marchbank[6], Timothy I. Hyde[1,6], Amy Kolpin[6], Dave Thompsett[6], Emma K. Gibson[1]**

*Email: L.Costley-Wood@UCL.ac.uk

*Email: Emma.Gibson@Glasgow.ac.uk

[1]University of Glasgow, Glasgow, G12 8QQ, United Kingdom

[2]University College London, London, WC1H 0AJ, United Kingdom

[3]Faculty of Engineering, University of Concepción, Concepción, 4070386, Chile

[4]ESRF – The European Synchrotron, Grenoble, 38000, France

[5]Diamond Light Source, Didcot, OX11 0DE, United Kingdom

[6]Johnson Matthey Technology Centre, Reading, RG4 9NH, United Kingdom

SI 1 Refinement details of CZ samples from laboratory PXRD.

Structure analysis was carried out by the Rietveld method,[1] employing GSAS-II software.[2] The R_w fit indicator, and esds value, were used to assess the quality of the refined structural model.[3] Refined parameters typically included the background (using the Chebyshev function, typically with 5 coefficients), unit cell parameters, crystallite size, phase fraction and in some cases fractional atomic coordinates. When refining U_{iso} values for ceria zirconias, when only a single phase was observed, a constraint equation was used to add equivalence for all cations, as they are similar elements which occupy the same site in the unit cell.[4] This was not applied to ceria zirconias which had undergone significant phase separation. Atomic site occupancies were fixed to the intended stoichiometry of the synthesised samples. Calibrations using 3 different $Ce_xZr_{1-x}O_2$ samples, where x = 0.5 – 1, confirmed the precision of the synthesis method, hence confirmed this method was appropriate. This method of fixing occupancies during refinement for ceria zirconias was also deemed appropriate by Summer et al. employing powder neutron diffraction (PND).[4] Figure SI1 shows the linear correlation between the Ce fraction, the shifting position of the (111) reflection, and the calculated lattice parameter. The position of the (111) reflection was taken directly from the raw data so is not subject to any processing or analysis, removing any risk of overfitting, but lattice parameter was refined. Refinements were run until data and fit converged, and the amount of shifting in the final cycle approached zero.

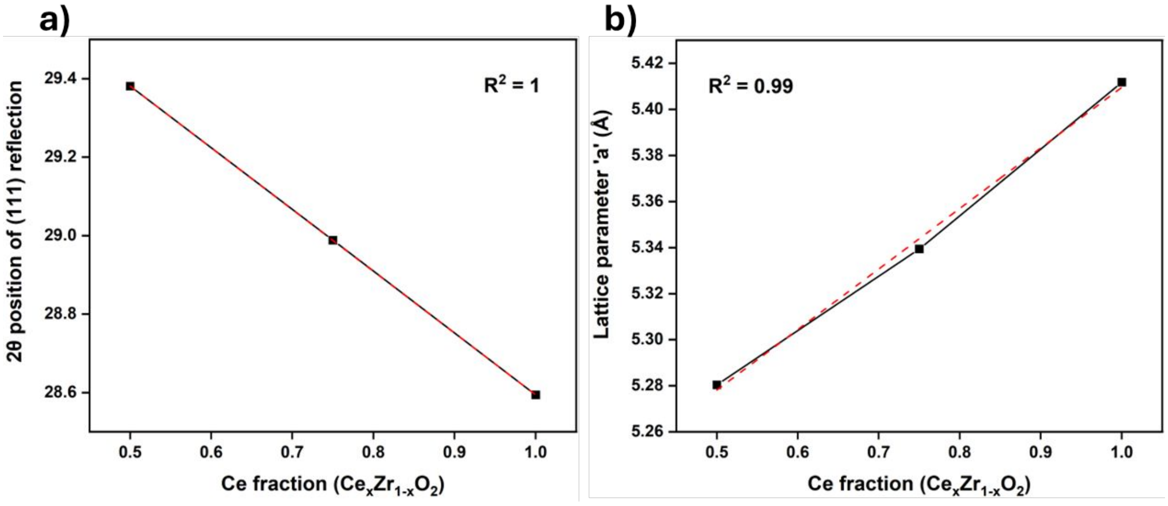

Figure SI1. **a)** 2θ position of the (111) reflection of the Fm$\overline{3}$m phase at 2θ = 29 °, and **b)** lattice parameter, as a function of Ce fraction in $Ce_xZr_{1-x}O_2$. Note for this crystal structure, lattice parameter a = b = c. Refined crystallite sizes were 10-11nm for all samples.

Oxygen occupancy in CZ1 should be 1. However, for CZ2, doping the system with trivalent RE cations introduces oxygen vacancies, and this was accounted for by reducing the occupancy of the oxygen in CZ2 by an amount calculated to maintain charge neutrality, and fixing it. It worth noting that lab-based PXRD is not an accurate method to determine site occupancy factors specifically if heavy elements are present. PND could be a better alternative, however it also needs to be treated with caution as it can overestimate the number of vacancies.[5]

SI2 Long Duration Experiment

Pd/CZ1 and Pd/CZ2 were pelletised using a 5 mm dye to ≈ 0.3 mm thickness, and each loaded into a vertically mounted Linkam TS1500 cell (Figure SI2.1). The cell uses ambient heating and water cooling, able to achieve a maximum temperature of 1500 °C at ramp rates of 200 °C min^{-1}. It has gas inlet valves, but these were left open to ambient air during the experiment. Two windows, with a combined thickness of 1.5 mm, allow the beam to pass through the sample. The pellet is held in place by a small metal ring at the front, and a ceramic cup at the back. The thermocouple sits to the side of the pellet, and its position is fixed.

Integrated software allows the heating to be controlled from the beamline control room. The two cells were run simultaneously, measured one after the other.

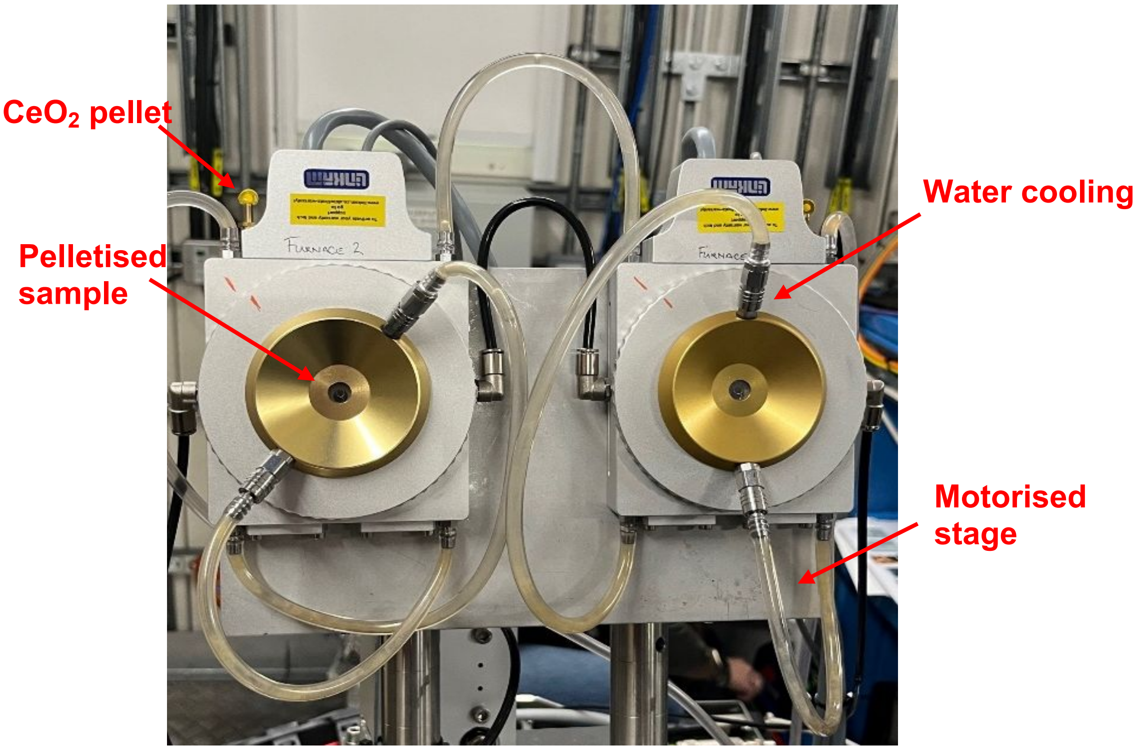

Figure SI2.1. Photo of the two TS1500 Linkam cells containing pelletised Pd/CZ1 and Pd/CZ2 on the motorised sample stage in EH2 on I11. The cells use ambient heating and water cooling.

Data calibration:

First, a LaB6 standard was pelletised and loaded into each Furnace. A measurement was taken at 25 keV and used to create instrument profiles for each Furnace (Figure SI2.2a). This was required as the backgrounds for each furnace were different, as one had a diamond window and one a quartz. Once the experiment had begun, ongoing data correction used a CeO_2 standard obtained from the National Institute of Standards and Technology (NIST), which was pelletised and attached to the top of each furnace by the beamline scientists. Each week correction to the wavelength and sample-detector distance generated from the CeO_2 data was applied automatically to the sample data (Figure SI2.2b,c). A displacement correction was also

applied to account for the difference in distance from the beam between the CeO_2 pellet and the sample pellet inside the cell.

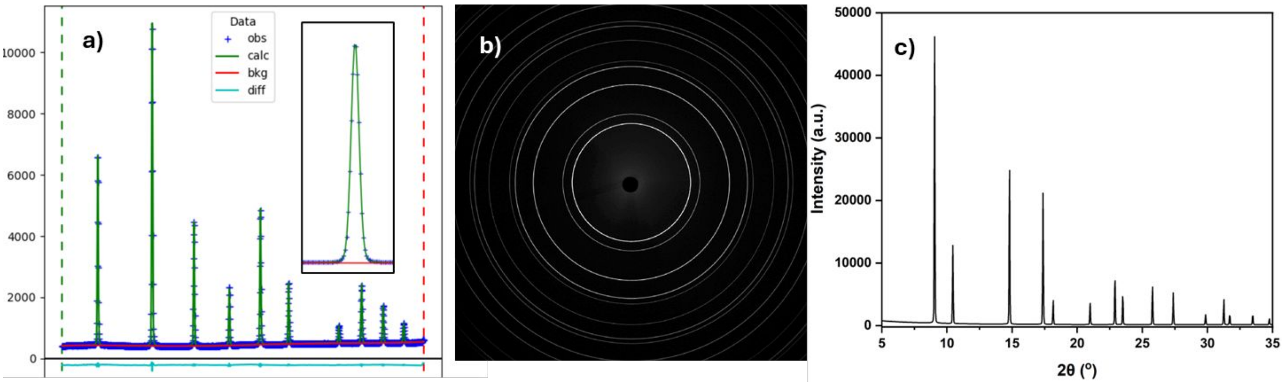

Figure SI2.2. a) Data and fit of the LaB6 pellet measured in Furnace 1 at 25 keV, used to generate an instrument template in GSAS II. A CIF file of NIST LaB6 600b standard was used for fitting, and measurement conditions and instrument parameters were applied. **b)** CeO_2 diffraction rings collected on Pixium area detector and c) the generated 1D 2θ pattern at 25 keV. Sample detector distance is 400 mm. Data visualisation was using DAWN software.[6]

During initial tests it was found the temperature of the Linkam cells was miscalibrated by a large margin. The distinctive PdO to Pd^0 phase transition should occur at 850 °C, shown by in situ lab XRD and TGA, but using the Linkam cells temperatures of > 1100 °C were required. Ideally, temperature calibration of the cells would be performed using reference samples with well-known phase transitions at discreet temperatures. This would have required online measurements though and could not be performed in the allocated one hour slot. Instead, a second high temperature thermocouple was inserted through the gas inlet and placed directly on the pellet during an offline temperature ramp, to better ascertain the actual sample temperature. Calibration curves were generated from this (Figure SI2.3), and used to find the temperature setpoints for the experiments.

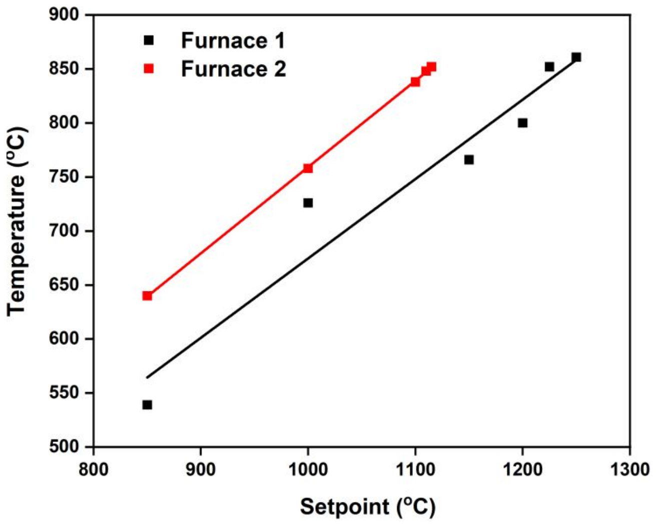

Figure SI2.3. Linkam cell temperature calibration data using two thermocouples, where the 'actual' temperature is given by the thermocouple in contact with the pellet surface.

SI 3 Ce K edge data correction and processing

Data collection at the Ce K edge at 40.4 keV is a new capability for the XMaS beamline. Small issues with the monochromator alignment meant the absorption edge energy had to be corrected by 60 eV, a higher correction than usually required at lower energy edges. The spectra were also subject to dampening effects, and this was best mitigated by applying a k_2 weighting to the EXAFS, despite the high scattering amplitude of Ce.

SI 4 Combined powder diffraction and X-ray absorption spectroscopy experiment

A combined XRD and XANES experiment was performed on the XMaS beamline (BM28) at ESRF. A sample environment cell was built and commissioned at Glasgow University in collaboration with XMaS, based on a design by Pete Chupas.[7] The cell consists of a stainless steel frame with two resistance wire heating elements, one above and one below a capillary containing the sample and an *in situ* thermocouple. The geometry allows data collection in both transmission and fluorescence geometry simultaneously, with a wide angular range for PXRD measurements. This is demonstrated by the schematic in Figure SI4.1 and also Figure SI4.2

which shows the cell prior to loading a sample, and Figure SI4.3 which shows the cell in use at 900°C.

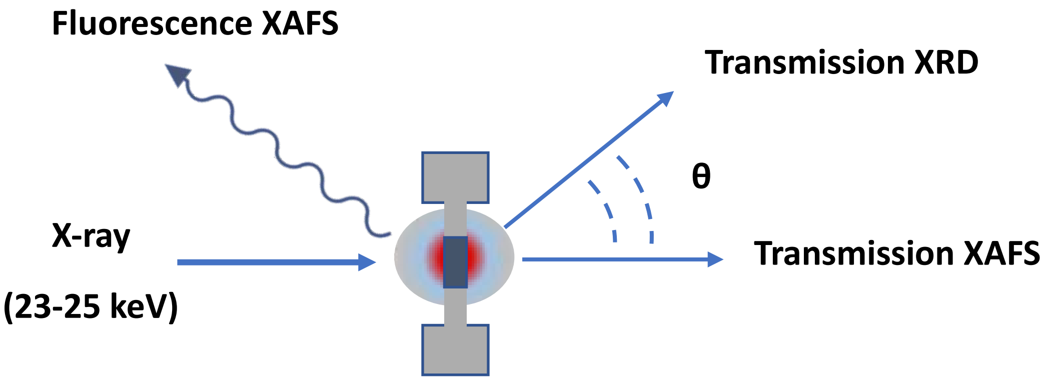

Figure SI4.1. A top-down schematic view of the *in situ* cell, showing its suitability for simultaneous X-ray absorption, both transmission and fluorescence measurements, and X-ray diffraction.

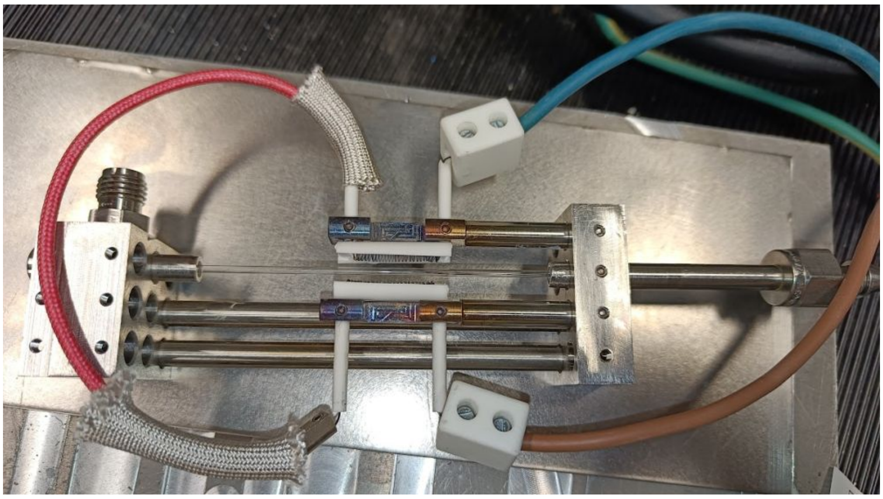

Figure SI4.2. Photograph of the cell prior to sample loading showing heating coils, ceramic radiation shields, ceramic insulation rods, electrical connection, and the capillary, with thermocouple inserted from the RHS through the Swagelok gas inlet. When the sample is

loaded, it sits between quartz wool plugs, and the capillary connections are made gas tight by silicone sealant (Silcoset 151).

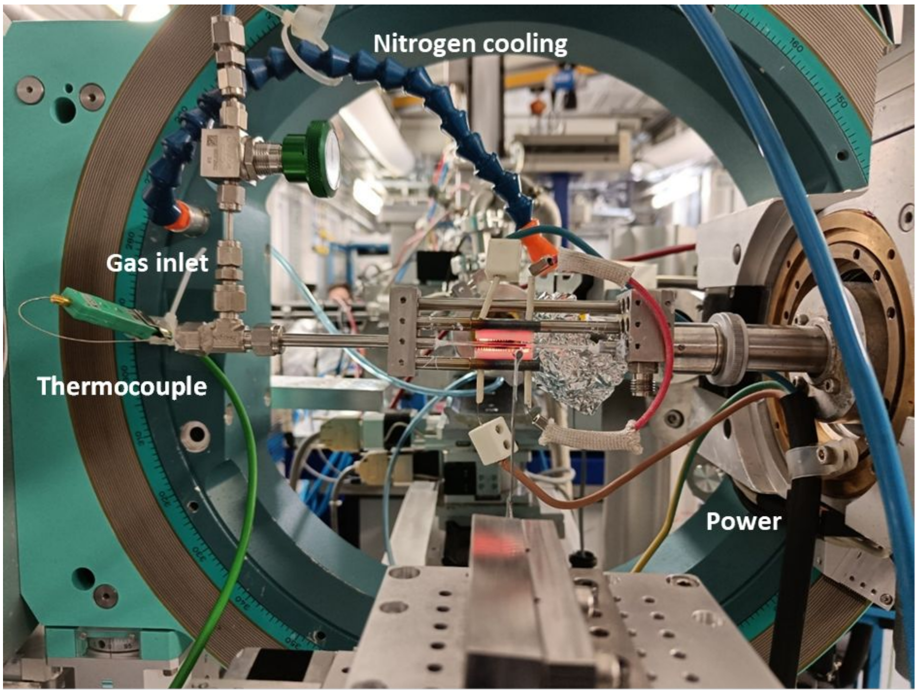

Figure SI4.3. Photograph of the *in situ* cell mounted on the goniometer, the high temperature glowing of the coils and thermal insulation from the shields can be seen. The fluorescence detector was covered by aluminium foil for heat protection, and a gas stream of nitrogen was also aimed at the fluorescence detector for cooling.

Both transmission and fluorescence yield XAS were performed at the XMaS beamline. The beamline optics consist of an LN2 cooled Si<111> monochromator, followed by a 1.4 metre long toroidal mirror to focus the beam at the centre of a Huber 6 circle diffractometer.[8] Both the 2D Pilatus 300K camera used for the XRD and ion chambers used for transmission XANES/EXAFS were mounted onto the diffractometer two theta arm, at two different angular positions, allowing each device to be positioned independently on the axis of the X-ray beam. By simply moving the two-theta arm of the diffractometer, it is easy to permit interleaving of both the XRD and XANES/EXAFS measurements. In this experiment XRD was measured at

24 keV, just below the Pd K edge, to prevent any effects from anomalous diffraction. Each image by the Pilatus camera covered just over 1° 2θ, so for the 14° time resolved patterns 10 images were collected. They were combined into one and converted into 1D 2θ versus intensity plots using the ESAproject software developed by Mark Dowsett.[9] The transmitted intensity was normalised to the intensity of the incident beam during this process, as detected by an ion chamber.

For the transmission XAS measurements, Oken ion chambers were used with a gas mix of Ar/Kr. For the fluorescence measurements, a Keytek single element Silicon drift diode was used at the Pd K edge, along with a Mirion 7 element Germanium detector for the Ce K edge measurements. Both of these detectors used a Falcon-X counting chain integrated into the beamline SPEC control system. Both edges required measurement in fluorescence mode due to the high absorption of X-rays by the ceria zirconia matrix.

SI 5 Dynamic OSC (DOSC) versus breakthrough method

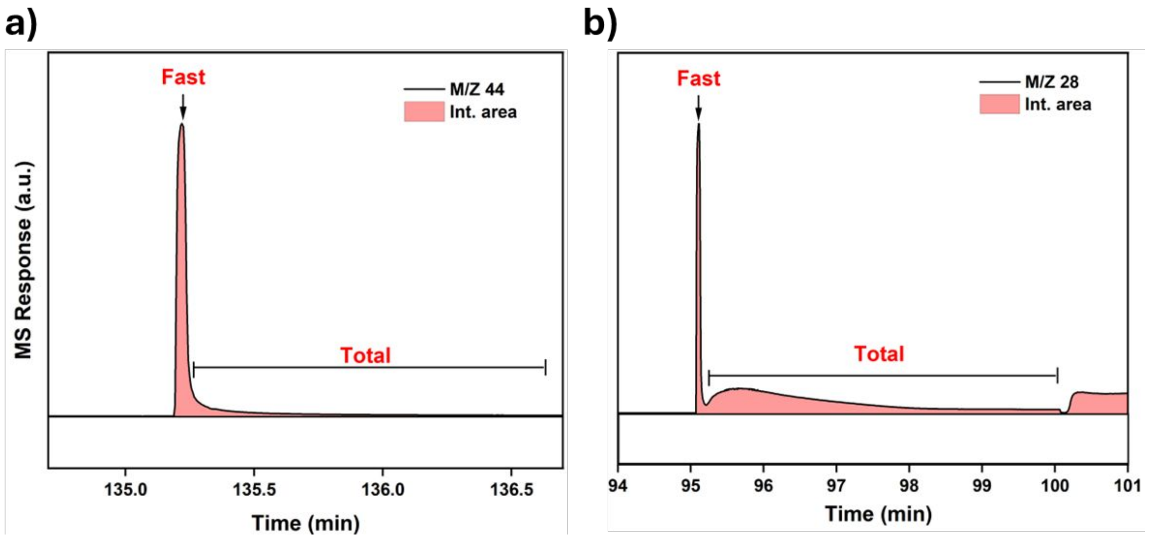

Figure SI5.1 Corrected MS response and integrated area for m/z 44 (CO_2) at during the reduction portion of a DOSC experiment for **a)** CZ1 and **b)** Pd/CZ1 at 700 °C

For ceria zirconia samples, a sharp CO_2 peak appears when the CO flow is started, which tails away until the CO flow is stopped at 300 s (Figure SI5.1a). The rate of oxygen removal becomes slower as the maximum extent of reduction is approached.[10] Pd/CZ1 however shows two peaks in the CO_2 trace, with the same short sharp peak at the start, but then another broad peak appearing once the first has started to tail off (Figure SI5.1b). This is suggestive of two different mechanisms, with the primary mechanism the same as for bare ceria zirconia. The second CO_2 forming reaction is likely the Boudouard reaction, with incoming CO reacting with reactive oxygen formed by the disproportionation of CO on the catalyst surface, represented below in Figure SI5.2. This has been observed for Rh/CeO_2 by Stubenrauch et al.,[11] for Pt/$CeZrO_2$ by Hickey et al.,[12] and CO oxidation in the absence of oxygen gas has been observed by Gredig et al.[13] and Matolin et al.[14]

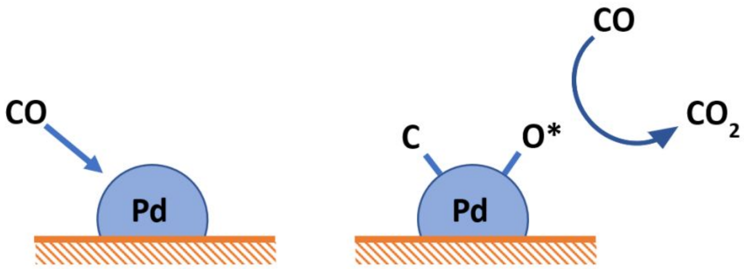

Figure SI5.2. Representative schematic of CO disproportionation and reaction with incoming CO on a supported Pd nanoparticle, a secondary CO_2 producing mechanism for Pd loaded samples. O* represents a single oxygen.

The absolute OSC of a sample measured by the DOSC method, compared to the same sample measured by the breakthrough method, are different. Therefore, comparing the OSC values recorded for CZ1 and CZ2, with those for Pd/CZ1 and Pd/CZ2, should be done with caution. The method of calculating OSC from breakthrough measurements is likely to lead to underestimation of the OSC, particularly when compared to the total OSC calculated by the DOSC method. Nevertheless, comparisons between CZ1 and CZ2, and between Pd/CZ1 and

Pd/CZ2, are believed to be unaffected by this, therefore the results of aging on each set of samples are real.

SI 6 OSC reproducibility, lattice oxygen calculations, and example MS results

Dynamic OSC tests of CZ1 were run 3 times, and the relative standard deviation over all temperatures averaged to 3.7 % and 3.8 % for total and fast OSC respectively, Figure SI6.1.

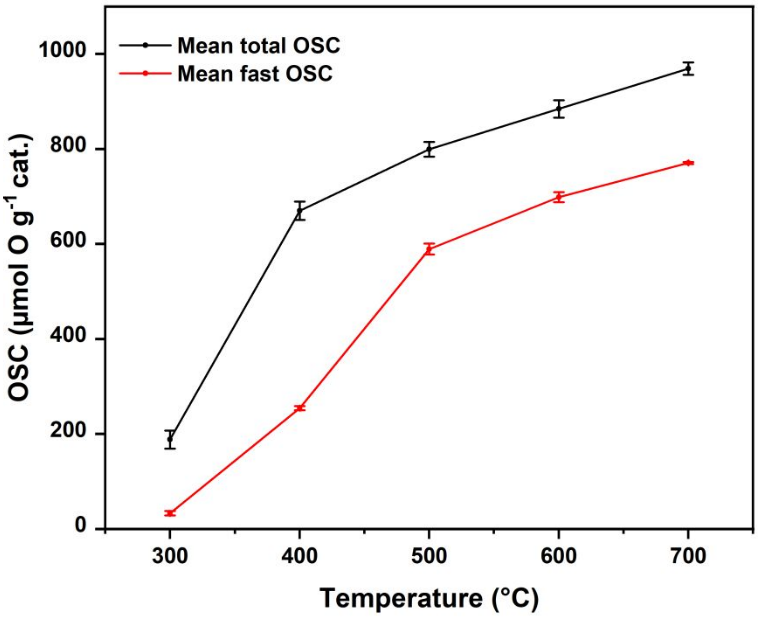

Figure SI6.1. Mean total and fast OSC of CZ1 from 3 repeats, with error bars showing the standard deviation from the mean.

To calculate % OSC utilisation of a sample, the theoretical maximum OSC was first calculated. The moles of Ce per g catalyst is determined. The reduction of two Ce^{4+} ions to Ce^{3+} ions are required to charge balance the loss of one O^{2-} ion. Therefore, the ratio of Ce:O in oxygen removal is 2:1. For a Pd containing sample, the maximum theoretical OSC from the mass of Pd in the sample is calculated and added to the value from the ceria zirconia. The reduction of one mole of PdO releases one mole of O*, hence the ratio of Pd:O in oxygen removal is 1:1.

An example of the MS response for CO, O_2 and CO_2 during one sequence using the breakthrough method is given in Figure SI6.2.

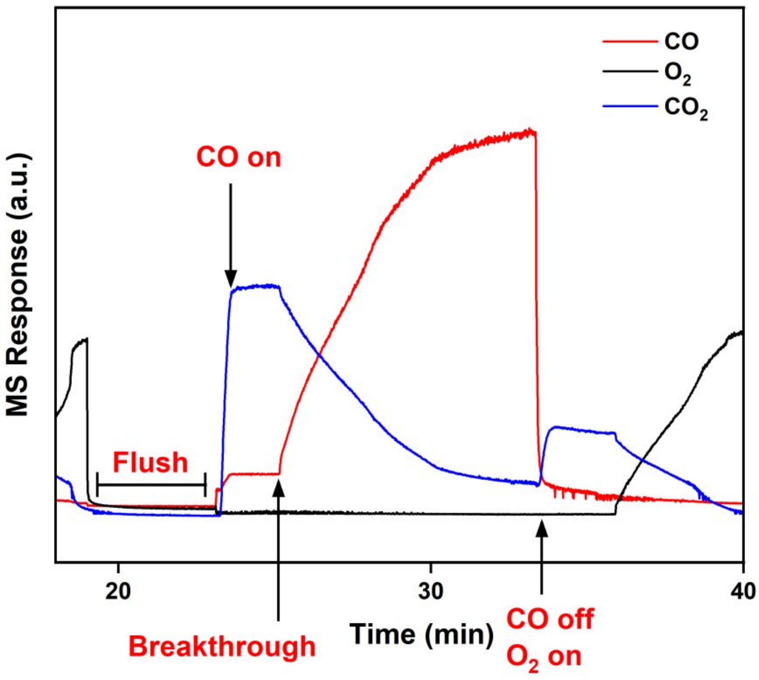

Figure SI6.2. Raw MS response of m/z 28 (CO), 32 (O_2) and 44 (CO_2) for Pd/CZ1 at 200 °C. OSC is calculated from the time between 'CO on' and 'breakthrough'. The small increase in m/z 28 during this period is due to CO_2 disproportionation inside the MS.

SI 7 Model exhaust gas composition

Table SI7.1 Composition of model exhaust gas mix and flow of each component. Suitable units, % or ppm, are used depending on concentration of species. Total flow was 3 L min^{-1} through the catalyst bed, and 2 L min^{-1} through the bypass, giving a combined flow of 5 L min^{-1}.

Gas	Concentration	Flow (mL min^{-1})
N_2 (2 MFCs)	balance	7250 + 1000
CO_2	14 %	1560
10% NO/N_2	2200 ppm	226
O_2	0.72 %	78
CO	0.8 %	93
H_2	0.27 %	29.3
10% C_3H_6/N_2	666 ppm	91
10% C_2H_6/N_2	333 ppm	35
CO (perturbed)	1.4 %	139
H_2 (perturbed)	0.47 %	52
O_2 (perturbed)	0.9 %	98
H_2O	4 %	

SI 8 Conversion and selectivity formulas

Conversions were calculated for CO, NO, total hydrocarbons (HCs) and individual hydrocarbons (ethane and propene). Conversions were calculated based on loss of reactant, rather than formation of product, using eq. 1:

$$\chi_A = \frac{[A]_{in} - [A]_{out}}{[A]_{in}}$$

where χ_A is the conversion of A,

$[A]_{in}$ is the concentration of A in the inlet, and

$[A]_{out}$ is the concentration of A in the outlet.

Inlet concentrations were taken from the 90 s baseline measurements. The experiment used gas perturbation to switch between a reducing and oxidising atmosphere every 3 s, meaning the inlet concentration of CO, H_2 and O_2 were also changed every 3 s. As the conversion of CO was calculated from the inlet concentration of CO, 20 values of $[CO]_{in}$ covering a time period of 6 s were averaged for the % conversion at each temperature point.

During the reaction NO was converted to the following major products: NH_3, NO_2, N_2O, and N_2. The selectivities were calculated using the outlet concentration of each product, the inlet concentration of NO from baseline measurements and the total conversion of NO, according to eq. 2:

$$S_{NH_3} = \frac{[NH_3]_{out}}{\chi_{NO}/_{100} \text{ x } [NO]_{in}}$$

where S_{NH_3} is the selectivity of NO to NH_3,

$[NH_3]_{out}$ is the concentration of NH_3 in the outlet,

$[NO]_{in}$ is the inlet concentration of NO, and

χ_{NO} is the conversion of NO.

Selectivity was calculated in this way for NH_3, NO_2 and N_2O. Selectivity to N_2 could not be calculated in this way as the majority of the feed gas concentration is N_2, hence it is not present in the outlet from the conversion of NO only. As there are only four main products to this reaction, the selectivity of N_2 was instead calculated according to eq. 3:

$$S_{N_2} = 100 - S_{NH_3} - S_{NO_2} - S_{N_2O}$$

Selectivities were only calculated when the total NO conversion passed 5 %.

SI 9 Ce K edge further data and Ce L3 edge data

Ce K edge

Figure SI9.1 shows the stacked Fourier transform Ce K edge data of CeO_2, $Ce_{0.75}Zr_{0.25}O_2$, and $Ce_{0.5}Zr_{0.5}O_2$ to demonstrate the effects of increasing Zr concentration on the second shell scattering path.

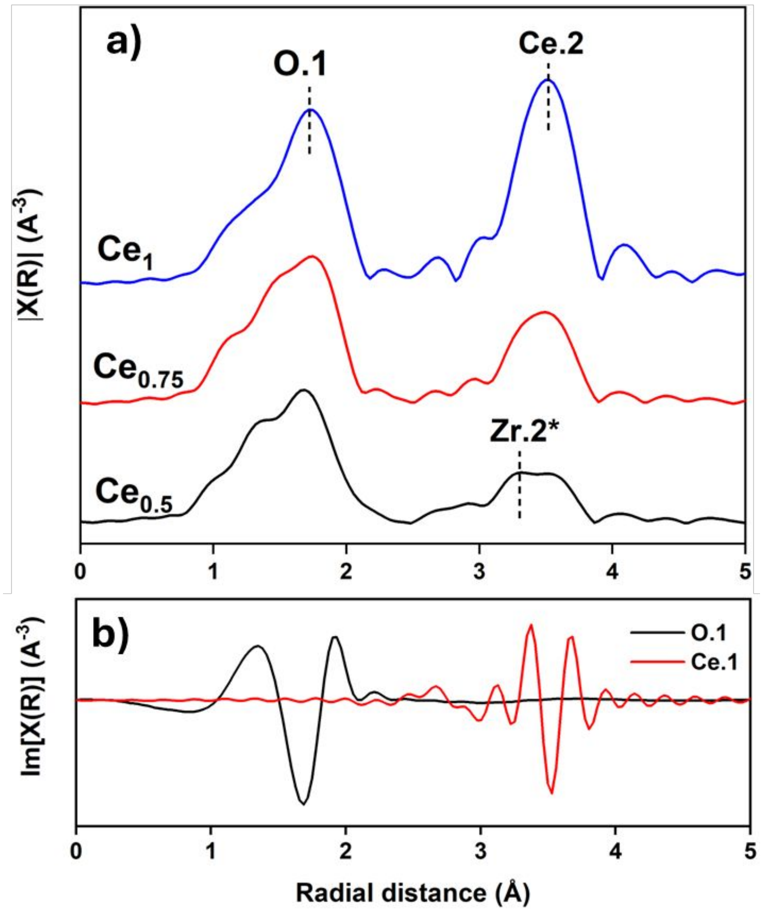

Figure SI9.1. a) the Fourier transformed Ce K edge data of $Ce_xZr_{1-x}O_2$, where x = 0.5, 0.75, and 1, plotted in magnitude in R space, and **b)** the O.1 and Ce.1 paths in CeO_2, plotted as the imaginary component in R space. The increasing second shell intensity at 3-4 Å (not phase corrected) is due to increasing contribution of the Ce.1 path as Ce fraction increases.

As the Ce fraction increases, the contribution of the second shell Ce-Ce path increases, the peak labelled Ce.2 in the R space plot. As expected, the amplitude of the single scattering first shell

Ce-O path remains similar for all three samples. These results are in line with those by Deguchi et al. who studied doped ceria systems.[15] As the amount of Zr increases from 0 to 0.5, contributions from a new scattering path emerge at 3.3 Å. This is a new Ce-Zr path. Given the difficulty in fitting EXAFS data where two different atoms share the same site in the unit cell, the Ce-Zr path was not fitted. However, Nagai combined Ce K and Zr K edges to analyse the Ce-Zr path, and calculated an estimated Ce-Zr distance of 3.735 Å, which they observed at the same position in their non-phase corrected data as the Ce-Zr path is observed in Figure SI9,1 above.[16]

The Ce-O and Ce-Ce path however were fitted, with an example of the fit for Pd/CeO_2 in Figure SI9.2.

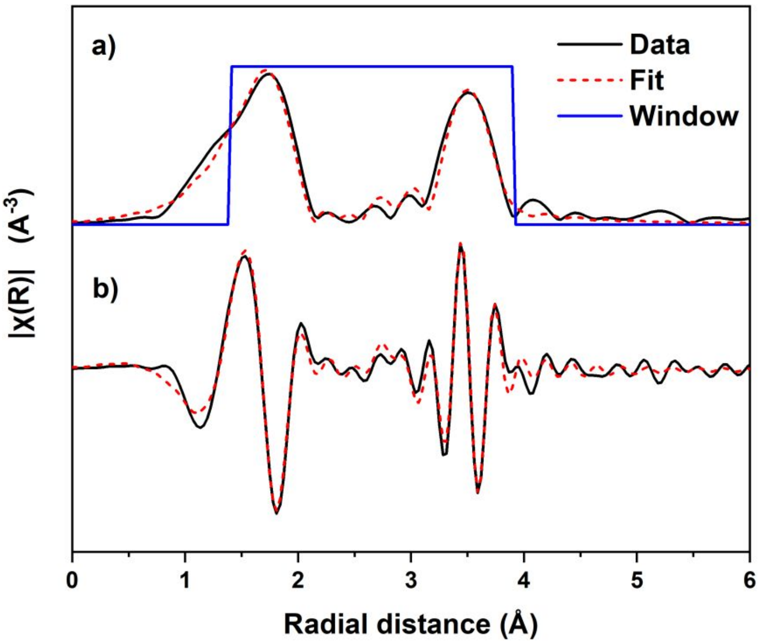

Figure SI9.2. k2 weighted Fourier transform data of Pd/CeO2 at the Ce K edge, plotted in R space, both the **a)** magnitude and **b)** imaginary component, data (black) and fit (red dash). Data is fitted to scattering paths from CeO2 only. A Ce foil was first refined to obtain the S_0^2 value.

The fit is slightly worse for the Zr doped samples as the unaccounted for Zr contribution increases, but the R_{fac} remained low. The calculated parameters from the fits of CeO_2,

$Ce_{0.75}Zr_{0.5}O_2$ and $Ce_{0.5}Zr_{0.5}O_2$, containing 5 wt % Pd, are displayed in Table SI9.1 below. The σ^2 values are high for oxygen, though the Ce K edge is less suited to 1st shell scattering than the Ce L_3 edge. The Ce:Zr 1:5 samples could not be fit given the low Ce concentration and the domination of a Ce-Zr path which could not be fit.

Table SI9.1. Fit results of Ce K edge EXAFS to a 1st shell oxygen path and a 2nd shell Ce path

Sample	R_{fac}	DeltaE (eV)	Path	Coordination number	Bond Length (Å)	σ^2
Pd/CeO_2	0.019	-5.9 ± 0.4	CeO_2: O	9.49 ± 0.42	2.319± 0.004	0.008 ± 0.001
			CeO_2: Ce	9.49 ± 0.50	3.865 ± 0.003	0.005 ± 0.000
$Pd/Ce_{0.75}Zr_{0.25}O_2$	0.025	-7.4 ± 0.5	CeO_2: O	8.69 ± 0.34	2.328± 0.008	0.008 ± 0.001
			CeO_2: Ce	5.63 ± 0.48	3.824 ± 0.006	0.005 ± 0.000
$Pd/Ce_{0.5}Zr_{0.5}O_2$	0.029	-6.7 ± 0.8	CeO_2: O	9.62 ± 0.47	2.316± 0.004	0.011 ± 0.001
			CeO_2: Ce	2.81 ± 0.32	3.812 ± 0.005	0.004 ± 0.000

Amplitude refined to 0.88. Fit window was 2 < k < 14 , 1.4 < R < 3.9, No. independent points = 19

Figure SI9.3 shows the linear relationship between increasing Ce substitution, and decreasing number of second shell Ce coordinating to the absorbing Ce atom. The reduction in C.N. seems particularly high, but is consistent with the literature. From the above figure, substituting 25 % of Ce with Zr decreased the C.N. by 40 %. In the study mentioned above, Deguchi et al. found substituting 30 % of Ce with Gd decreased C.N. also by 40%.[15] From the graph, the C.N. halves again when another 25 % of the Ce is substituted, giving a C.N. of only 2.5. The real C.N. may not be quite so low, but increased disorder and reduced crystallinity in the highly doped samples may be affecting amplitudes by potentially adding destructive interference between the Ce-Zr path and the Ce-Ce path.

The decrease in the Ce-Ce distance with increasing Zr concentration is consistent with changing lattice size. PXRD showed increasing Zr content decreases lattice size in a linear fashion, and all cation-cation distances should decrease in a shrinking lattice.

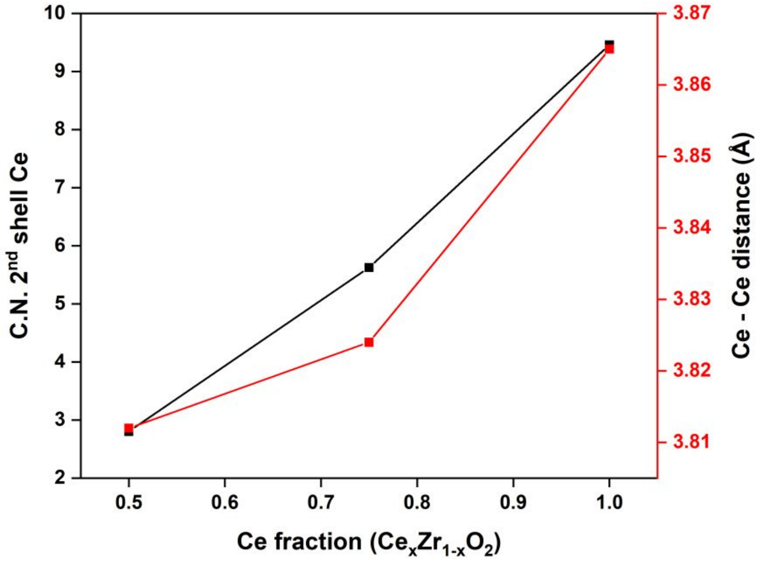

Figure SI9.3. Ce-Ce distance and coordination number as a function of Ce content in $Ce_xZr_{1-x}O_2$ from ex situ Ce K-edge data obtained at XMaS at the ESRF.

Ce L_3 edge

The Ce L_3 edge was measured to confirm the oxidation state of Ce in the fresh samples, Figure SI9.4. Typically this would be performed using linear combination fitting, as for the Pd K edge, however this method is unsuitable for Ce L_3 data, and so a manual fitting method was used, described below.[17,18] This method calculated 100% Ce^{4+} for both fresh CZ1 and fresh CZ2.

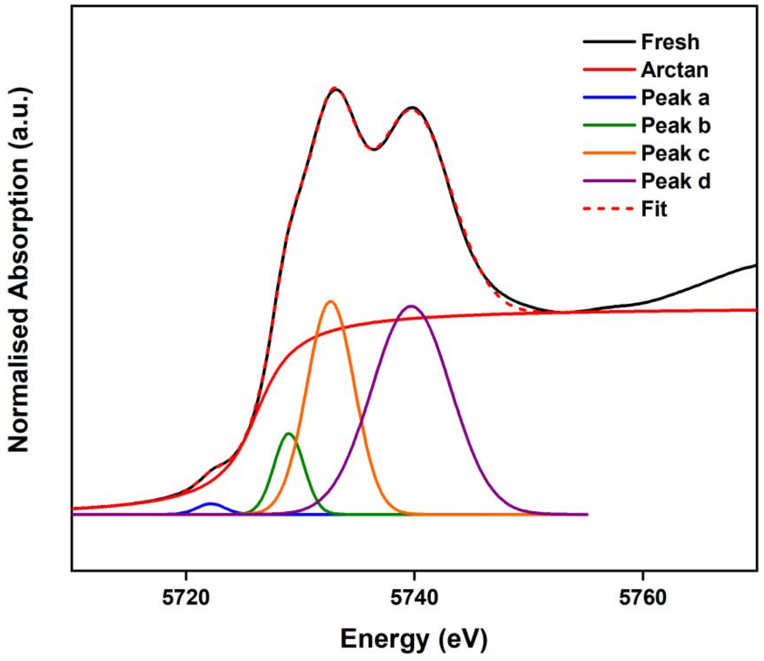

Figure SI9.4. XANES spectra of fresh CZ1 measured at the Ce L_3 edge on B18 at DLS. The spectra has been fitted with an arctan function for the edge jump and four gaussian features for the four corresponding electronic transitions, detailed below.

An arctan function was used to account for the rise in intensity at the absorption edge. Four gaussian functions were fit for four electronic transitions from the $2p_{3/2}$ state. Peak a fits the pre-edge feature arising from partly allowed excitation to empty 4f states. Peak b is a small shoulder caused by crystal field splitting within cubic fluorite ceria, however at this exact energy in Ce^{3+} a much larger peak is observed due to the 2p → $4f^1$ transition.[17,18] The relative area of a peak at this energy is used to calculate Ce^{3+} concentration. Given the dual origin of intensity at this energy, it is essential to first subtract the contribution from Ce^{4+}. Peak c and d form the characteristic doublet and arise from the 2p → $f4^{0,1}$ transition.[17]

SI 10 Phase assignment of single phase ceria zirconias

Regarding the powder diffraction measurements, the phase assignment of ceria zirconia is not unanimously agreed in the literature. Phase identification is particularly complex as the fluorite phase, which has $Fm\bar{3}m$ symmetry (space group No. 225), and one of the tetragonal phases, with $P4_2/nmc$ symmetry (space group No. 137, often described as t"), are indistinguishable by X-ray diffraction.[19] The t" phase has the symmetry elements of the fluorite phase, with three equal unit cell axes, but with a slight distortion in the positions of the oxygen in the sublattice, brought about by the substitution of larger Ce^{4+} with smaller Zr^{4+} ions.[20,21] Another tetragonal phase is often observed which is more distinctive by PXRD and is most frequently formed upon aging of the pseudo-fluorite phase, as observed in the later sections on catalyst aging.[19] In summary, the fresh ceria zirconia catalysts here are be described as having a 'pseudo-cubic' phase, and refined with $Fm\bar{3}m$ symmetry.

SI 11 Raman spectroscopy for phase identification

Raman spectra were measured on a Horiba LabRam HR photospectrometer at ambient temperature in atmospheric air, in the range 50-3500 cm^{-1} using a 532 nm laser with a x50WD lens. Spectra were not normalised due to a 10 fold difference in absolute intensity and differences in baseline features.

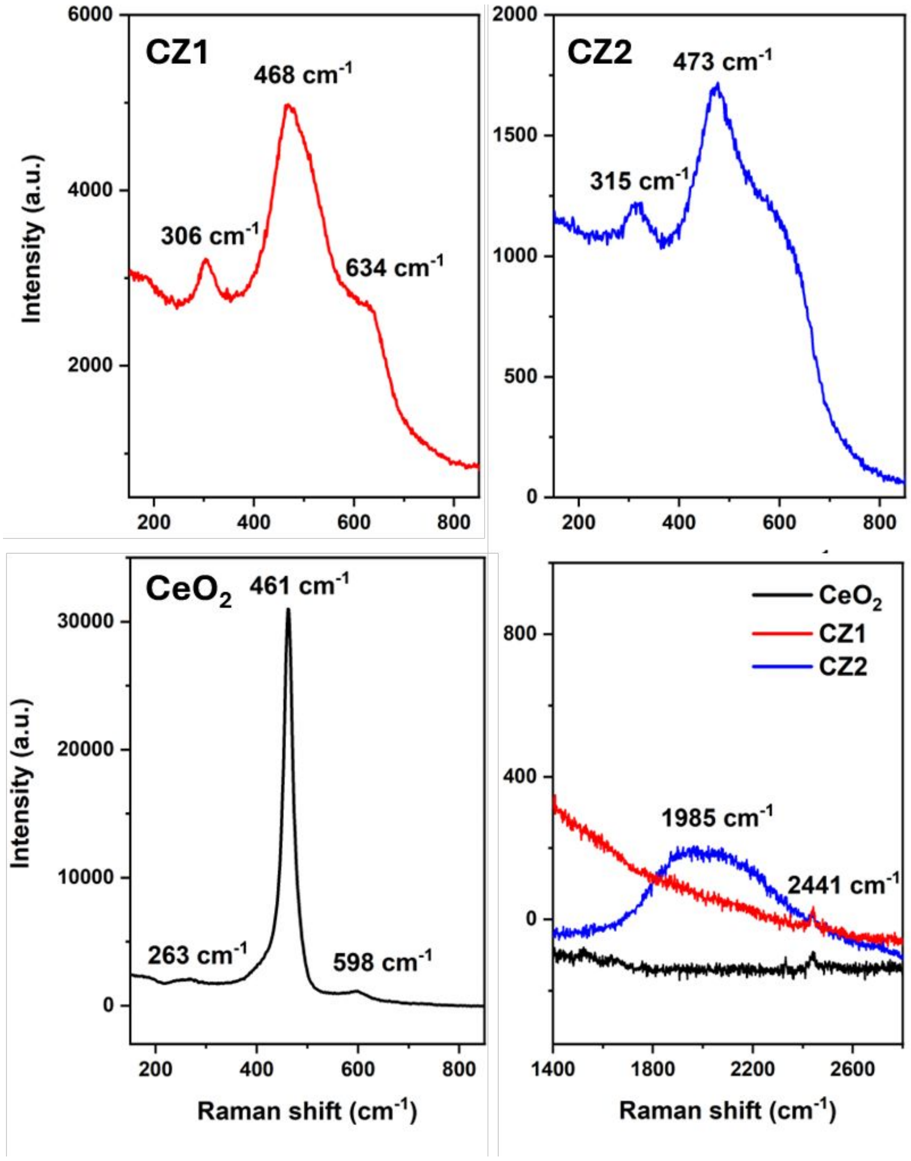

Figure SI11.1. Raman spectra of CeO_2, CZ1 and CZ2 measured with a 532nm laser. The three are compared at higher wavenumber in the bottom right figure. Spectra were not normalised.

Pure CeO_2 with an $Fm\overline{3}m$ phase has one Raman active mode resulting in an F_{2g} band, observed here at 461cm^{-1}.[22] The Raman band at 598 cm^{-1} in pure CeO_2 are symmetrically forbidden for

the cubic fluorite phase but become allowed as symmetry is lowered due to oxygen vacancies and structural defects caused by oxygen displacement. The Raman band at 263 cm^{-1}, and the shoulder at approximately 400 cm^{-1}, are observed only for nanocrystalline CeO_2 with a high proportion of clean CeO_2 surfaces. They are the result of Ce – O stretching in the top most atomic layers.[22]

The blue-shifting of the F_{2g} band in CZ1 and CZ2 compared to CeO_2 is a result of lattice shrinkage as Ce^{4+} ions are substituted by smaller Zr^{4+} ions. The broadening of the F_{2g} band is due to structural defects, and the lower symmetry of the t" phase of $CeZrO_2$ compared to the cubic phase of CeO_2.[19] Note that crystallite size is also proportional to peak width and intensity,[22] however the crystallite sizes determined earlier by PXRD were 10, 10 and 16 nm for CeO_2, CZ1 and CZ2 respectively, hence too similar to be responsible for the huge differences in peak shape. As discussed in the main text, the t" phase of fresh CZ1 and CZ2 is tetragonal but with a c/a ratio of 1, hence is referred to as 'pseudo-cubic'. The difference between t" and c is observed most clearly in the Raman spectra above, where CZ1 and CZ2 show a new Raman band at just above 300$cm^{-1,}$ associated with the tetragonal $P4_2/nmc$ symmetry group. The shoulder in the F_{2g} band, strongest in CZ1, is also associated with the t" phase, and is not present in pure CeO_2.

The broad Raman band centred at 1985 cm^{-1}, observed only for CZ2, indicates trivalent rare earth metal ions, corresponding to a $^2F_{5/2} \rightarrow {}^2F_{7/2}$ electronic transition.[22] In the referenced publications, Ce^{3+} cause this transition, however CZ2 is doped specifically with redox inactive RE^{3+}, whereas other characterisation confirmed all the Ce is in a +4 oxidation state in the fresh sample.

SI 12 PXRD pattern of CZ2, refinement details of both CZ1 and CZ2

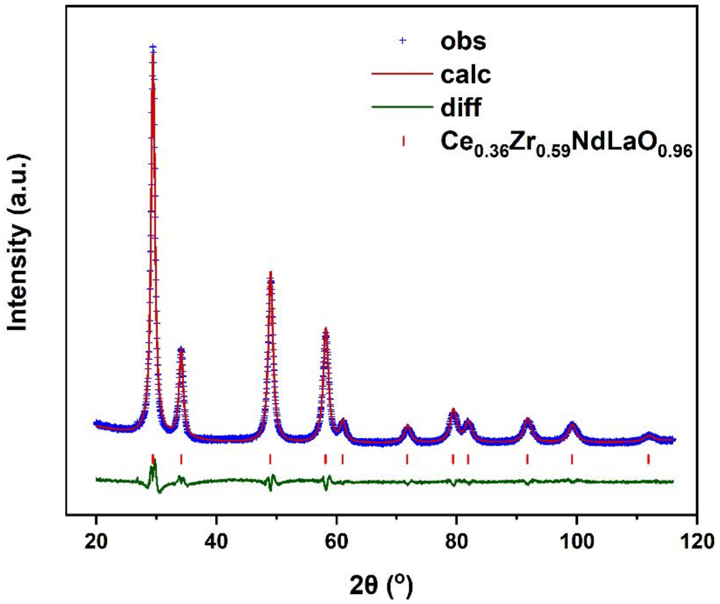

Figure SI12.1. Room-temperature profile fit from Rietveld refinement of the structure of CZ2 against PXRD data. R_w = 3.8%

Table SI12.1. Selected crystallographic data from Rietveld refinement of CZ1 ($Ce_{0.5}Zr_{0.5}O_2$) and CZ2 ($Ce_{0.36}Zr_{0.}59Re(+3)_{0.05}O_{0.96}$).

**Support**	**Latt. Param. (a = b = c)**	**Crystallite size**	**U_{iso} of cations**	**U_{iso} of oxygen**	**wR**
CZ1	5.280(1) Å	10 nm	0.0217	0.0270	4.14 %
CZ2	5.260(1) Å	16 nm	0.0217	0.0270	3.84 %

The U_{iso} of all cations were made equivalent. Cation occupancies were fixed to reflect intended stoichiometry; oxygen occupancies were fixed to maintain charge neutrality. Weight of residuals (wR), and estimated standard deviations (esds), are given.

SI 13 Particle size by TEM

Particle size distribution of primary particles, or crystallites, determined from the TEM images, for Pd/CZ1 and Pd/CZ2 fresh and aged are shown in Figure SI13.1, with an example of the crystallites measured in SI13.2. Mean calculated sizes were 7.5, 27.5, 11.3 and 22.9 nm for Pd/CZ1 fresh, aged, and Pd/CZ2 fresh, aged respectively. Whilst these are for Pd loaded samples, PXRD showed the crystallite size of the CZ to be unaffected by Pd impregnation.

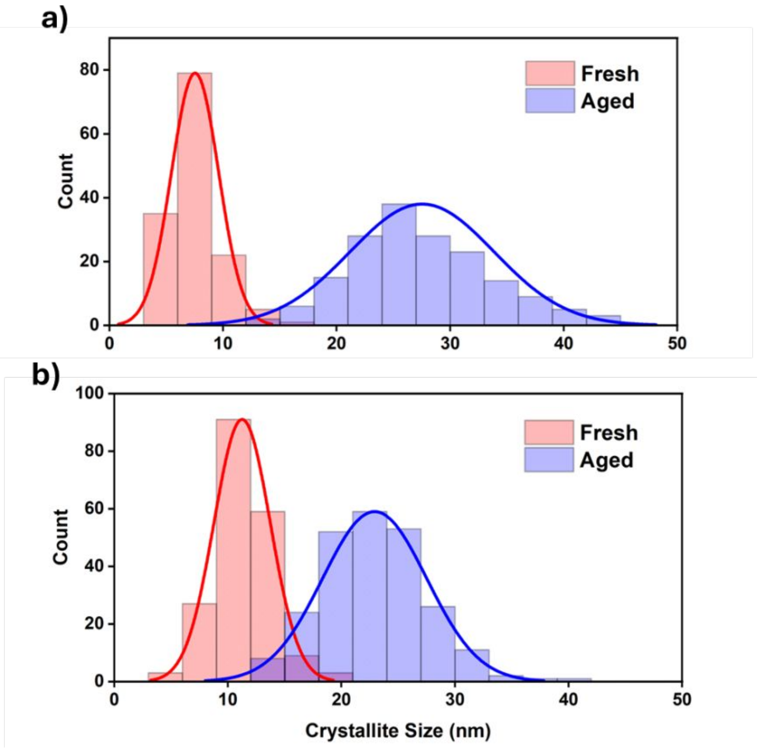

Figure SI13.1. The particle size distribution of crystallites of **a)** Pd/CZ1 and **b)** Pd/CZ2, fresh and aged 950°C 12h.

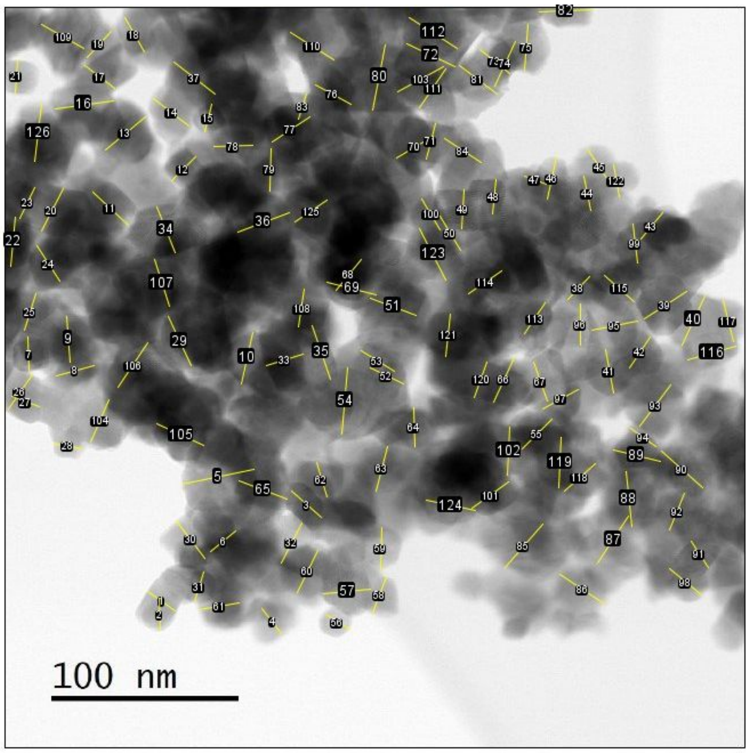

Figure SI13.2. Example extraction of crystallite size from aged Pd/CZ2 at 100 nm magnification using ImageJ.[23]

SI 14 Full profile refinements of aged CZ1 and CZ2

Rietveld refinement of CZ1 and aged CZ2, aged in air at 1150 °C for 24h. These conditions were chosen for full profile analysis of CZ1 rather than the 950 °C aging used for other ex situ characterisation, because higher levels of phase separation mean less peak overlap, hence phases can be assigned and data fit with more confidence. Full refinement details are below each figure.

CZ1:

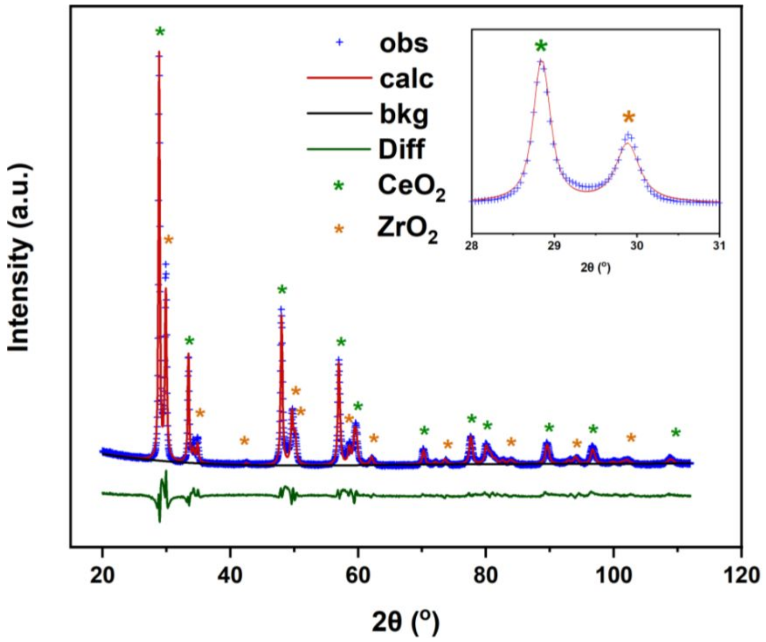

Figure SI14.1. Room-temperature profile fit from Rietveld refinement of the structure of CZ1 ($Ce_{0.5}Zr_{0.5}O_2$) aged at 1150 °C for 24h against PXRD data. The data is fit to a cubic $Fm\bar{3}m$ ceria[24] and tetragonal P42/nmc zirconia phase.[25]

Table SI14.1. Refined atomic parameters of CZ1 aged 1150 °C for 24h. Fit to a cubic $Fm\bar{3}m$ CeO2 phase, lattice parameter 'a' = 5.360(1) Å, crystallite size 79 nm, and a tetragonal P42/nmc ZrO2 phase, lattice parameter 'a' = 3.639(1) Å 'c' = 5.242(2) Å, crystallite size 48 nm. Weight fraction 52 % and 48 % respectively.

**Element**	**Phase**	**Site Symmetry**	**x**	**y**	**z**	**U_{iso} ($Å^2$)**	**Occupancy**
Ce^{4+}	$Fm\bar{3}m$	m3m	0	0	0	0.0210	1.0
O		-43m	0.25	0.25	0.25	0.0250	1.0
Zr^{4+}	$P4_2/nmc$	-4m2(z)	0.75	0.25	0.25	0.0220	1.0
O		mm2(z)	0.25	0.25	0.45	0.0361	1.0

wR = 4.11 %.

The phase separation of CZ1 results in a ceria rich cubic phases with small zirconia components, and vice versa for the zirconia rich tetragonal phase. This is evidenced by the lattice parameters of each phase in the above fit. The ceria lattice is slightly smaller than that of pure CeO_2 as measured previously, 5.36 Å compared to 5.41 Å, and the zirconia lattice is slightly larger than usually found in the literature, 3.64 Å compared to 3.59 Å for the 'c' axis.[25] The quantity of substitution in each phase based on these lattice values is expected to be small, but the quality of fit and number of other changing parameters was not sufficient to allow the compositions to be refined, hence the use of pure phase CeO_2 and ZrO_2. Instead, the calibration curve from SI 1 relating Ce content to lattice parameter can be used to extract the Ce fraction of the cubic phase. It estimates a stoichiometry of $Ce_{0.81}Zr_{0.19}O_2$. If no amorphous material formed, the tetragonal phase would have a corresponding composition of $Ce_{0.19}Zr_{0.81}O_2$. The crystallite size of the ceria and zirconia phase was 79 and 48 nm respectively, compared to 10 nm for the fresh mixed phase.

CZ2:

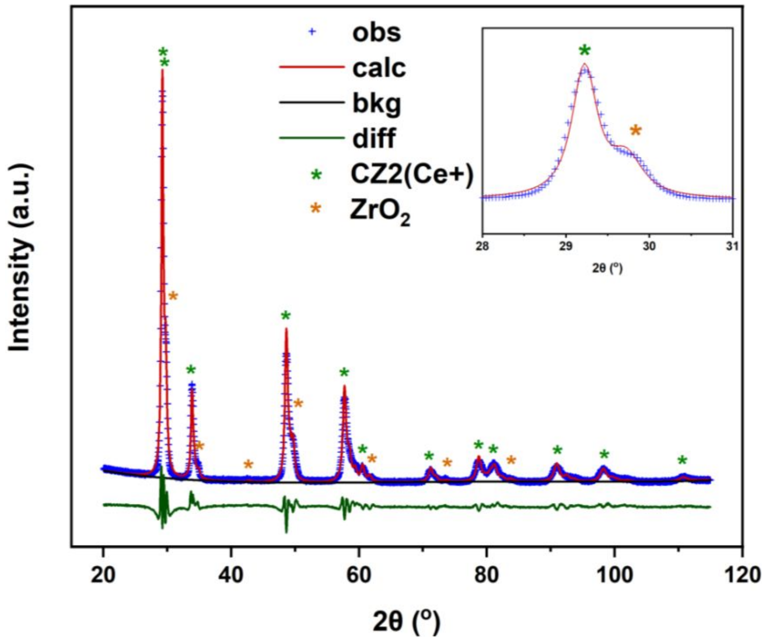

Figure SI14.2. Room-temperature profile fit from Rietveld refinement of the structure of CZ2 ($Ce_{0.36}Zr_{0.59}Re(III)_{0.05}O_2$) aged at 1150 °C for 24h against PXRD data. An inset shows the reflection at 29°, previously a single peak, now has a broad shoulder from an overlapping reflection. The data is fit to a cubic Fm$\bar{3}m$ ceria zirconia and a tetragonal $P4_2/nmc$ zirconia phase.

Table SI14.2. Refined atomic parameters of CZ2 aged 1150 °C for 24h. Fit to a cubic Fm$\bar{3}$m ceria zirconia phase, lattice parameter 'a' = 5.299 Å, crystallite size 63 nm, with occupancies not equal to the same phase of the fresh samples, and a tetragonal $P4_2/nmc$ ZrO_2 phase, lattice parameter 'a' = 3.709 Å 'c' = 5.146 Å, crystallite size 24 nm. Weight fraction 68 % and 52 % respectively.

**Element**	**Space group**	**Site Symmetry**	**x**	**y**	**z**	**U_{iso} ($Å^2$)**	**Occupancy**
Ce^{4+}	Fm$\bar{3}$m	m3m	0	0	0	0.0217	0.542
Zr^{4+}		m3m	0	0	0	0.0217	0.342
RE^{3+}		m3m	0	0	0	0.0217	0.039

O		-43m	0.25	0.25	0.25	0.0270	0.938
Zr^{4+}	$P4_2/nmc$	-4m2(z)	0.75	0.25	0.25	0.0217	1.0
O		mm2(z)	0.25	0.25	0.45	0.0270	1.0

wR = 7.72 %, esds = 0.0003.

The new composition of the cubic phase was $Ce_{0.53}Zr_{0.34}RE(+3)_{0.051}O_{0.94,}$ involving an increase in the Ce/Zr ratio from 0.6 to 1.6. The tetragonal phase had to be fit using pure ZrO_2, although its lattice parameter, 3.71 Å for the 'c' axis compared to the literature value 3.59 Å, indicated residual Ce.[25] Based on lattice size alone, it appears more Ce was left in the zirconia rich phase for CZ2 than for CZ1. Unfortunately, the composition could not be determined as the peaks are highly overlapping, and fitting resulted in impossible values. The calculated crystallite size of the cubic and tetragonal phase was 63 and 24 nm respectively compared to the 16 nm of the fresh mixed phase.

SI 15 Surface area measurements of CZ vs Pd/CZ

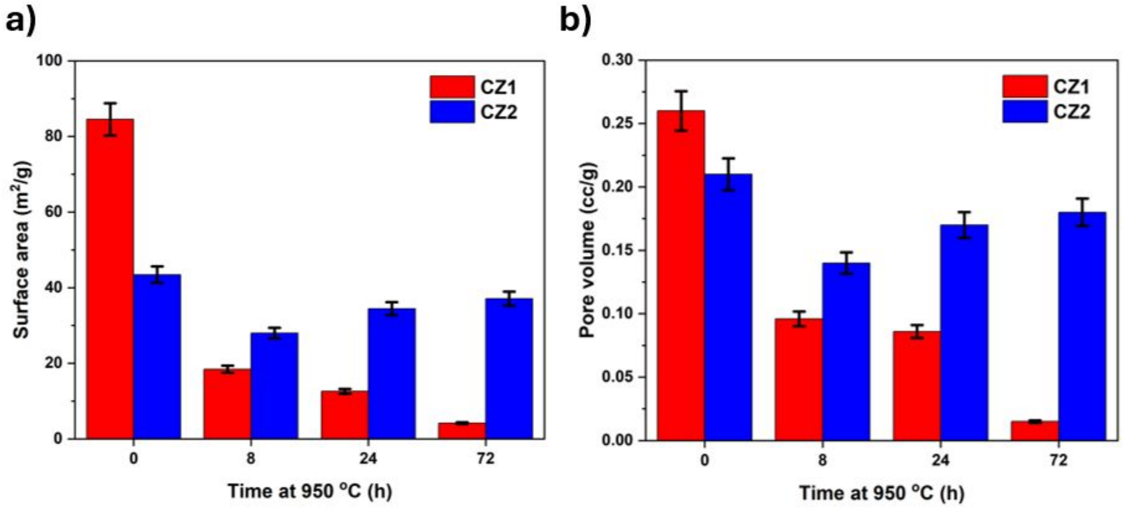

Figure SI5.2. a) surface area and **b)** pore volume from nitrogen sorption measurements of (red) CZ1 and (blue) CZ2, fresh, and aged at 950°C for 8, 24 and 72 h. Error was calculated from 3 repeated measurements of fresh CZ1 and was 5 % for surface area and 6 % for pore volume.

The surface area and pore volume values for CZ2 aged between 8 – 72 h show an apparent increase over time, but this is likely to be a combination of instrumental and experimental error.

Table SI15.1 Average surface area, pore volume and pore size of CZ1 and CZ2 compared to Pd/CZ1 and Pd/CZ2 prepared by an incipient wetness method.

	**Surface area (m^2 g^{-1})**	**Pore volume (cc g^{-1})**	**Pore size (Å)**
**CZ1**	85 ± 4	0.26 ± 0.02	28 ± 2
**Pd/CZ1**	62 ± 3	0.20 ± 0.01	36 ± 2
**CZ2**	43 ± 2	0.21 ± 0.01	16 ± 1
**Pd/CZ2**	35 ± 2	0.16 ± 0.01	19 ± 1

SI 16 TEM imaging and EDS/EELS of Pd/CZ1 and Pd/CZ2, fresh and aged

Note that Pd/PdO is expected to be difficult to observe on such a highly absorbing element as cerium, hence this may contribute, along with high dispersions, to the invisibility of Pd/PdO nanoparticles on the ceria zirconia crystallites.

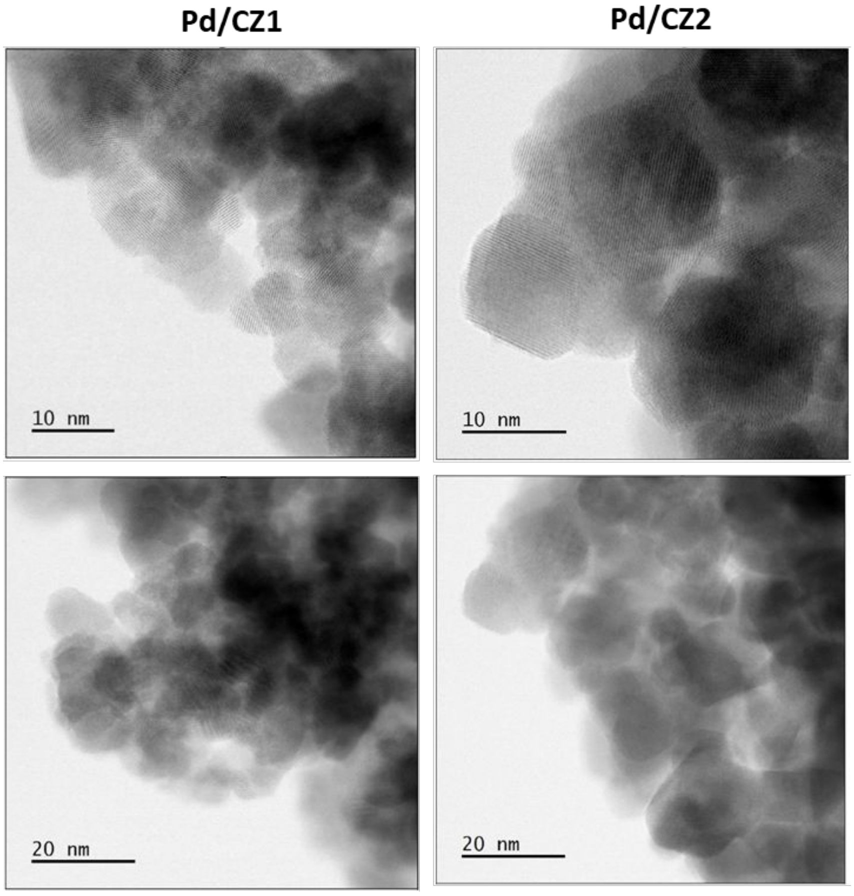

Figure SI16.1. Brightfield TEM images of Pd/CZ1 and Pd/CZ2, fresh, at two different magnifications (10 nm and 20 nm)

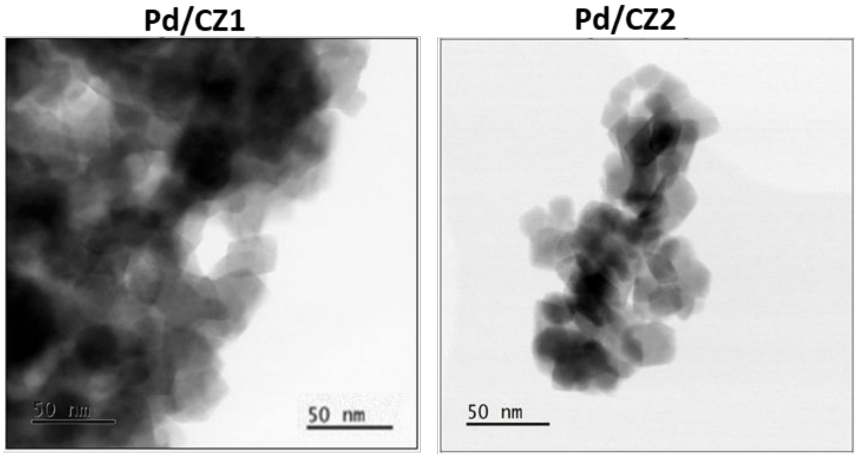

Figure SI16.2. Brightfield TEM images of Pd/CZ1 and Pd/CZ2, aged (950°C, 12h, air).

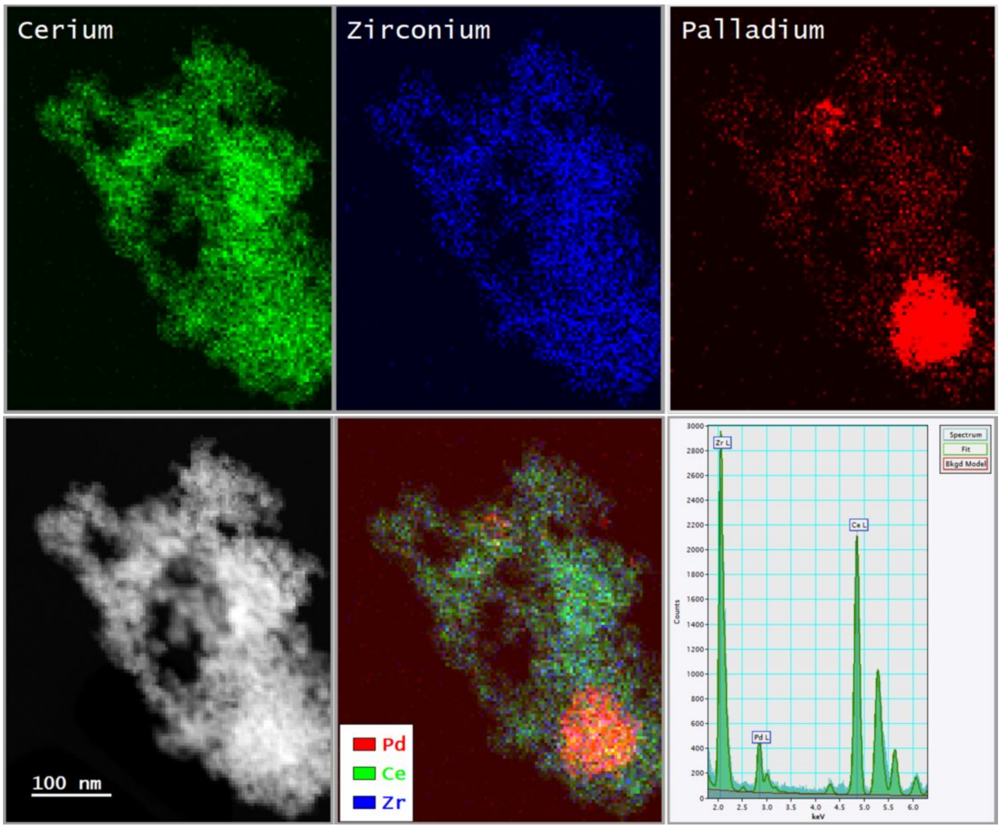

Figure SI16.3. Full elemental maps of Pd/CZ1, fresh, at lower magnification, by TEM coupled with EDS.

The above figure shows the concentrated region of Pd over a circa 100nm area, though with seemingly diffused Pd over the rest of the sample surface. The Ce and Zr are homogeneously mixed.

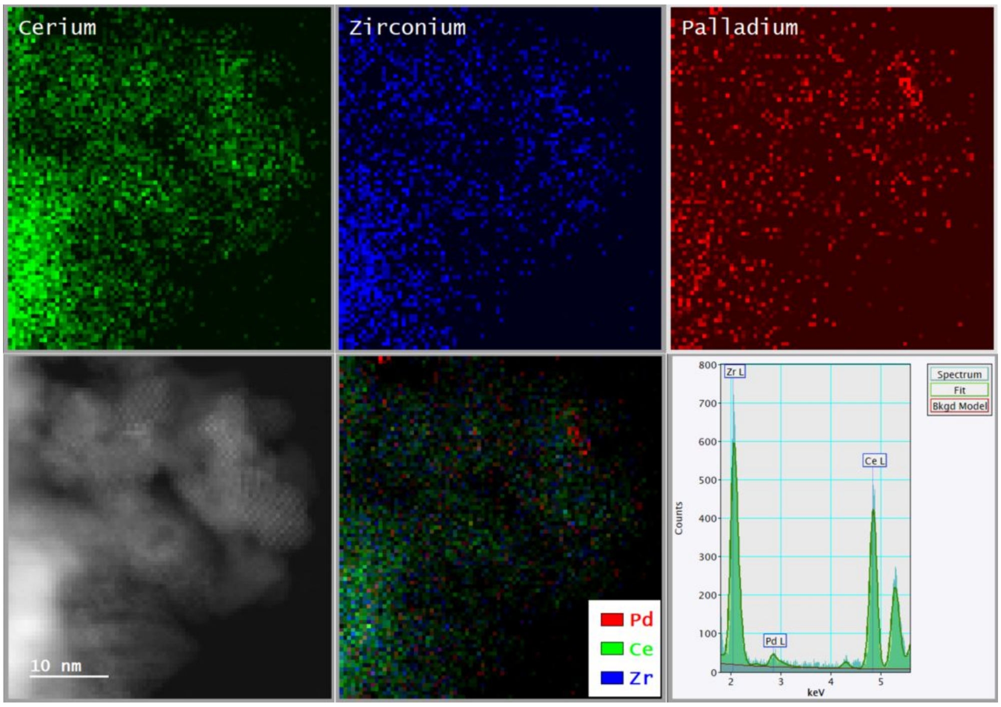

Figure SI16.4. Full elemental maps of Pd/CZ1, fresh, at higher magnification, by TEM coupled with EDS.

The above figure shows Pd is present over the CZ crystallites, confirming the background dispersion beyond the highly concentrated regions shown above. The Pd L signal is low, but is above the noise level.

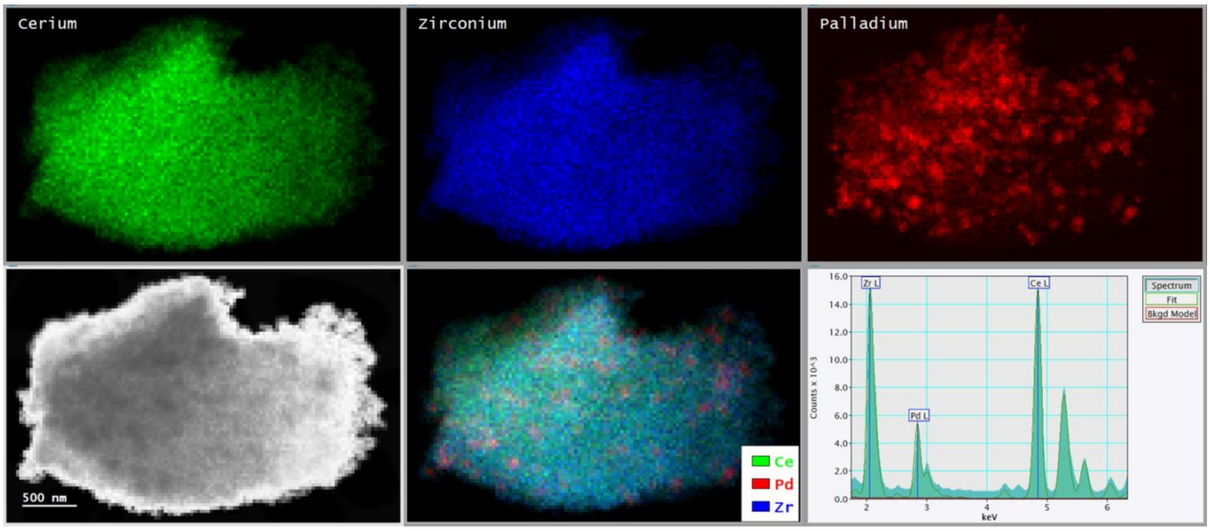

Figure SI16.5. Full elemental maps of Pd/CZ1, aged (950°C, 12h, air), at low magnification, by TEM coupled with EDS.

The above figure shows Pd agglomeration has occurred with aging, resulting in areas of high and low Pd concentration. The Ce and Zr remain homogeneously dispersed at this scale. Higher resolution TEM and PXRD demonstrate that the palladium particles continue to be formed of small (20-30 nm) individual crystallites, with no >100 nm Pd particles observed as would be indicated by the electron mapping above. Refer back to SI13.2 for HRTEM, and Figure 5 in the main text for PXRD.

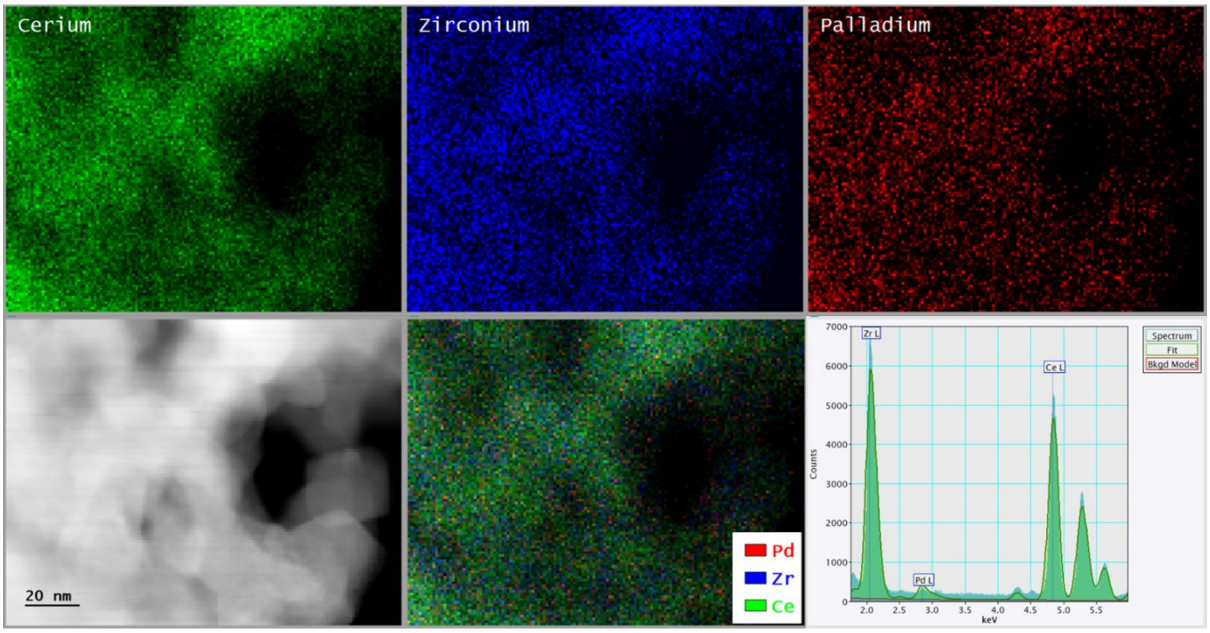

Figure SI16.6. Full elemental maps of Pd/CZ1, aged (950°C, 12h, air), at higher magnification, by TEM coupled with EDS.

The above figure shows that, despite the agglomeration observed at low magnification, there remains a dispersed layer of Pd over the crystallites, presumably with more or less total Pd in different regions. The Pd signal is low, indicating this is an area of low Pd concentration, but is above the noise level. Again the Ce and Zr remain elementally well dispersed, despite the phase separation occurring within the crystallites.

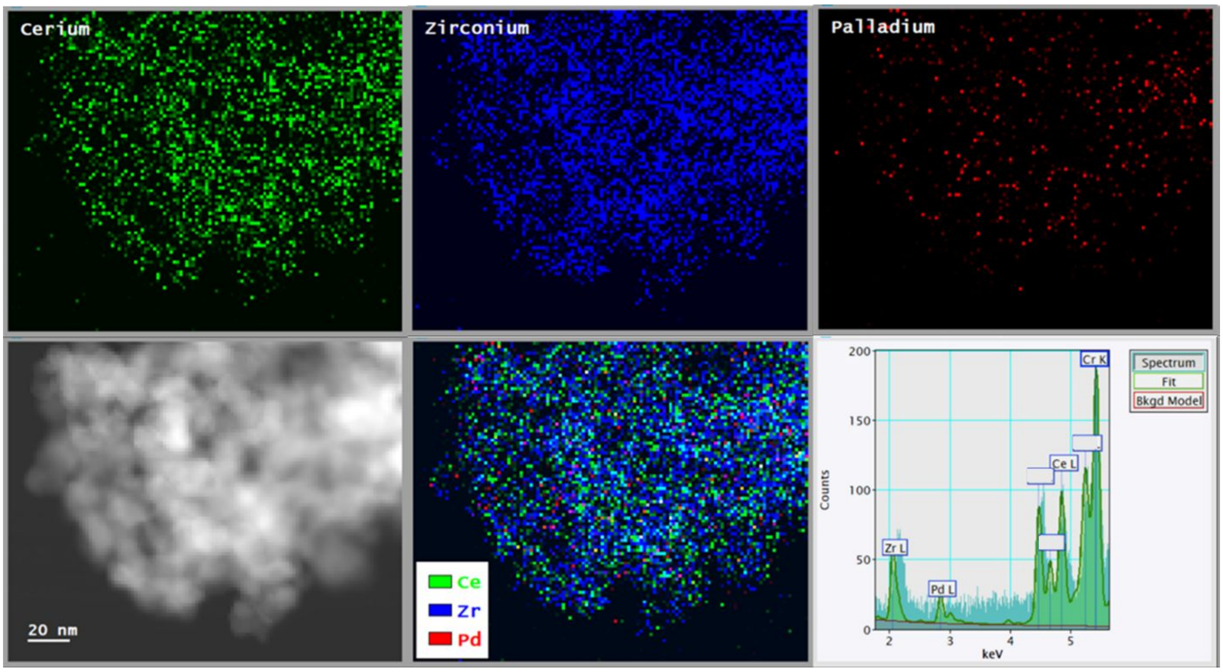

Figure SI16.7 Full elemental maps of Pd/CZ2, fresh, at medium magnification, by TEM coupled with EDS.

The above figure shows a homogeneous distribution of all the elements in the support and of the surface Pd. However, the Pd signal is only slightly above the background noise level. Presumably this region is one where Pd is less concentrated.

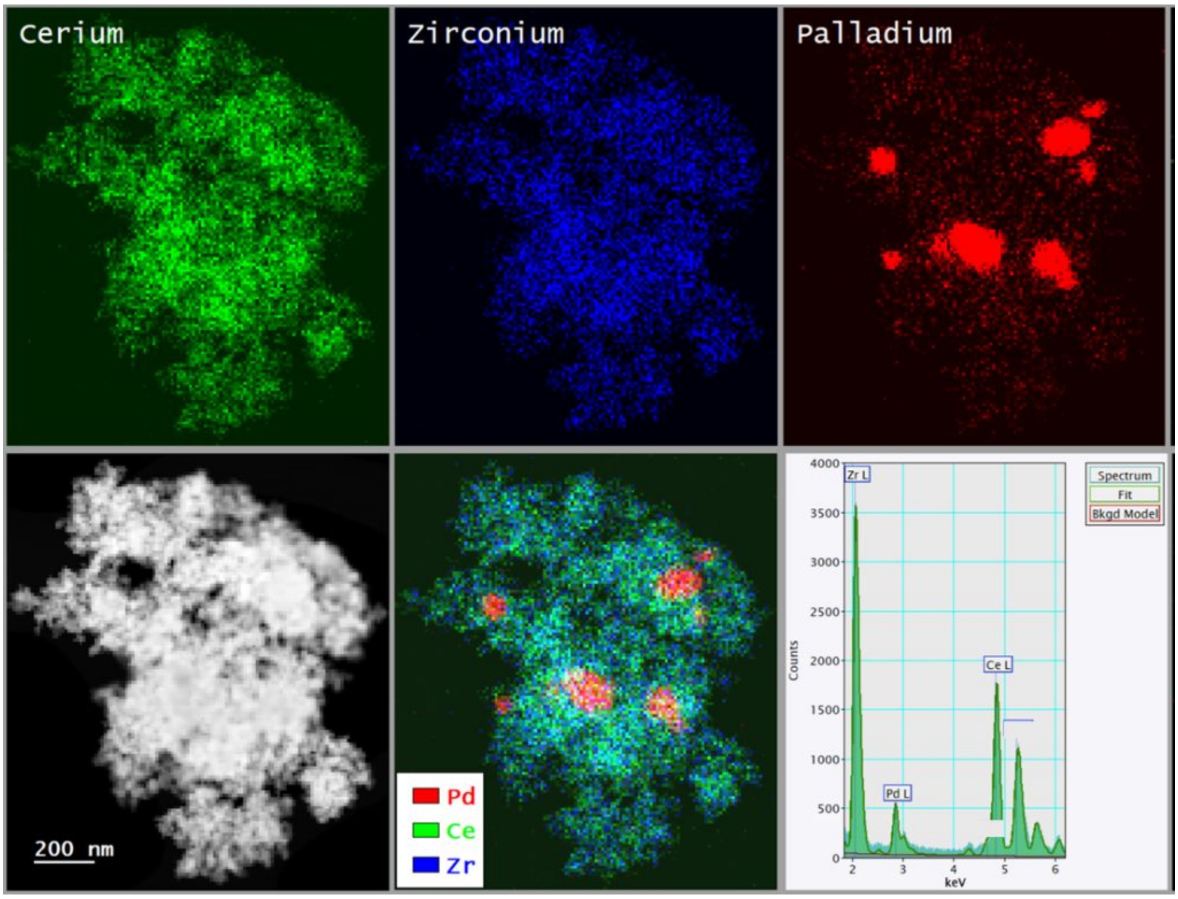

Figure SI16.8. Full elemental maps of Pd/CZ2, aged (950°C, 12h, air), at low magnification, by TEM coupled with EDS.

The above figure shows Pd agglomeration has occurred with areas of high and low Pd concentration. All other elements remain homogeneously dispersed.

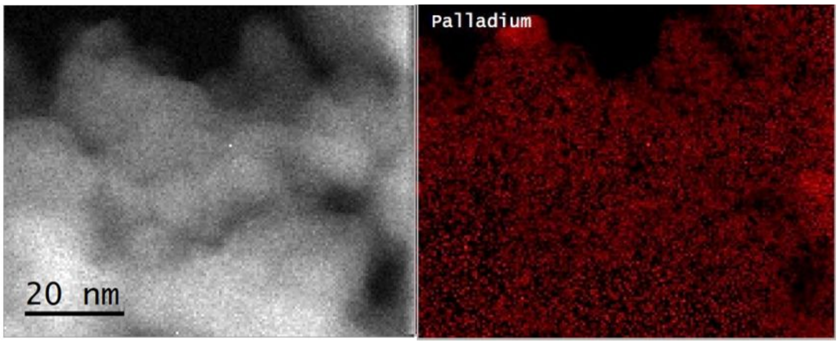

Figure SI16.9. Elemental map of Pd in Pd/CZ2, aged (950°C, 12h, air), by TEM coupled with EELS, as opposed to EDS in earlier figures.

It is worth noting that, using EELS, a background dispersion of Pd in areas other than these high concentration spots is seemingly present.

SI 17 XANES, LCF and EXAFS of Pd/CZ1 and Pd/CZ2, fresh and aged

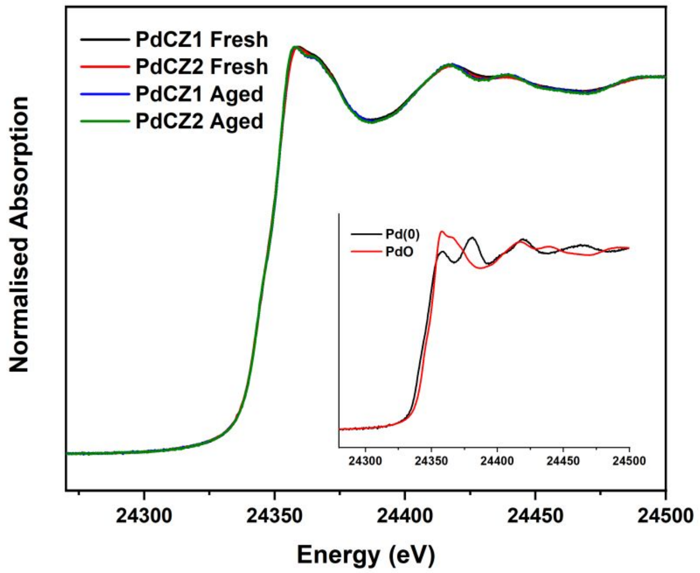

Figure SI17.1. XANES of Pd/CZ1 and Pd/CZ2, fresh and aged at 950 °C for 12 h in air, at the Pd K edge. Measurements were ex situ on B18. References of PdO and Pd^0 (NPs, reduced in H_2 for better comparison) are inserted.

Table SI17.1. LCF fits from the above XANES spectra, using the PdO and Pd^0 references described in figure SI17.1 above.

	**Pd(0) %**	**PdO %**	**Rfac**
**Pd/CZ1 fresh**	0 ± 2	100 ± 2	0.002
**Pd/CZ1 aged**	2 ± 1	95 ± 1	0.001
**Pd/CZ2 fresh**	1 ± 2	99 ± 2	0.003
**Pd/CZ2 aged**	0 ± 1	100 ± 1	0.001

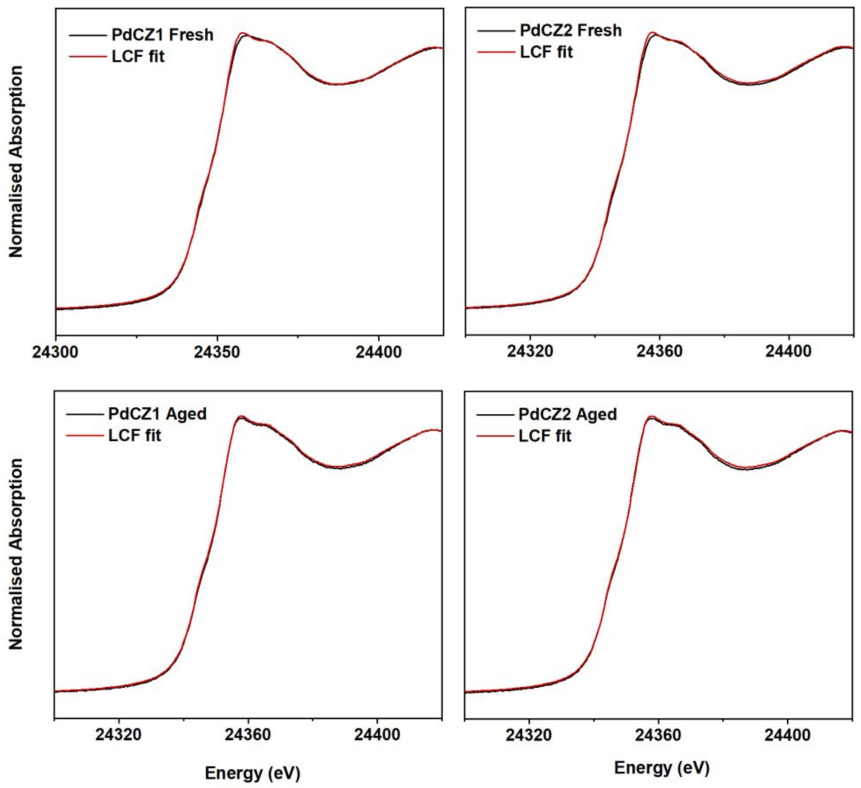

Figure SI17.2. The LCF fits corresponding to the data in the above table (SI12.1)

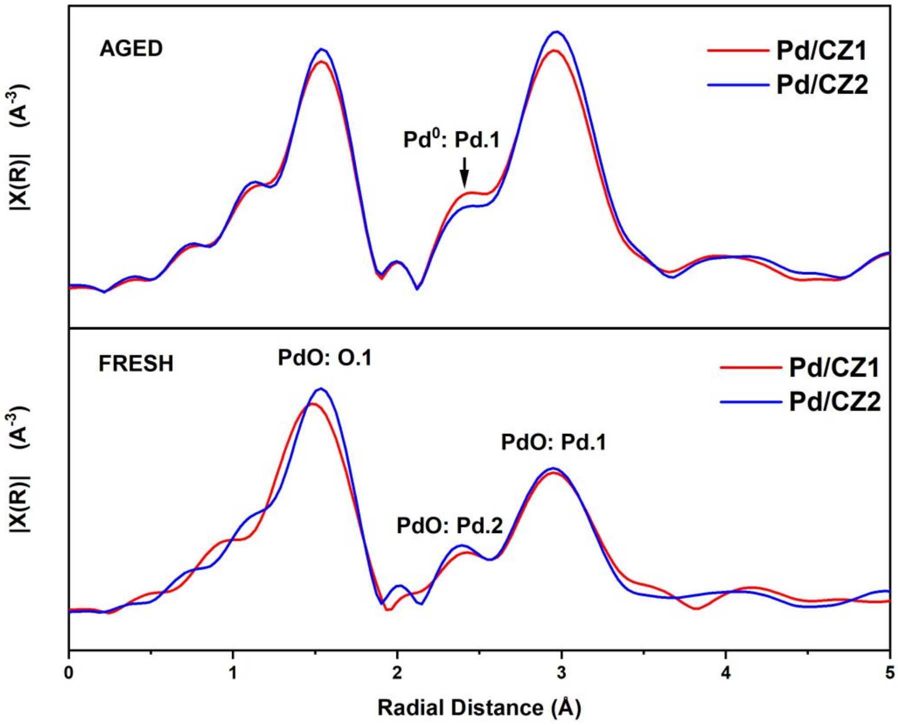

Figure SI17.3. k_2 weighted fourier transform of (bottom) fresh and (top) aged Pd/CZ1 and Pd/CZ2, aged, collected at the Pd K edge on B18. Aging was at 950 °C for 12 h in air.

Tale SI17.2. Fit results of Pd K edge EXAFS using Pd-O and Pd-Pd scattering paths in PdO, and for one sample, a Pd-Pd scattering path in Pd^0. Aging was at 950 °C for 12 h in air.

Sample	R_{fac}	DeltaE (eV)	Path	Coordination number	Bond Length (Å)	σ^2
PdCZ1 Fresh	0.003	0.41 ± 0.63	PdO: O1	3.99 ± 0.10	2.021± 0.005	0.002 ± 0.000
			PdO: Pd1	4.53 ± 0.26	3.055 ± 0.006	0.007 ± 0.000
			PdO: Pd2	5.10 ± 0.40	3.443 ± 0.007	0.007 ± 0.000
PdCZ2 Fresh	0.004	0.42 ± 0.25	PdO: O1	3.94 ± 0.09	2.022 ± 0.002	0.002 ± 0.000
			PdO: Pd1	1.12 ± 0.05	3.048 ± 0.003	0.007 ± 0.000
			PdO: Pd2	0.63 ± 0.04	3.449 ± 0.005	0.007 ± 0.000
PdCZ1 Aged	0.005	2.73 ± 0.32	PdO: O1	3.82 ± 0.15	2.035 ± 0.004	0.002 ± 0.000
			PdO: Pd1	8.01 ± 0.41	3.075 ± 0.004	0.008 ± 0.001
			PdO: Pd2	5.63 ± 0.41	3.456 ± 0.005	0.006 ± 0.001
			Pd^0: Pd1	0.80 ± 0.18	2.760 ± 0.013	0.005 ± 0.002
PdCZ2 Aged	0.007	-0.24 ± 0.78	PdO: O1	4.41 ± 0.17	2.018 ± 0.006	0.002 ± 0.004
			PdO: Pd1	7.84 ± 0.43	3.069 ± 0.006	0.007 ± 0.000
			PdO: Pd2	8.84 ± 0.52	3.442 ± 0.006	0.007 ± 0.000

R=1.1-3.5, k=3.0-12.7, Nipd=14.83. Amplitude was 0.85, refined from foil.

Only the fit of Pd/CZ1 aged is improved by addition of Pd^0: Pd-Pd path

SI 18 Justification of the inference of metal – support interaction

The strength of the metal – support interaction and mobility of the Pd NPs are inferred indirectly in this study from the reduction temperature and rate of PdO, and the efficiency of the re-oxidation of Pd(0). A comparison of the physiochemical properties of Pd/metal oxide catalysts with stronger and weaker metal - support interactions is prudent to explain why this correlation was considered justified. Al_2O_3 is not considered to produce a strong metal-support interaction, whereas ceria and ceria zirconia are.[26] Al_2O_3 as a support for Pd results in lower initial Pd dispersion following impregnation and calcination, with large PdO reflections in the PXRD. The surface area of Al_2O_3 is significantly higher than that of ceria zirconia (170 m^2 g^{-1} compared to 85 m^2 g^{-1}), hence less available surface is not the reason for the higher nucleation of the PdO. linear combination fitting (LCF) of the XANES calculates 11% Pd(0), indicating also an amorphous metallic component to the Pd. The reduction temperature of PdO is lower on Al_2O_3, reported in the literature from an *in situ* XANES under TWC light-off conditions.[27] The reason for strong Pd-Ce interaction which results in the strong metal-support interaction, as opposed to supports such as alumina, was discussed in paragraph 5 of the introduction in the main text.

After thermal aging, the reoxidation of Pd(0) to PdO is far less efficient on Al_2O_3 than on ceria zirconia. Pd(0) reflections are observed in the PXRD (partially overlapped by those of Al_2O_3) and the remaining PdO reflections remain much larger and result from much larger and more crystalline nanoparticles compared to on ceria zirconia (Figure SI18.1). LCF calculates 36% Pd(0) on Al_2O_3 after aging, but 5% or less on ceria zirconia (Table SI18.1).

Finally, even stronger metal-support interaction than observed in this study from other ceria oxide based supports results in the encapsulation of Pd NPs, which drastically inhibits or totally prevents migration.[28]

Taking all of this into consideration, it seems justified to correlate the re-oxidaiton and redispersion of Pd on ceria zirconias to the strength of the metal-support interaction.

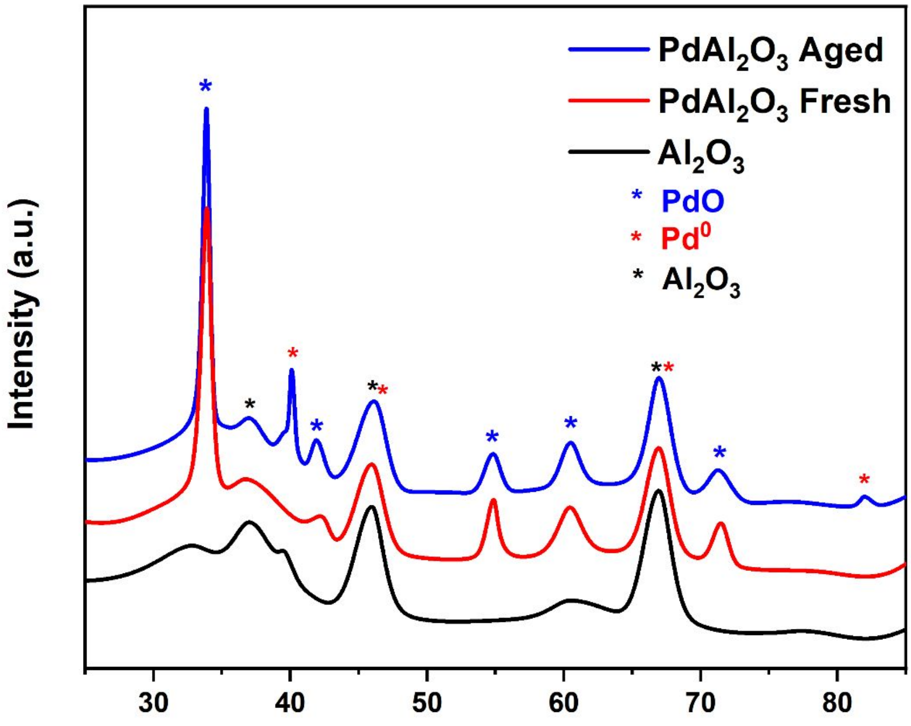

Figure SI18.1 X-ray diffraction pattern of the blank Al_2O_3 support, fresh 5% Pd/Al_2O_3, and aged Pd/Al_2O_3 (950 °C 12 h). PdO reflections are present for the fresh Pd/Al_2O_3, and both Pd0 and PdO for aged Pd/Al_2O_3.

Table SI18.1 Weighted % of Pd^0 and PdO in fresh and aged Pd/CZ1, Pd/CZ2 and Pd/Al_2O_3 determined by linear combination fitting of the Pd K-edge XANES.

	**Pd/CZ1**		**Pd/CZ2**		**Pd/Al_2O_3**	
	**Pd^0 %**	**PdO %**	**Pd^0 %**	**PdO %**	**Pd^0 %**	**PdO %**
Fresh	0	100	1	99	11	89
Aged 950 °C 12h	5	95	0	100	36	64

SI 19 PXRD cycling 650-1050 °C

In situ PXRD temperature cycling at 850 – 650°C and 1050 – 650°C, to complement the 950 – 650°C cycling in the main text. Pd^0 is able to reoxidise on both supports on cooling from the lower temperature of 850°C. Increasing the temperature beyond this, and reoxidation can only occur on CZ2, not CZ1.

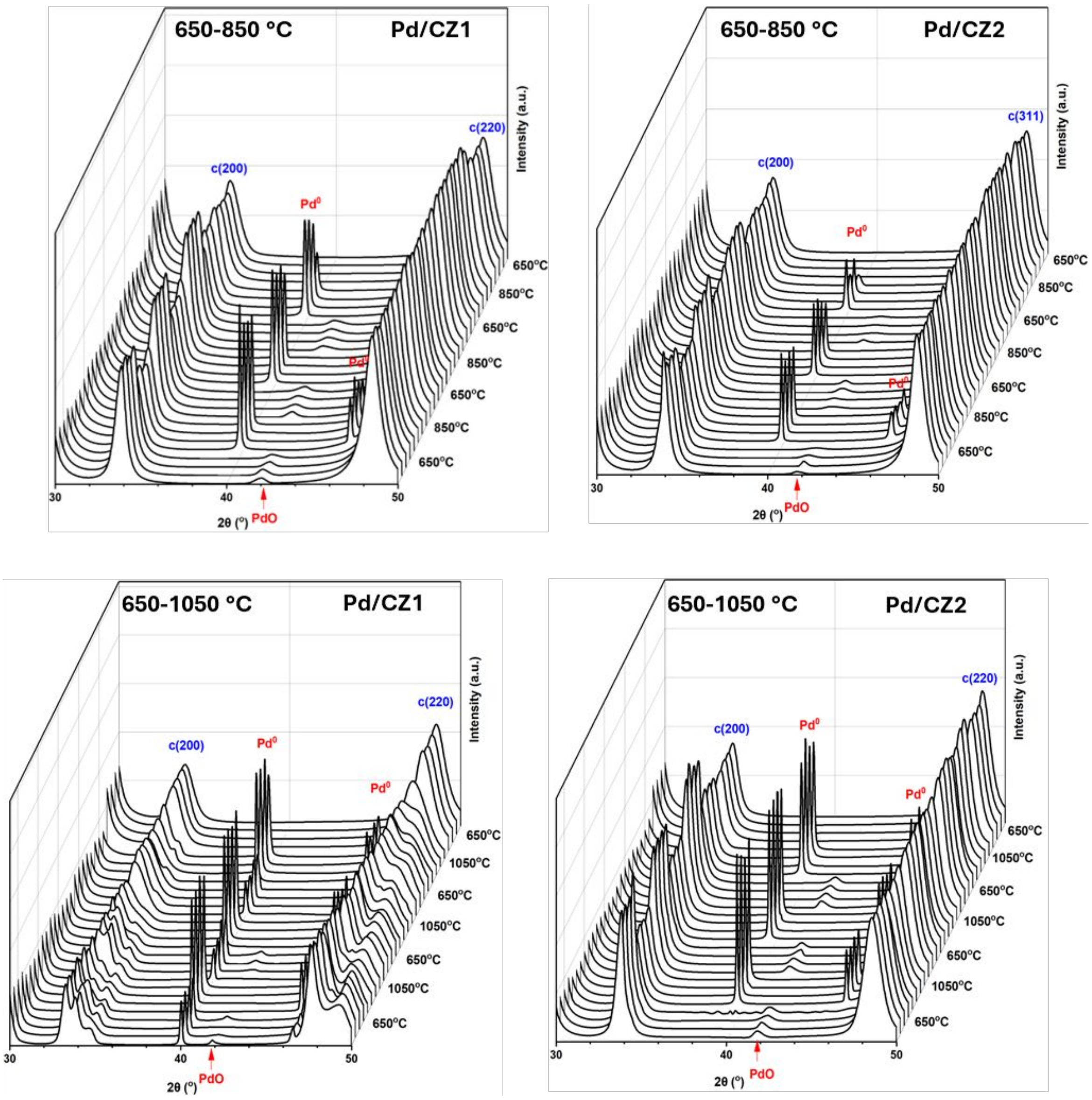

Figure SI19.1. *In situ* XRD of Pd/CZ1 (left) and Pd/CZ2 (right) during cycling, (top) 850-650°C and (bottom) 1050-650°C, in atmospheric air using an Anton Paar chamber. A Savitzky–Golay filter was applied for clarity in the figure.

SI 20 LCF fits of in situ XANES (Figure 9 main text)

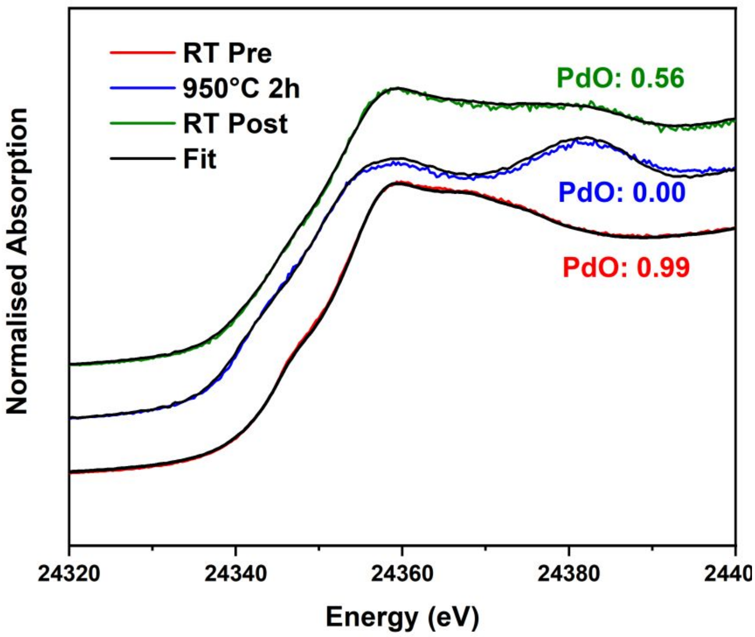

Figure SI20.1. Example linear combination fits, showing high fitting quality, and calculated PdO fraction for Pd/CZ1 at three stages during the described temperature ramp experiment on B18 at DLS.

SI 21 Thermogravimetric analysis of Pd/CZ1 and Pd/CZ2

Standard TGA measurements were performed in atmospheric air. Samples were heated to 800°C at 10°min^{-1}, then immediately to 950°C at 5°min^{-1}, held for 2 h then cooled at the same rates.

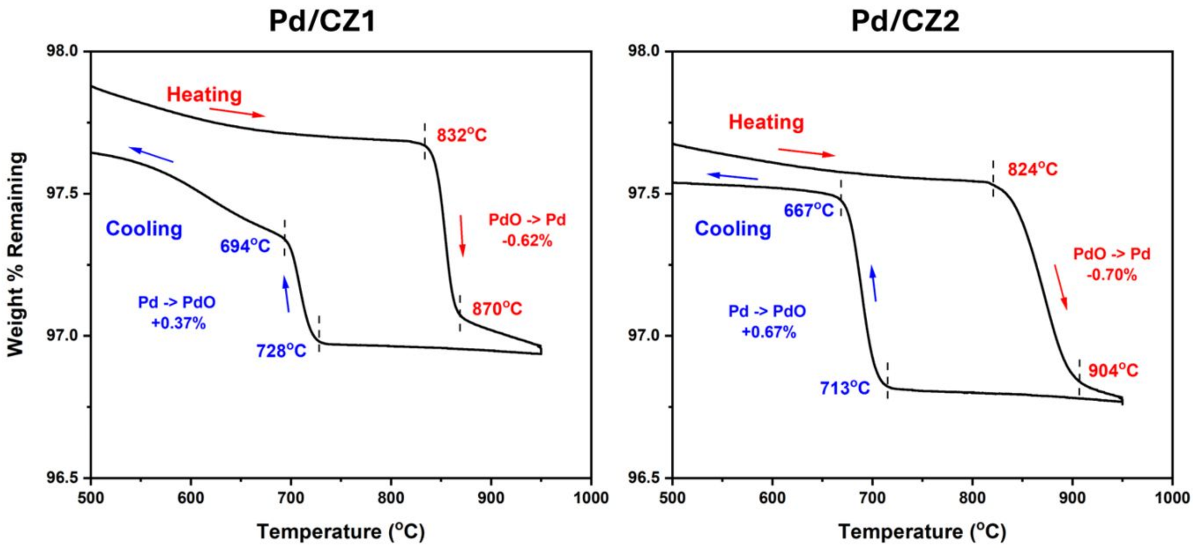

Figure SI21.1. Thermogravimetric analysis of Pd/CZ1 (left) and Pd/CZ2 (right) showing hysteresis loops of % weight loss and gain during a temperature heating and cooling experiment.

The distinctive mass loss of around 0.7 % for both samples corresponds to the decomposition of PdO to Pd0. It occurs at a slightly lower temperature than observed by XRD, likely due to the crystallinity requirement for XRD visibility or thermal miscalibration. On cooling, the mass remains constant until around 720 °C. Pd/CZ2 regains all its oxygen in one linear process, whereas Pd/CZ2 appears to be split into two distinct stages, indicating two separate reoxidation stages. The samples were held at 950 °C for 2 h before cooling, to imitate the sintering which occurred during in situ XRD and XANES experiments. Less total mass was regained by Pd/CZ1, again corroborating the *in situ* experiments which showed less total Pd^0 reoxidation on CZ1.

SI 22 results from combined XRD and XAS experiment

The purpose of this combined experiment was to validate the results of the individual in situ experiments presented in the main text, and find whether the changes in the crystalline phases were occurring simultaneously to speciation changes. This was especially important given the temperature sensitivity of the autoreduction of PdO, and subsequent reoxidation.

During the ramp, 5 discreet energies along the XANES spectra (each an average of two points) were measured. The spectra of both PdO and Pd(0) with the selected energies marked are given in Figure SI21.1. One energy point was below the edge to allow any significant changes to I0 to be observed. Note the 5 absorption plots were normalised independently, and different scales are selected for each to best demonstrate the temperature at which the most significant changes occurred.

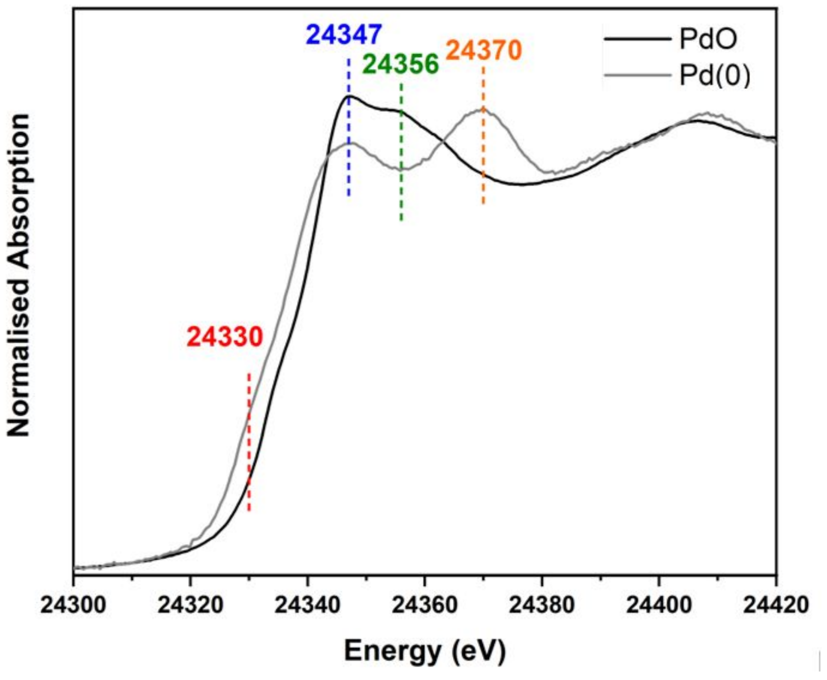

Figure SI22.1. Reference Pd^0 and PdO spectra overlayed with 4 of the discreet energies at which fluoresced intensity was measured. The fifth energy selected was on the baseline before the absorption edge at 24250 eV.

The decomposition of PdO to Pd^0 for Pd/CZ1 during the temperature ramp is shown by temperature resolved diffraction and absorption spectroscopy in Figure SI22.2 below. The decomposition onset is indicated by the emergence of Pd^0 reflections in the PXRD, and by an inflection in the intensity lines of the absorption plot. These occurred simultaneously at 852 – 857 °C for Pd/CZ1. Identical behaviour was observed for Pd/CZ2, though the decomposition began at 770°C. This discrepancy was not observed by TGA, where decomposition temperatures were within 20°C, and may be due to different capillary placement, or temperature spikes during the ramp causing early reduction. If Pd/CZ2 did experience higher temperatures than Pd/CZ1, then its enhanced Pd reoxidation behaviour is yet more impressive.

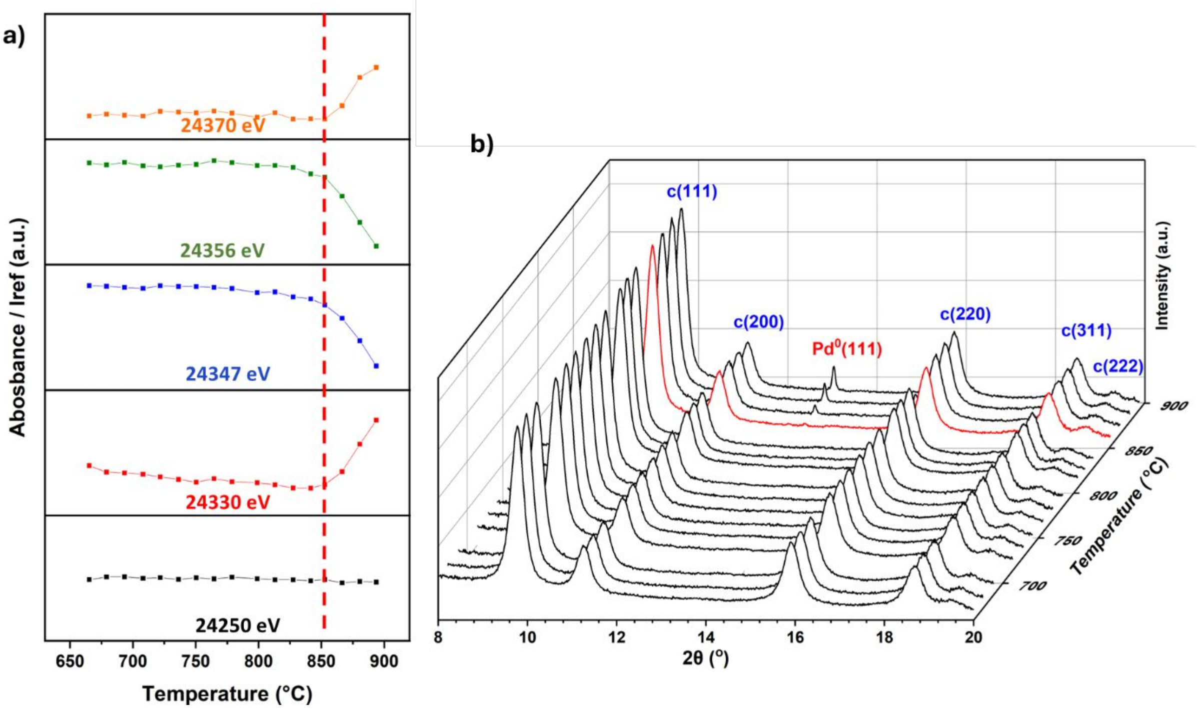

Figure SI22.2. Intensity of **a)** fluoresced X-rays and **b)** PXRD patterns collected during a temperature ramp of Pd/CZ1. The onset of PdO decomposition is indicated in red on each figure. Absorption was at the Pd K edge at 24.3 keV, and diffraction measurements at 24 keV.

The cooling stage was monitored in the same way, see Figure SI22.3, and here the differences between Pd/CZ1 and Pd/CZ2 are more pronounced. For Pd/CZ1, the reoxidation was more

gradual than the reduction, beginning just above 600 °C and eventually plateauing at around 400 °C, observed by both techniques. However, no PdO reflection formed during the reoxidation. PdO reflections were observed by lab in situ temperature cycling study for Pd/CZ1, however only after a couple of sequential cycles, whereas the temperature programme here corresponds to only one temperature cycle. In contrast, for Pd/CZ2, Reoxidation began at 865 °C and oxidation state and reflection changes were finished by 620 °C. PdO reflections are present immediately upon the loss of Pd^0.

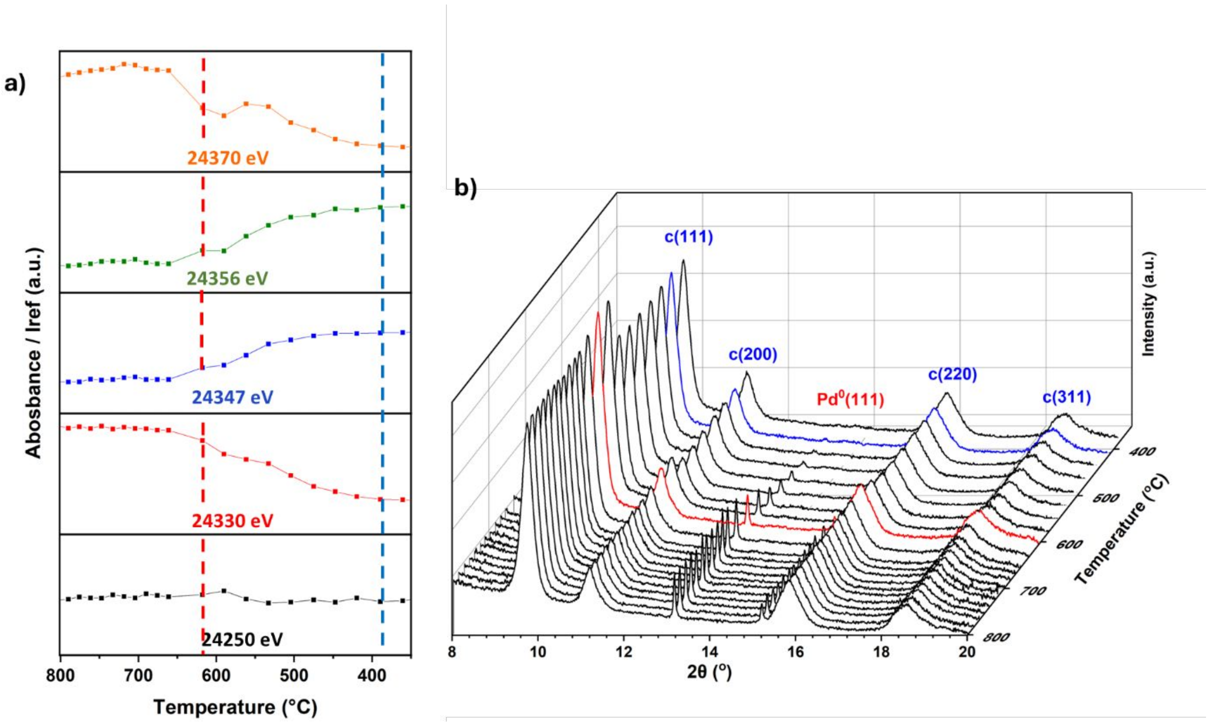

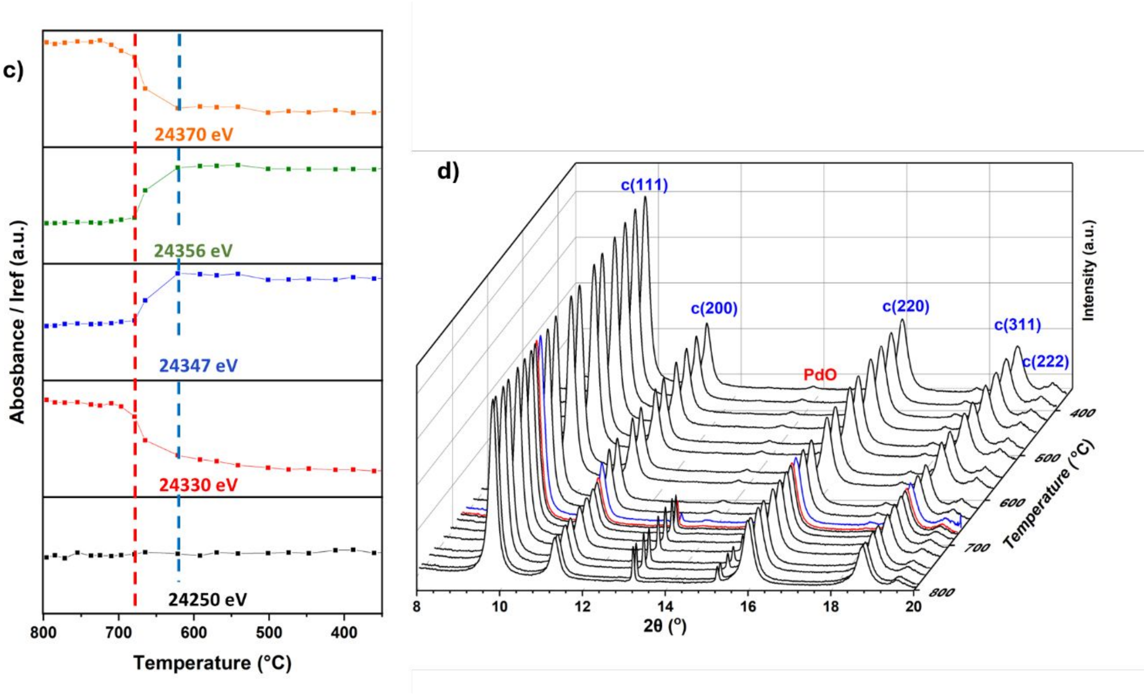

Figure SI22.3. Intensity of fluoresced X-rays and PXRD patterns collected during cooling of **a,b)** Pd/CZ1 and **c,d)** Pd/CZ2. The onset, and end of, the Pd^0 reoxidation process are indicated in red and blue respectively. Absorption measurements were at the Pd K edge at 24.3 keV, and diffraction measurements at 24 keV. In the PXRD of Pd/CZ1, figure c), The loss of intensity of the ceria zirconia reflections, observable at ~600 °C, are likely due to the sample shifting in the capillary during cooling.

The oxidation states of the Pd were compared during the 650 °C isothermal dwell during cooling, and once fully cooled, by LCF. Complete XANES scans were measured at these points, rather than the discreet energy measurements. Whilst these calculated oxidation states have a higher error value than would be desired due to sample shifting during cooling, they clearly indicate the more rapid reoxidation of Pd^0 on CZ2 compared to CZ1, and are consistent with TGA results in SI 21 above.

Table SI22.1. Linear combination fit result of Pd/CZ1 and Pd/CZ2 during in situ thermal experiment at the Pd K edge. Samples were cooled from 900 °C, pausing at 650 °C for XANES measurements, and then cooled fully and measured again.

	Pd^0 %	
	**Pd/CZ1**	**Pd/CZ2**
**650 °C cooling**	94 ± 2	55 ± 5
**RT cooled**	30 ± 10	33 ± 7

Interestingly, the LCF shows both samples contain equal quantities of PdO, however PdO reflections are only present in the diffraction pattern of Pd/CZ2 (Figure SI 22.2 compared with Figure SI 22.3 above). This indicates the reoxidised nanoparticles are more crystalline on Pd/CZ2.

SI 23 Long duration experiment: data, refinements and plotted parameters

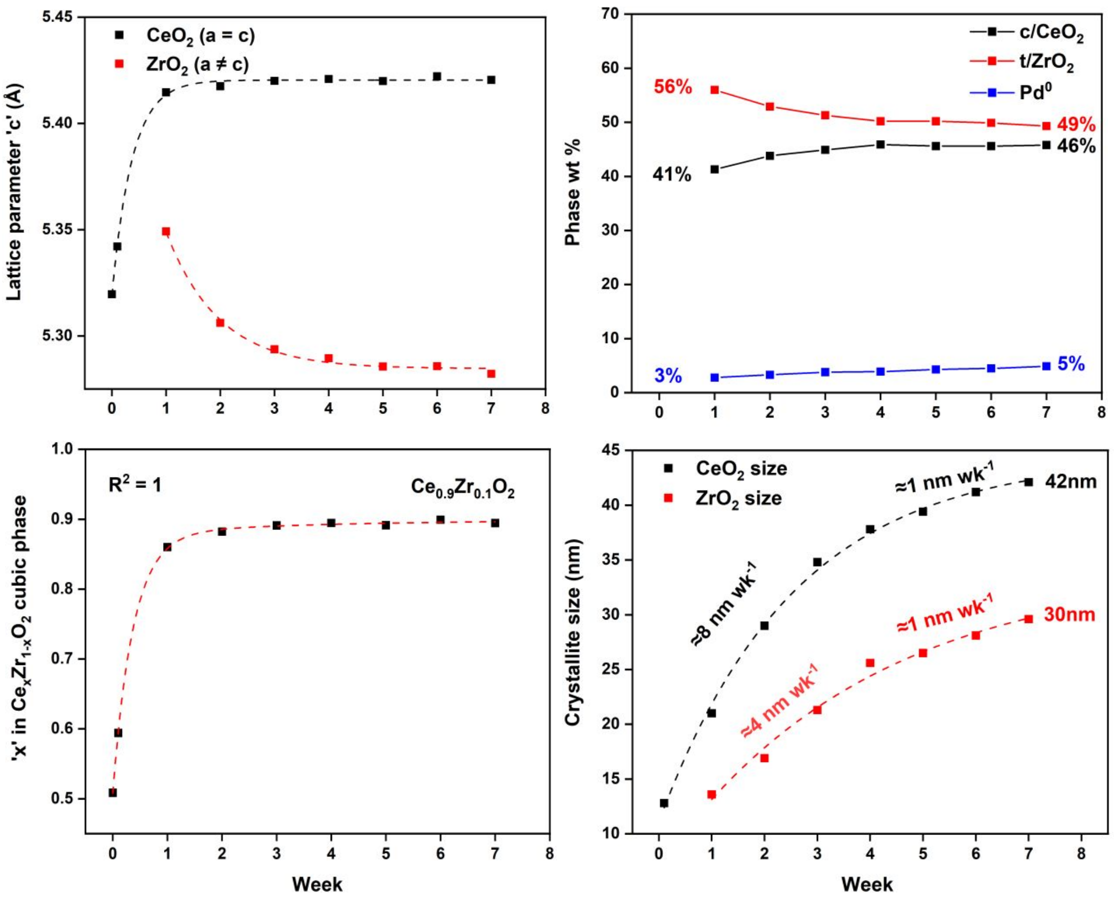

Figure SI23.1. The calculated **a)** lattice parameters, **b)** crystalline phase wt %, **c)** stoichiometry of the ceria rich cubic phase and **d)** crystallite size, all refined from the measurements of Pd/CZ1. At week 0 only the cubic phase was fit, and from week 1 of the separated cubic (CeO_2) and tetragonal (ZrO_2) phases (only the 'c' value for ZrO_2 is shown). The data point at 6 h comes from a complementary lab based in situ experiment.

Fit rational and details for Pd/CZ1:

Prior to separation, the mixed phase was fit to a $Ce_{0.5}Zr_{0.5}O_2$ $Fm\overline{3}m$ phase, and after separation, to pure CeO_2 ($Fm\overline{3}m$) and pure ZrO_2 (P42/nmc). High levels of disorder and evolving compositions made it impossible to fit the Ce:Zr ratios of each phase with accuracy, hence pure phases were fit and the lattice parameters used to monitor the stoichiometry. A Pd^0 $Fm\overline{3}m$ phase was also fit. Background was fit using a Chebyschev-1 function with two background peaks fitted to account for contributions from the quartz window. The Uiso of both Ce^{4+} and Zr^{4+} were made equivalent in the mixed phase during week 0 at 0.0417. After phase separation began, the Uiso values of the Ce^{4+} and O in CeO2 were 0.0417 and 0.06 respectively, and for Zr^{4+} and O^{2-} in the ZrO2 phase were 0.051 and 0.1 respectively. For the Pd^0 phase, strain was not refined and the Uiso was fixed at 0.01, Crystallite sizes are therefore included for reference to show overall phase growth, and replaced by '>max' when sizes surpass 1000 nm. Goodness of fit is given by the weight of residuals, wR (%).

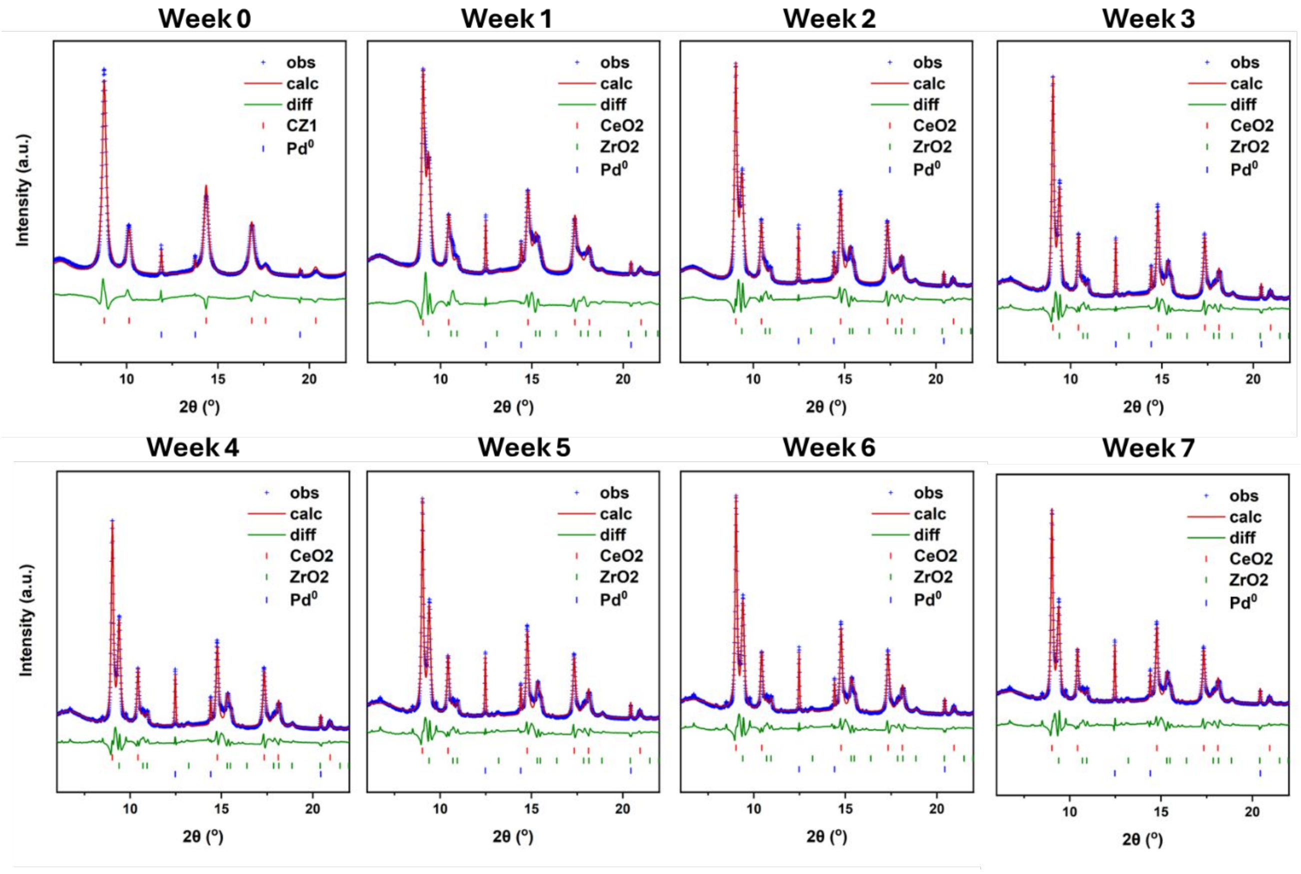

Figure SI23.2 Data, calculated fit and difference plot of Pd/CZ1 collected in situ over 8 weeks at 850 °C, measured in transmission at 25 keV. The low intensity reflections which appear from week 3 primarily at low angles were found to be due to the ceramic sample holder ring. The phases of (initially) the mixed $CeZrO_2$, then of the CeO_2 rich $Fm\bar{3}m$ and ZrO_2 rich P42/nmc phases are indexed, along with that of $Fm\bar{3}m$ Pd^{0}. Week by week stoichiometries and phase fractions were described in Figure SI23.1 above. The lattice size data recorded after 0.6h at 950C was from a complementary laboratory experiment, though crystallite sizes were not calculated given the difference in instrument.

Table SI23.1. Fit results from Rietveld refinement of Pd/CZ1 over 7 weeks (8 measurements) at 950°C, all measurements taken in situ at 950°C.

**Time (wk)**	**Lattice parameters (Å)**				**Crystallite size (nm)**			**Phase wt%**			**wR (%)**
	**CeO_2 'a'**	**ZrO_2 'a'**	**ZrO_2 'c'**	**Pd^0 'a'**	**CeO_2**	**ZrO_2**	**Pd^0**	**CeO_2**	**ZrO_2**	**Pd^0**	
**0**	5.332	/	/	3.929	13	/	78	97.0	/	3.0	5.4
**0.6h**	5.342	/	/	3.927	-	-	-	-	-	-	-
**1**	5.415	3.670	5.349	3.930	21	14	520	20.4	77.4	2.2	5.1
**2**	5.418	3.668	5.306	3.929	29	17	589	22.3	75.0	2.7	5.8
**3**	5.420	3.662	5.294	3.929	35	21	N/A	23.1	73.8	3.1	6.3
**4**	5.421	3.659	5.290	3.929	38	26	N/A	23.8	72.9	3.3	5.8
**5**	5.420	3.657	5.286	3.928	39	27	N/A	23.7	72.8	3.6	5.7
**6**	5.422	3.657	5.286	3.929	41	28	N/A	23.7	72.5	3.8	8.7
**7**	5.420	3.655	5.282	3.929	42	30	N/A	23.9	71.9	4.1	4.3

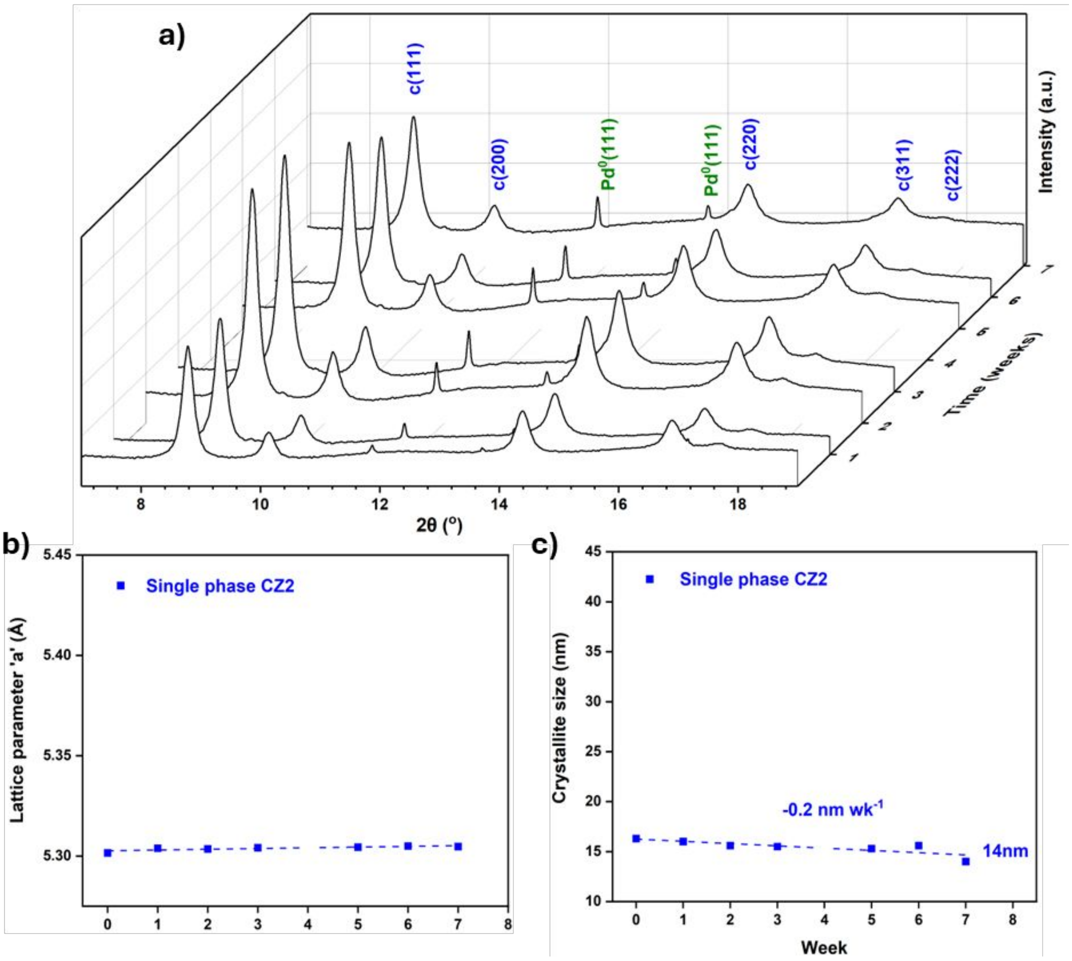

Figure SI23.3. a) Stacked in situ XRD patterns of Pd/CZ2 collected at 850 °C at the long duration facility on I11. Samples were housed in Linkam cells and held at temperature for 8 weeks (labelled 0-7). The reflections corresponding to the cubic Fm$\overline{3}m$ ceria zirconia phase are labelled 'c', and the Fm$\overline{3}m$ Pd^0 reflections are also indexed. **b)** Lattice parameter and **c)** crystallite size of the 'single phase' Pd/CZ2, using the same y axis scale as used for Pd/CZ1 in the main text. Note data for week 4 is unfortunately missing.

Fit rational and details for Pd/CZ2:

The two phases that were fit were a $Ce_{0.36}Zr_{0.59}Re_{0.05}O_{2-x}$ Fm3$\overline{m}$ phase, shortened to 'CZ' in the table below, and a Pd^0 Fm3$\overline{m}$ phase. The lattice parameters of the ceria zirconia phase did not change sufficiently to warrant refining of the phase stoichiometry, given the additional error incurred by this process. Background was fit using a Chebyschev^{-1} function with two

background peaks fitted to account for contributions from the quartz window. The Uiso of all cations in the ceria zirconia phase were made equivalent. For the Pd^0 phase, strain was not refined and the Uiso was fixed at 0.01, as it was deemed highly unlikely that the crystallite size calculations would be accurate for such large crystallites using the Scherrer equation. Again, crystallite sizes are therefore included to demonstrate overall crystallite growth, and to allow for a good overall fit, but are not expected to be accurate. Goodness of fit is given by the weight of residuals, wR (%).

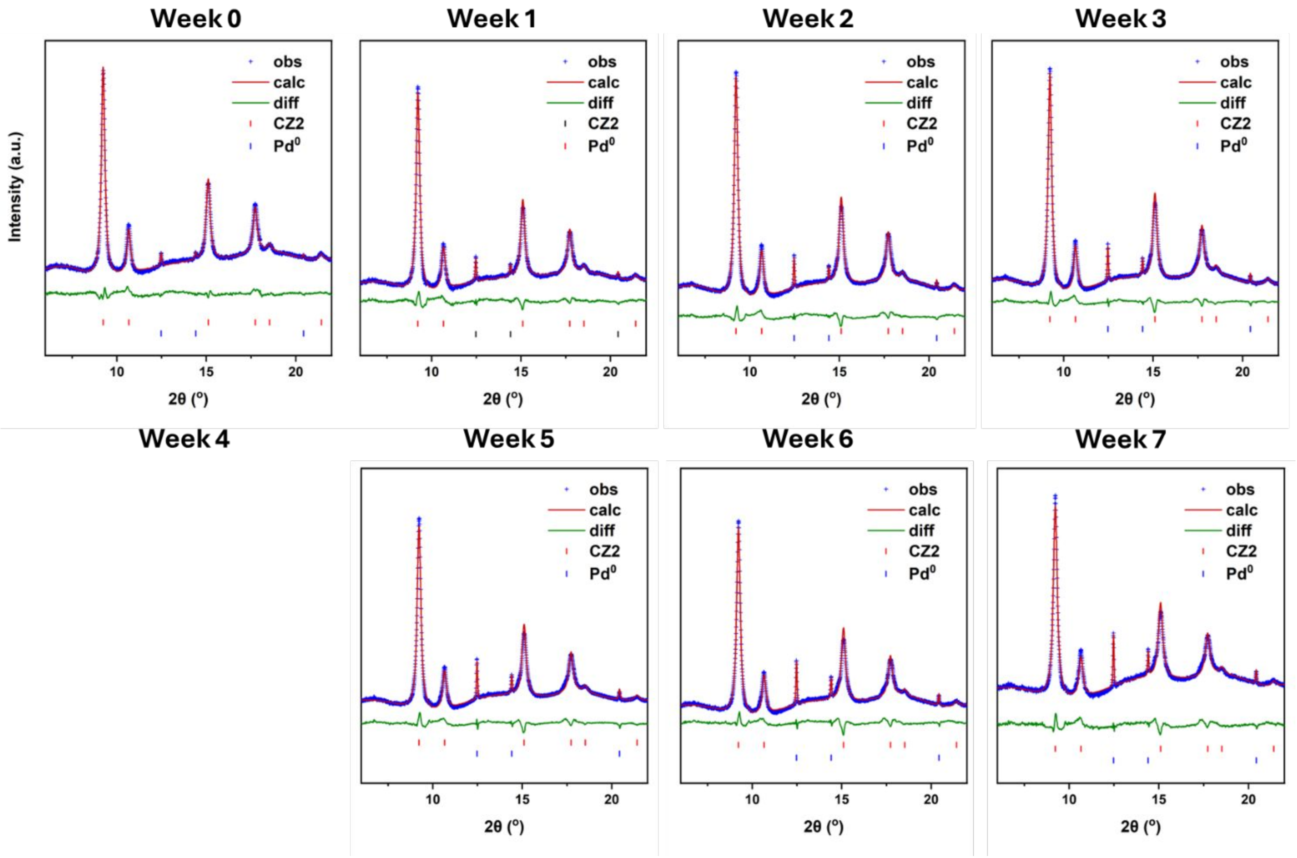

Figure SI23.4. Data, calculated fit and difference plot of Pd/CZ2 collected in situ over 8 weeks at 850 °C, measured in transmission at 25 keV. Note data for week 4 is unfortunately missing. The higher background of this data is a result of a quartz, not diamond, window in the Linkam cell. The phases of the mixed RE doped CeZrO2 ($Fm\bar{3}m$) and the Pd^0 ($Fm\bar{3}m$) are indexed. The stoichiometry of the ceria zirconia phase did not change over the 8 weeks, described below.

Table SI23.2. Fit results from Rietveld refinement of Pd/CZ2 over 7 weeks (8 measurements) at 950°C, all measurements taken in situ at 950°C.

Time (wk)	Lattice parameters (Å)		Crystallite size (nm)		Uiso (cation)	Phase wt%		Strain (a.u.)	wR (%)
	CZ	Pd^0	CZ	Pd^0	CZ	CZ	Pd^0	CZ	
**0**	5.310	3.926	13	71	0.0569	98.7	2.0	6734	1.1
**1**	5.304	3.930	16	100		98.0	2.0	8690	1.5
**2**	5.304	3.929	16	168	0.0673	98.0	2.3	8690	1.9
**3**	5.304	3.930	16	275	0.0673	97.7		8690	2.0
**4**									
**5**	5.304	3.931	15.	234	0.0673	97.2	2.8	8690	1.8
**6**	5.305	3.930	16	339	0.0673	97.0	3.0	8690	1.9
**7**	5.305	3.930	14.	564	0.0673	96.9	3.1	8690	1.4

SI 24 Oxygen Storage Capacity

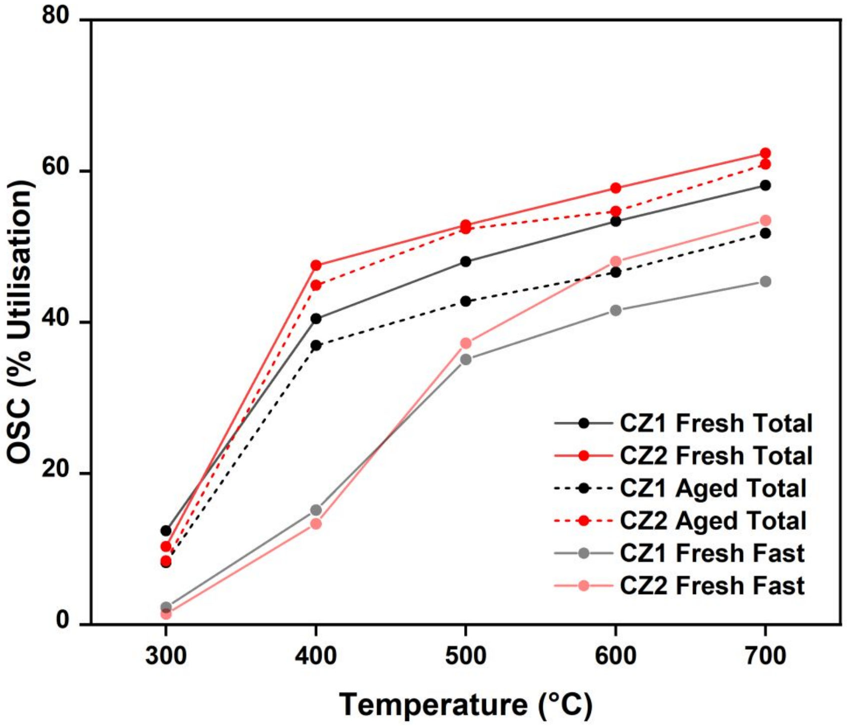

Figure SI24.1. Oxygen storage capacity in % utilisation of CZ1 and CZ2, fresh and aged (950 °C, 12h). For the fresh samples, both the fast and total OSC are given, calculated from the quantity of CO oxidised over 3.5s and 300s respectively.

Values for the total OSC of the fresh samples are reported below as umolCOg^{-1} and % Ce utilisation for comparison.

Table SI24.1. Dynamic OSC values of CZ1 and CZ2, calculated from total CO oxidised over 300s at each temperature. The % utilisation is calculated from the stoichiometries of each support.

**Temperature (°C)**	**OSC (µmol/g)**		**% Ce utilisation**	
	**CZ1**	**CZ2**	**CZ1**	**CZ2**
**300**	210	135	12	10
**400**	685	625	40	48
**500**	812	695	48	53
**600**	903	759	53	58
**700**	984	820	58	62

SI 25 HC conversion, N_2 selectivity and selectivity to various nitrogen containing products during TWC testing

As the experiments were transient with a net reducing gas composition ($\lambda = 0.99$), the conversions oscillate each 3 seconds, and therefore some never quite reach 100% for even the most active catalyst.

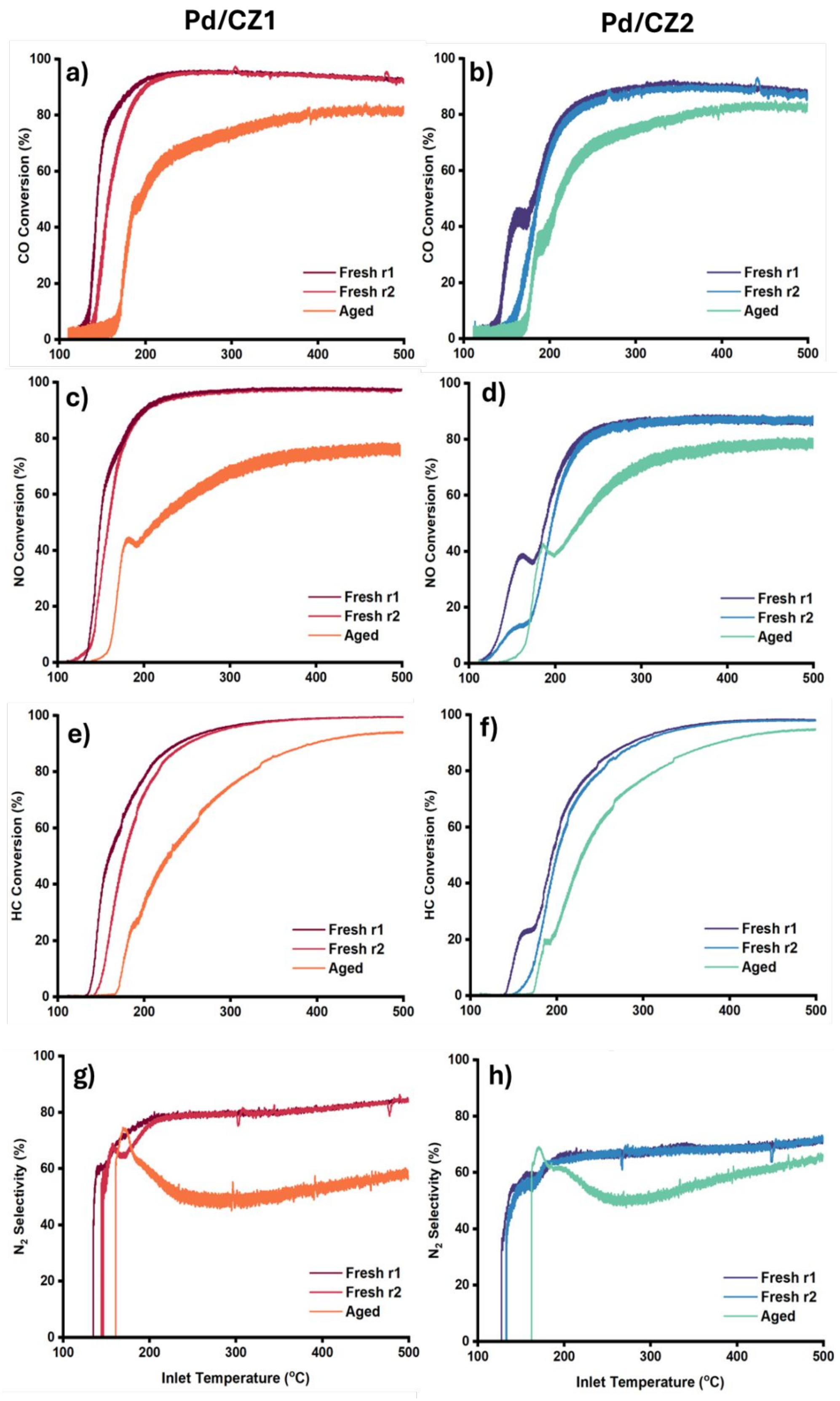

Figure SI25.1 a-b) CO, **c-d)** NO, **e-f)** total hydrocarbon (HC) conversion and **g-h)** total N_2 selectivity of (left) Pd/CZ1 and (right) Pd/CZ2 fresh, from TWC activity testing in model

exhaust gas with 5% water. Data is from the first temperature ramp (Fresh r1), and second temperature ramp (Fresh r2) (performed directly after cooling of r1), and after thermal aging in air at 950 °C for 12 h (aged)

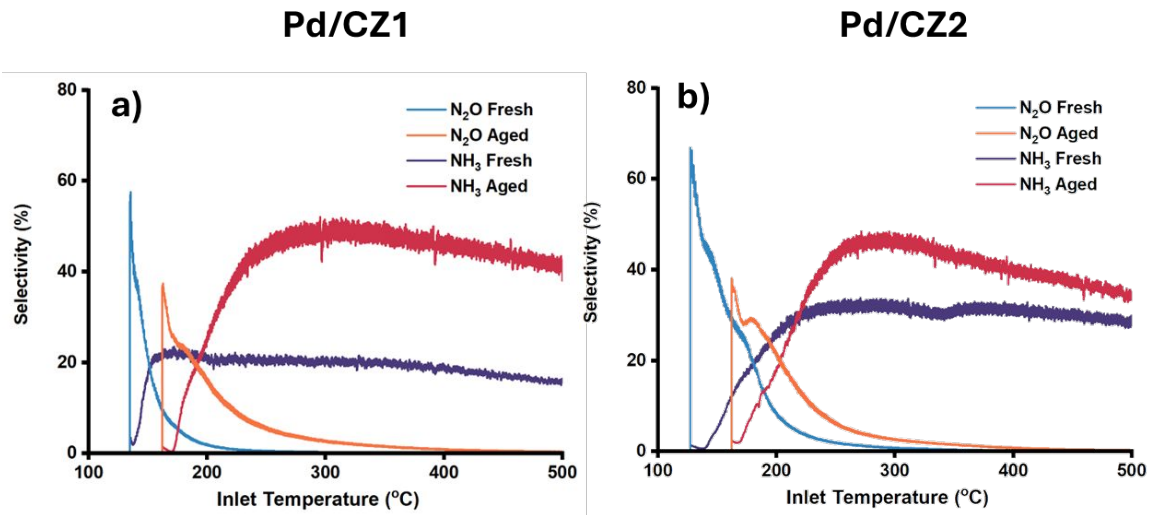

Figure SI25.2. NH_3 and N_2O selectivities from NO conversion by **a)** Pd/CZ1 and **b)** Pd/CZ2 fresh and aged (950 °C, 12h) from TWC activity testing. Selectivity of both species was calculated from outlet concentrations by FTIR, as described in the section 2.7 of the main text.

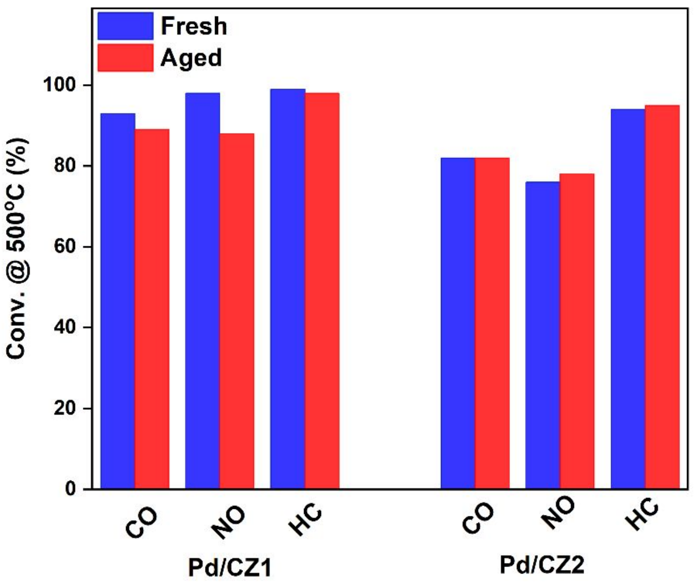

Figure SI 25.3. The conversion calculated at 500°C of CO, NO, and HC (total hydrocarbons) for Pd/CZ1 and Pd/CZ2 fresh and aged (950°C, 12 h, in air). Experiments were under model exhaust conditions.

SI 26 Performance and PXRD results of Pt/CZ1 and Pt/CZ2, before and after thermal aging.

5 wt % platinum on ceria zirconia (both CZ1 and CZ2) catalysts were prepared and aged identically to the Pd catalysts described in the main text. During synthesis, the only difference was the use of $Pt(NO_3)_2.xH_2O$ rather than the Pd equivalent. The performance data and PXRD of the catalysts after aging is presented here for comparison with Pd results in the main text and in SI section 25.

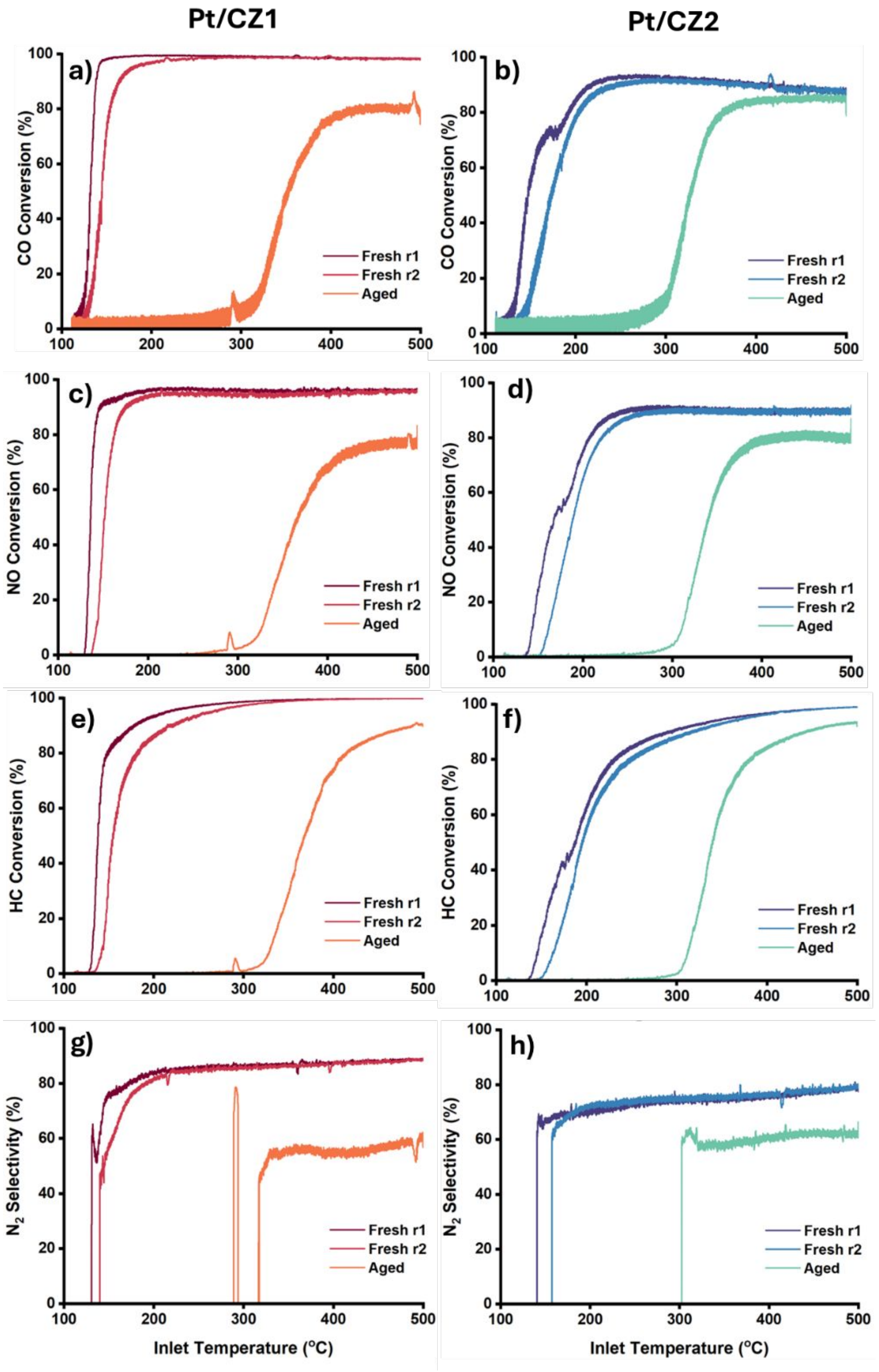

Figure SI26.1 a-b) CO, **c-d)** NO, **e-f)** total hydrocarbon (HC) conversion and **g-h)** total N_2 selectivity of (left) Pt/CZ1 and (right) Pt/CZ2 fresh, from TWC activity testing in model exhaust gas with 5% water. Data is from the first temperature ramp (Fresh r1), and second temperature ramp (Fresh r2) (performed directly after cooling of r1), and after thermal aging in air at 950 °C for 12 h (aged). Note that as N_2 was only calculated above 5 % NO conversion, there is a blip in g) where NO conversion temporarily surpassed 5 %.

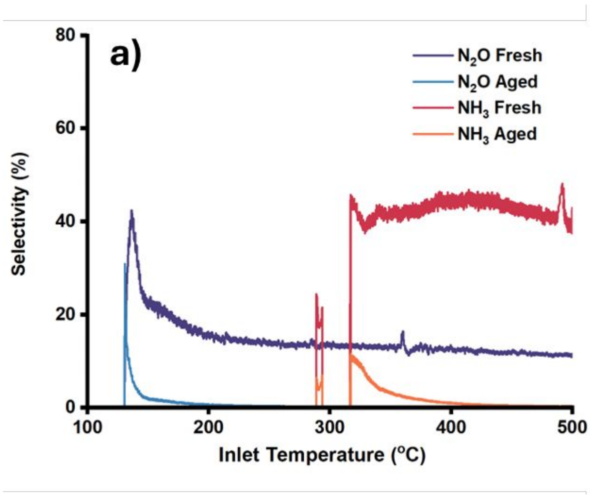

Figure SI26.2 NH_3 and N_2O selectivities from NO conversion by **a)** Pt/CZ1 and **b)** Pt/CZ2 fresh and aged (950 °C, 12h) from TWC activity testing. Selectivity of both species was calculated from outlet concentrations by FTIR, as described in the section 2.7 of the main text. Note that as selectivities to nitrogen containing products was only calculated above 5 % NO conversion, there is a blip in Pt/CZ1 data where NO conversion temporarily surpassed 5 %.

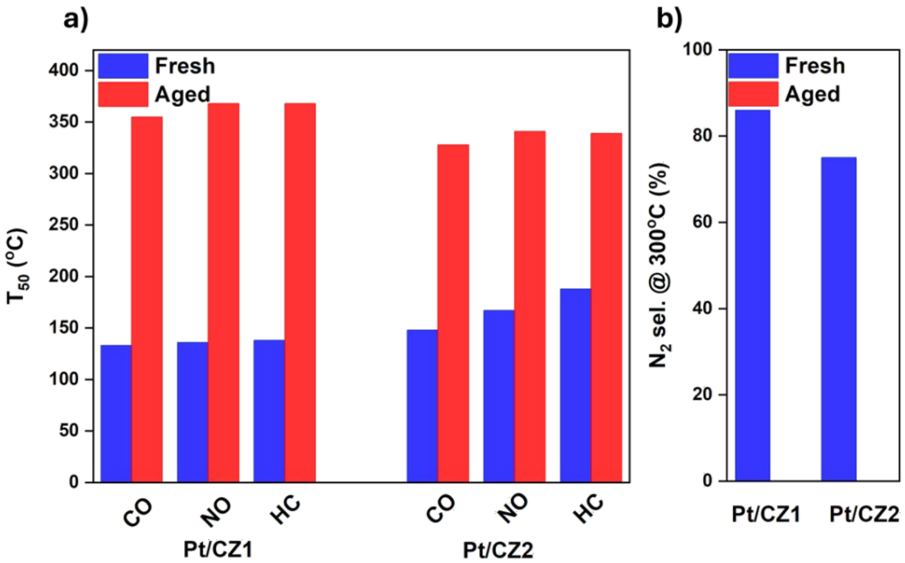

Figure SI26.3 a) T50 values (temperature at 50% conversion) for the conversion of CO, NO, and HC (total hydrocarbons), along with **b)** the N2 selectivity at 300°C, for Pt/CZ1 and Pt/CZ2 fresh and aged (950°C, 12 h, in air). Experiments were under model exhaust conditions. Note that as NO conversion was below 5% at 300°C, no N2 selectivity is recorded.

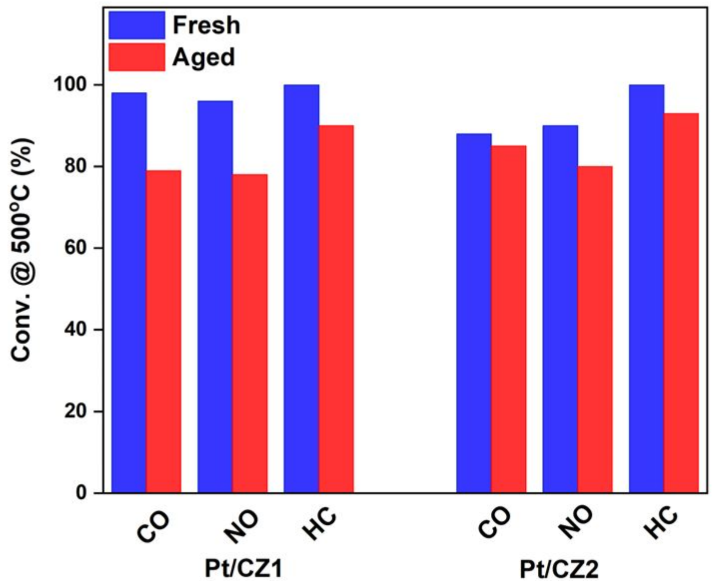

Figure SI26.4 The conversion calculated at 500°C of CO, NO, and HC (total hydrocarbons) for Pt/CZ1 and Pt/CZ2 fresh and aged (950°C, 12 h, in air). Experiments were under model exhaust conditions.

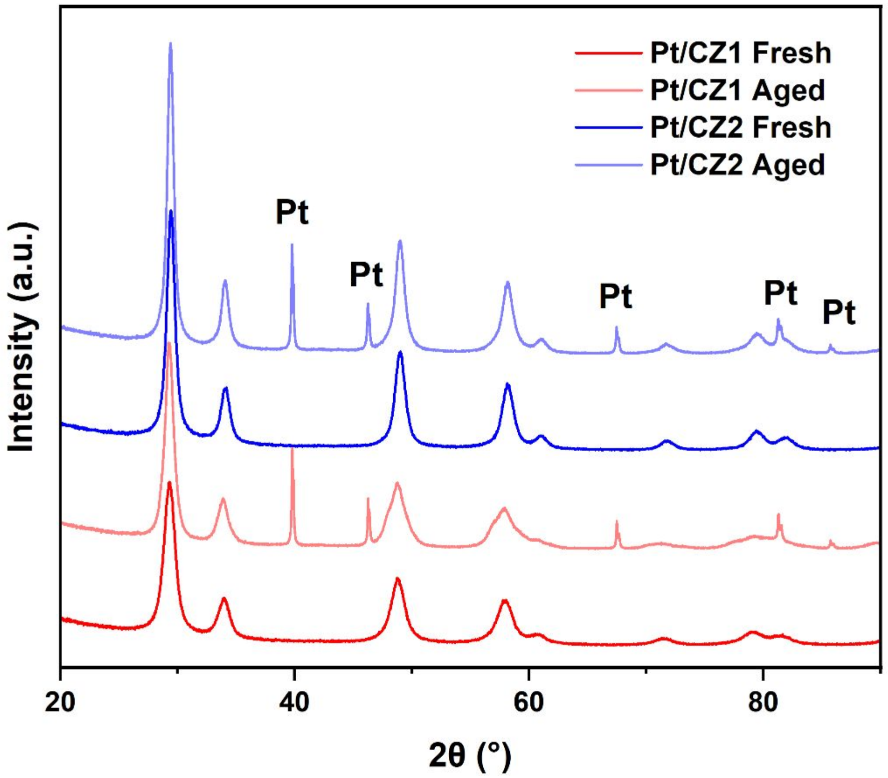

Figure SI26.5 Stacked room-temperature PXRD patterns of Pt/CZ1 (red) and Pd/CZ2 (blue), fresh (bold) and aged at 950°C for 12 h (faded). This diffractomer was not equipped with a monochromator, resulting in slight splitting of Pt reflections observed at high angles.

A brief comparison of Pt and Pd

It must first be noted that the comparison of Pd with Pt on ceria zirconia for TWC application has been studied and clearly by Wang and Zhao and by Vlachou et al.[29,30] Another study by Cooper and Beecham compares combinations of Pt + Rh with Pd + Rh, reporting both vehicle test results and bench engine test results, however the influence of Rh makes this study less effective for direct comparison of Pd and Pt.[31] Theis also reported Pt/CZ with Pd/CZ for NOx adsorption, and the responsible properties for this reaction are considered relevant to those which affect TWC performance.[32] Overall findings here regarding the differences in behaviour of Pd and Pt in terms of catalytic performance and resistance to aging are in good agreement with that reported in the literature to date. Fresh, Pt has a lower light-off (LO) temperature for all reactions. As for Pd, the LO temp increases slightly during the second run, due to either the more reduced state of the PGM, and/or due to sintering or other structural transformation which occurred during the first reaction. Pt achieves a higher maximum conversion CO conversion than Pd, however NO conversion are similar both in light-off profile and in maximum conversions achieved. Note that for both NO and HC conversion, Pt/CZ1 reaches maximum conversion almost immediately on light-off, unlike the more gradual incline for Pd/CZ1. The trend is slightly reversed on CZ2, with Pd/CZ2 performing slightly better, however the Pd and Pt are much more similar on this support. While the Pt/CZ2 light-off curve also shows the delay at approximately 180 °C for all CO, HC and NO conversion during run 1, which was attributed to changing metal-support interface for Pd/CZ2, the feature is much smaller for Pt compared to Pd.

While these results show that in many regards fresh Pt/CZ outperforms Pd/CZ, its limited effectiveness is observed immediately upon aging the samples under the same conditions the Pd samples were aged under (950°C, 12 h, in air). Light-off temperatures for all reactions increase by over 200°C for Pt/CZ1, and by over 150°C for Pt/CZ2. PXRD shows very large Pt metal nanoparticles, implying that significant levels of sintering are responsible for this activity loss. As demonstrated throughout this study, the redispersion mechanism of Pd/PdO on ceria zirconia is presumably responsible for its retained performance after aging, for example while Figure SI26.5 above shows that significant irreversible sintering of Pt^0 occurred during thermal aging, Figure 3c) in the main text showed that no metallic Pd was observed under the same conditions, reversibly reoxidising instead to PdO during cooling in air.

References

S1) Rietveld, H. M. A Profile Refinement Method for Nuclear and Magnetic Structures *J. Appl. Crystallogr.,* **1969**, 2, 65-71.

S2) Toby, B. H.; Von Dreele, R. B. GSAS-II: the genesis of a modern open-source all purpose crystallography software package, *J. Appl. Cryst.*, **2013**, 46, 544-549.

S3) Mccusker, L. B.; Von Dreele, R. B.; Cox, D. E.; Loue, D.; Scardi, P. Rietveld refinement guideline, *J. Appl. Crys.* **1999**, 32, 36-50.

S4) Summer, A.; Playford, H. Y.; Owen, L. R.; Fisher, J. M.; Kolpin, A.; Thompsett, D.; Walton, R. I. Order and disorder in cerium-rich ceria-zirconia solid solutions revealed from reverse Monte Carlo analysis of neutron and X-ray total scattering, *APL Mater.*, **2023**, 11, 031113.

S5) Yaremchenko, A. A.; Khalyavin, D. D.; Patrakeev, M. V. Uncertainty of oxygen content in highly nonstoichiometric oxides from neutron diffraction data: example of perovskite-type $Ba_{0.5}Sr_{0.5}Co_{0.8}Fe_{0.2}O_{3-\delta}$ *J. Mat. Chem. A* **2017**, 5**,** 3456-3463.

S6) Filik, J.; Ashton, A. W.; Chang, P. C. Y.; Chater, P. A.; Day, S. J.; Drakopoulos, M.; Gerring, M. W.; Hart, M. L.; Magdysyuk, O. V; Michalik, S.; Smith, A.; Tang, C. C.; Terrill, N. J.; Wharmby, M. T.; Wilhelm, H. Processing Two-Dimensional X-Ray Diffraction and Small-Angle Scattering Data in DAWN 2. *J. Appl. Cryst.* **2017**, 50, 959–966.

S7) Chupas, P. J.; Chapman, K. W.; Kurtz, C.; Hanson, J. C.; Lee, P. L.; Grey, C. P. A Versatile Sample-Environment Cell for Non-Ambient X-Ray Scattering Experiments. *J. Appl. Cryst.* **2008**, 41, 822–824.

S8) Thompson, P.; Bikondoa, O.; Bouchenoire, L.; Brown, S.; Cooper, M.; Hase, T.; Lucas, C.; Wermeille, D. New opportunities for the XMaS beamline arising from the ESRF upgrade program, *AIP Conf. Proc.*, **2019**, 2054, 060030.

S9) ESA Project Software by Mark Dowsett, https://warwick.ac.uk/fac/cross_fac/xmas/other_projects/esaproject accessed August 2023

S10) Hwang, A.; Wu, J.; Getsoian, A. "Bean"; Iglesia, E. Kinetic Relevance of Surface Reactions and Lattice Diffusion in the Dynamics of Ce−Zr Oxides Reduction−Oxidation Cycles, *J. Phys. Chem. C*, **2023**, 127, 2936-2952.

S11) Stubenrauch, J.; Vohs, J. M. Interaction of CO with Rh Supported on Stoichiometric and Reduced CeO_2(111) and CeO_2(100) Surfaces *J. Catal.*, **1996**, 159, 50-57.

S12) Hickey, N.; Fornasiero, P.; Kašpar, J.; Gatica, J. M.; Bernal, S. Effects of the Nature of the Reducing Agent on the Transient Redox Behavior of NM/$Ce_{0.68}Zr_{0.32}O_2$ (NMDPt, Pd, and Rh), *J. Catal.*, **2001**, 200, 181-193.

S13) Gredig, S.; Tagliaferri, S.; Maciejewski, M.; Baiker, A. Oxidation and disproportionation of carbon monoxide over Pd-ZrO_2 catalysts prepared from glassy Pd-Zr alloy and by coprecipitation, *Stud. Surf. Sci. Catal.*, **1995**, 96, 285-295.

S14) Matolín, V.; Matolínová, I.; Dvořák, F.; Johánek, V.; Mysliveček, J.; Prince, K. C.; Skála, T.; Stetsovych, O.; Tsud, N.; Václavů, M.; Šmíd, B. Water interaction with CeO_2(1 1 1)/Cu(1 1 1) model catalyst surface, *Catal. Today*, **2012**, 181, 124-132.

S15) Deguchi, H.; Yoshida, H.; Inagaki, T.; Horiuchi, M. EXAFS study of doped ceria using multiple data set fit, *Solid State Ionics,* **2005**, 176, 1817-1825.

S16) Nagai, Y.; Yamamoto, T.; Tanaka, T.; Yoshida, S.; Nonaka, T.; Okamoto, T.; Suda, A.; Sugiura, M. X-ray absorption fine structure analysis of local structure of CeO_2–ZrO_2 mixed oxides with the same composition ratio (Ce/Zr = 1), *Catal. Today*, **2002**, 74, 225-234.

S17) Ferraro, D.; Tredici, I. G.; Ghigna, P.; Castillio-Michel, H.; Falqui, A.; Di Benedetto, C.; Alberti, G.; Ricci, V.; Anselmi-Tamburini, U.; Sommi, P. Dependence of the Ce(III)/Ce(IV) ratio on intracellular localization in ceria nanoparticles internalized by human cells, *Nanoscale*, **2017**, 9, 1527-1538.

S18) Marchbank, H. R.; Clark, A. H.; Hyde, T. I.; Playford, H. Y.; Tucker, M. G.; Thompsett, D.; Fisher, J. M.; Chapman, K. W.; Beyer, K. A.; Monte, M.; Longo, A.; Sankar, G. Structure of Nano-sized CeO_2 Materials: Combined Scattering and Spectroscopic Investigations, *ChemPhysChem*, **2016**, 17, 3494-3503.

S19) Yashima, M.; Takashina, H.; Kakihana, M.; Yoshimura, M. Low-Temperature Phase Equilibria by the Flux Method and the Metastable–Stable Phase Diagram in the ZrO2–CeO2 System, *J. Am. Ceram. Soc.*, **1994**, 77, 1869-1874.

S20) Bozo, C.; Gaillard, F.; Guilhaume, N. Characterisation of ceria–zirconia solid solutions after hydrothermal ageing, *Appl. Catal. A*, **2001**, 220, 69-77.

S21) Lee, T. A.; Stanek, C. R.; McClellan, K. J.; Mitchell, J. N.; Navrotsky, A. Enthalpy of formation of the cubic fluorite phase in the ceria–zirconia system, *J. Mater. Res.*, **2008**, 23, 1105-1112.

S22) Loridant, S. Raman spectroscopy as a powerful tool to characterize ceria-based catalysts, *Catal. Today*, **2021**, 373, 98-111.

S23) Schneider, C. A.; Rasband, W. S.; Eliceiri, K. W. NIH Image to ImageJ: 25 Years of Image Analysis, *Nat Methods* **2012**, 9, 671–675.

S24) Artini, C.; Pani, M.; Carnasciali, M. M.; Buscaglia, M. T.; Plaisier, J. R.; Costa, G. A. Structural Features of Sm- and Gd-Doped Ceria Studied by Synchrotron X-ray Diffraction and μ-Raman Spectroscopy, *Inorg. Chem.*, **2015**, 54, 4126-4137.

S25) Bondars, B.; Heidemane, G.; Grabis, J.; Laschke, K.; Boysen, H.; Schneider, J.; Frey, F. Powder diffraction investigations of plasma sprayed zirconia, *J. Mater. Sci.*, **1995**, 30, 1621-1625.

S26) Ertl, G.; Knozinger, H.; Weitkamp, J. *Handbook of Heterogeneous Catalysis*; Wiley-VCH: Weinheim, Germany, 1997.

S27) Fernández-García, M.; Iglesias-Juez, A.; Martínez-Arias, A.; Hungría, A. B.; Anderson, J. A.; Conesa, J. C.; Soria, J. Role of the State of the Metal Component on the Light-off Performance of Pd-Based Three-Way Catalysts, *J. Catal.* **2004**, 221, 594–600.

S28) Sun, H. P.; Pan, X. P.; Graham, G. W.; Jen, H. W.; McCabe, R. W.; Thevuthasan, S.; Peden, C. H. F. Partial Encapsulation of Pd Particles by Reduced Ceria-Zirconia, *Appl. Phys. Lett.* **2005**, 87, 1–3

S29) Wang, T.; Zhou, R. PM-Support Interfacial Effect and Oxygen Mobility in Pt, Pd or Rh-Loaded (Ce,Zr,La)O2 Catalysts, *Front. Environ. Sci. Eng.* **2021**, 15, 76.

S30) Vlachou, M. C.; Marchbank, H. R.; Brooke, E.; Kolpin, A. Challenges and Opportunities for Platinum in the Modern Three-Way Catalyst: Flexibility and Performance in Gasoline Emissions Control. Johns. Matthey technol. rev. **2023**, 67, 219–229.

S31) Cooper, J.; Beecham, J. A Study of Platinum Group Metals in Three-Way Autocatalysts. *Platin. Met. Rev*. **2013**, 57, 281–288.

S32) Theis, J. R. An Assessment of Pt and Pd Model Catalysts for Low Temperature NOx Adsorption. *Catal. Today,* **2016**, 267, 93–109.
